# Supplementary material for: Experimental Estimation of the Effects of All Amino-Acid Mutations to HIV’s Envelope Protein on Viral Replication in Cell Culture
Source: PLoS Pathog. 2016 Dec 13;12(12):e1006114. doi: 10.1371/journal.ppat.1006114 (PMC5189966; doi:10.1371/journal.ppat.1006114)
Supplement: S1 Table — The 25 sites (HXB2 numbering) from Fig 2B for which the error-corrected mutation frequency increased by >3-fold in at least two replicates upon two rounds of passaging in cell culture. We report the change in mutation frequency for each site as a ratio of mutation frequency post- vs. pre- selection (P2:DNA). Negative ratios arise when the mutation frequency in the wildtype control is greater than in the mutant plasmid or virus library. For many sites, there is a large difference between the hydropathy of the wildtype amino acid and the hydropathy averaged across the site’s amino-acid preferences, suggesting pressure to change the chemical character of the amino-acid. We also report the relative solvent accessibility for each site as computed using PDB structure 4TVP [133]. Adaptation at each site could occur through a single highly beneficial amino-acid change or though numerous roughly equally beneficial changes. For many sites, we observe the latter scenario, as indicated by the entropy of the preferences, which ranges from 2.2–4.1 in this list of 25 sites, compared to 0.5–4.3 for all sites. (PDF) [file ppat.1006114.s001.pdf]

sites	error-corrected mutation frequency (P2:DNA)			WT amino acid	hydropathy			RSA	entropy of preferences
	1	2	3		WT amino acid	preferences	difference		
48	3.4	2.1	3.4	A	1.8	-0.4	2.2	0.2	2.9
62	1.6	4.2	8.2	D	-3.5	-1.6	-1.9	0.6	3.6
64	14.4	6.8	10.3	E	-3.5	-1.6	-1.9	0.6	2.8
65	1.3	3.3	3.1	V	4.2	-2.1	6.3	0.6	3.3
66	6.1	3.0	13.7	H	-3.2	-0.2	-3.0	0.6	3.5
81	4.1	4.5	4.1	P	-1.6	-1.9	0.3	0.6	2.9
105	2.2	3.1	7.0	H	-3.2	0.7	-3.9	0.0	3.0
162	11.2	4.9	1.2	S	-0.8	-2.6	1.8	0.2	2.7
188	5.8	2.9	4.8	T	-0.7	-2.1	1.4	0.5	2.9
203	-8.6	6.7	5.4	Q	-3.5	0.0	-3.5	0.0	2.5
207	15.6	19.2	21.1	K	-3.9	2.7	-6.6	0.5	2.7
212	5.2	-27.2	9.8	P	-1.6	-0.3	-1.3	0.2	3.4
377	10.5	1.1	3.9	N	-3.5	-2.7	-0.8	0.2	2.2
420	3.0	3.8	4.8	I	4.5	-1.7	6.2	0.0	2.8
433	7.2	7.2	8.4	A	1.8	2.2	-0.4	0.0	2.2
436	3.1	2.8	5.2	A	1.8	1.1	0.7	0.0	2.4
443	-5.4	4.2	3.2	I	4.5	-0.9	5.4	0.1	3.4
557	4.7	11.4	5.6	R	-4.5	0.6	-5.1	nd	3.5
558	2.9	6.0	3.8	A	1.8	-0.7	2.5	nd	2.2
560	5.9	5.2	6.0	E	-3.5	0.7	-4.2	nd	3.2
564	42.5	6.3	-4.0	H	-3.2	-0.8	-2.4	nd	4.1
588	9.4	11.4	11.2	K	-3.9	1.5	-5.4	0.2	3.0
591	6.2	4.2	5.0	Q	-3.5	2.2	-5.7	0.0	2.3
653	1.6	3.4	3.2	Q	-3.5	1.7	-5.2	0.5	3.1
655	10.8	8.2	4.7	K	-3.9	1.5	-5.4	0.1	3.3
