# Supplementary material for: Experimental Estimation of the Effects of All Amino-Acid Mutations to HIV’s Envelope Protein on Viral Replication in Cell Culture
Source: PLoS Pathog. 2016 Dec 13;12(12):e1006114. doi: 10.1371/journal.ppat.1006114 (PMC5189966; doi:10.1371/journal.ppat.1006114)
Supplement: S2 Table — This table shows all sites (in HXB2 numbering) that differ between HXB2 and LAI for which we have estimates of Env’s preferences. At each site, we report the wildtype amino-acid identity for each strain and its corresponding preference from Fig 5. Most sites favor the HXB2 identity more than the LAI identity. Three sites (137, 192, and 275) strongly prefer the HXB2 identity, while only a single (626) site strongly prefers the LAI identity. (PDF) [file ppat.1006114.s002.pdf]

site	HXB2 identity	LAI identity	HXB2 preference	LAI preference	difference (=HXB2-LAI)
135	K	G	0.047	0.046	0.001
137	D	A	0.202	0.043	0.159
146	R	E	0.083	0.054	0.028
148	I	M	0.118	0.038	0.080
192	K	T	0.316	0.021	0.295
275	V	A	0.203	0.043	0.160
290	T	Q	0.110	0.036	0.074
306	R	S	0.027	0.043	-0.016
340	N	A	0.024	0.055	-0.030
423	I	F	0.250	0.186	0.064
429	K	E	0.085	0.006	0.078
461	S	N	0.074	0.039	0.035
464	E	G	0.133	0.051	0.082
625	H	N	0.028	0.041	-0.013
626	T	M	0.155	0.281	-0.126
684	L	I	0.028	0.048	-0.021
