# Supplementary material for: Experimental Estimation of the Effects of All Amino-Acid Mutations to HIV’s Envelope Protein on Viral Replication in Cell Culture
Source: PLoS Pathog. 2016 Dec 13;12(12):e1006114. doi: 10.1371/journal.ppat.1006114 (PMC5189966; doi:10.1371/journal.ppat.1006114)
Supplement: S3 Table — A multiple linear regression as in Table 3, except the five variable loops (V1–V5) are analyzed independently from one another. (PDF) [file ppat.1006114.s003.pdf]

variable	coefficient	95% confidence interval
RSA	1.37	1.04 to 1.70
RRE	-0.80	-0.98 to -0.62
bNAbs	-0.16	-0.28 to -0.05
V1	0.09	-0.28 to 0.46
V2	-0.13	-0.39 to 0.13
V3	0.15	-0.13 to 0.42
V4	0.32	-0.03 to 0.66
V5	0.28	-0.21 to 0.77
