# Supplementary figures and images for: Experimental Estimation of the Effects of All Amino-Acid Mutations to HIV’s Envelope Protein on Viral Replication in Cell Culture
Source: PLoS Pathog. 2016 Dec 13;12(12):e1006114. doi: 10.1371/journal.ppat.1006114 (PMC5189966; doi:10.1371/journal.ppat.1006114)

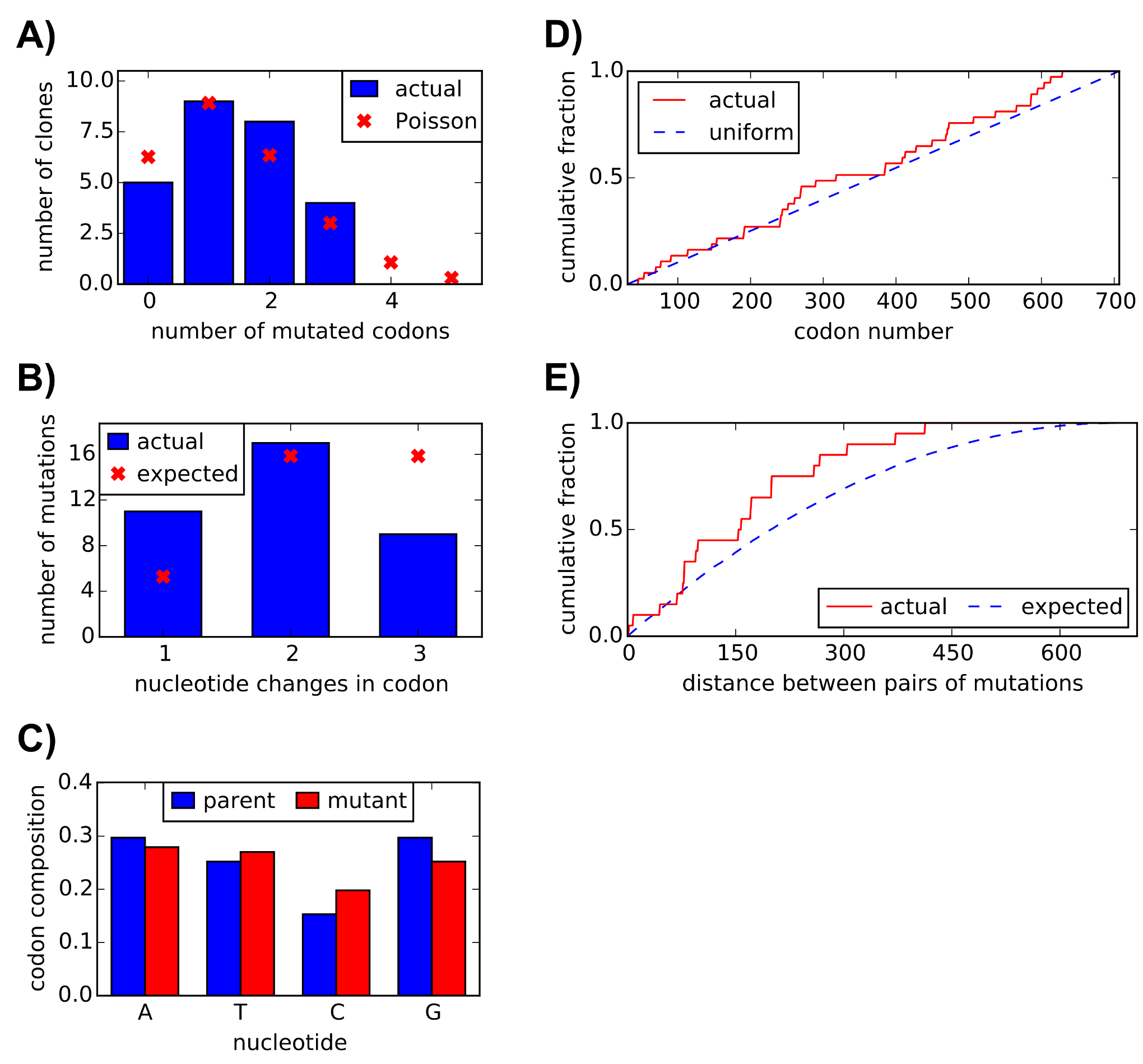

Supplement: S1 Fig — We Sanger sequenced 26 clones sampled roughly evenly from the three replicate mutant plasmid libraries prior to any functional selection. (A) We observed an average of 1.4 mutant codons per clone. The number of mutant codons per clone closely followed a Poisson distribution. (B) Mutant codons had a mix of single-, double-, and triple-nucleotide changes. (C) The nucleotide frequencies were fairly uniform in the mutant codons. (D) Mutations were distributed roughly evenly along the portion of env that we mutagenized (codons 31–707). (E) For clones with multiple mutations, we computed pairwise distances between mutations in primary sequence and plotted the cumulative distribution of these distances (red line). For comparison, we simulated the expected distribution of pairwise distances if mutations occurred entirely independently (blue line). The difference between the actual and expected distributions suggests our mutagenesis had a slight bias to introduce mutations closer together than expected by chance. (TIFF) [file ppat.1006114.s004.tiff]

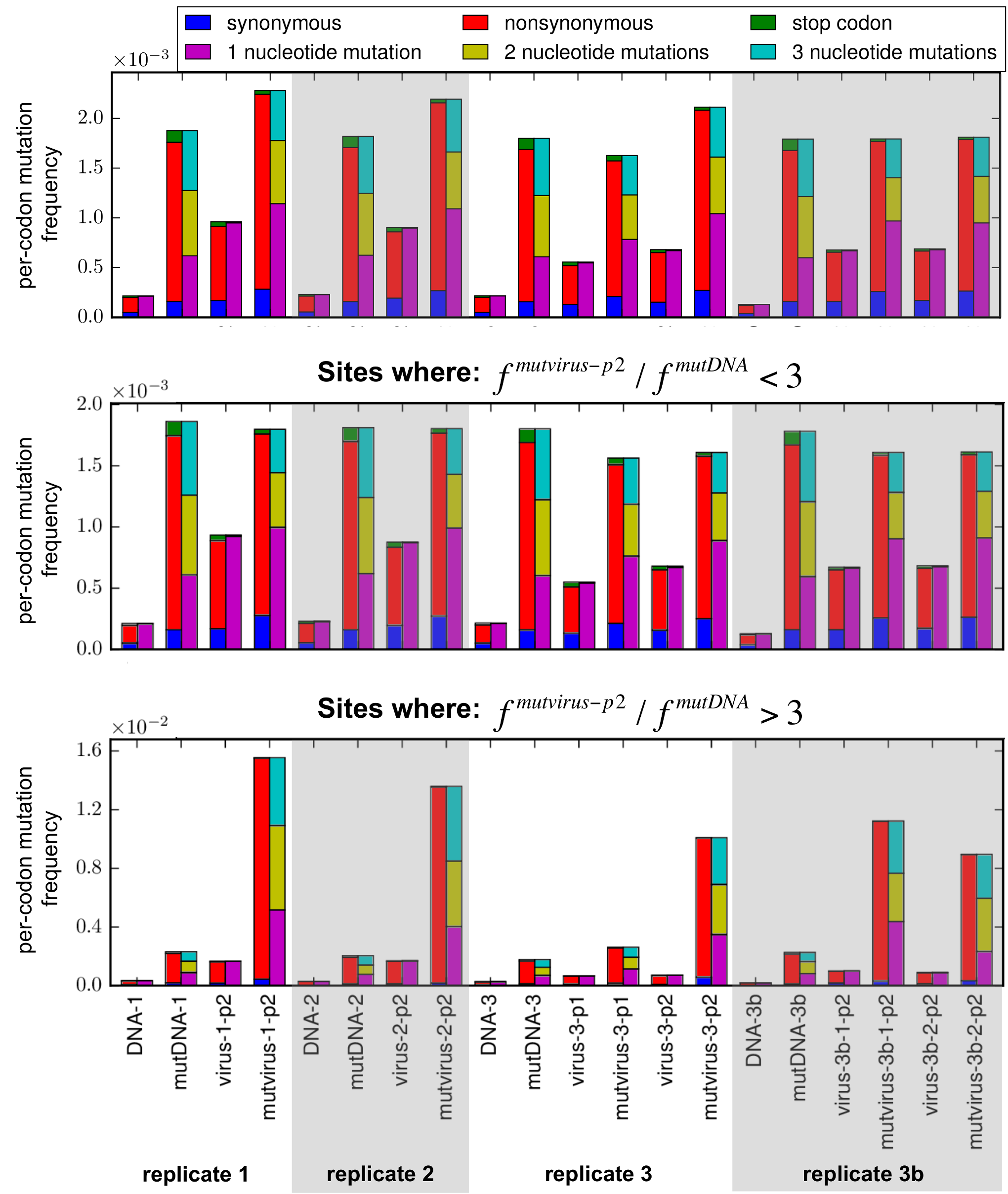

Supplement: S2 Fig — This figure is similar to Fig 2 except that it shows the uncorrected mutation frequencies in the mutant plasmid and mutant virus libraries, and the mutation frequencies in the wildtype controls that were used to correct the mutation frequencies in Fig 2. Codon mutations are classified both by their effect on the protein (synonymous, nonsynonymous, or stop codon) and by the number of nucleotides they change in the codon (one, two, or three). The top panel shows data for all sites, whereas the middle and lower panels show data for the indicated subsets of sites. This this plot, fmutvirus−p2 and fmutDNA refer to the nonsynonymous mutation frequency in the twice-passaged mutant viruses and the initial mutant DNA, respectively. (TIFF) [file ppat.1006114.s005.tiff]

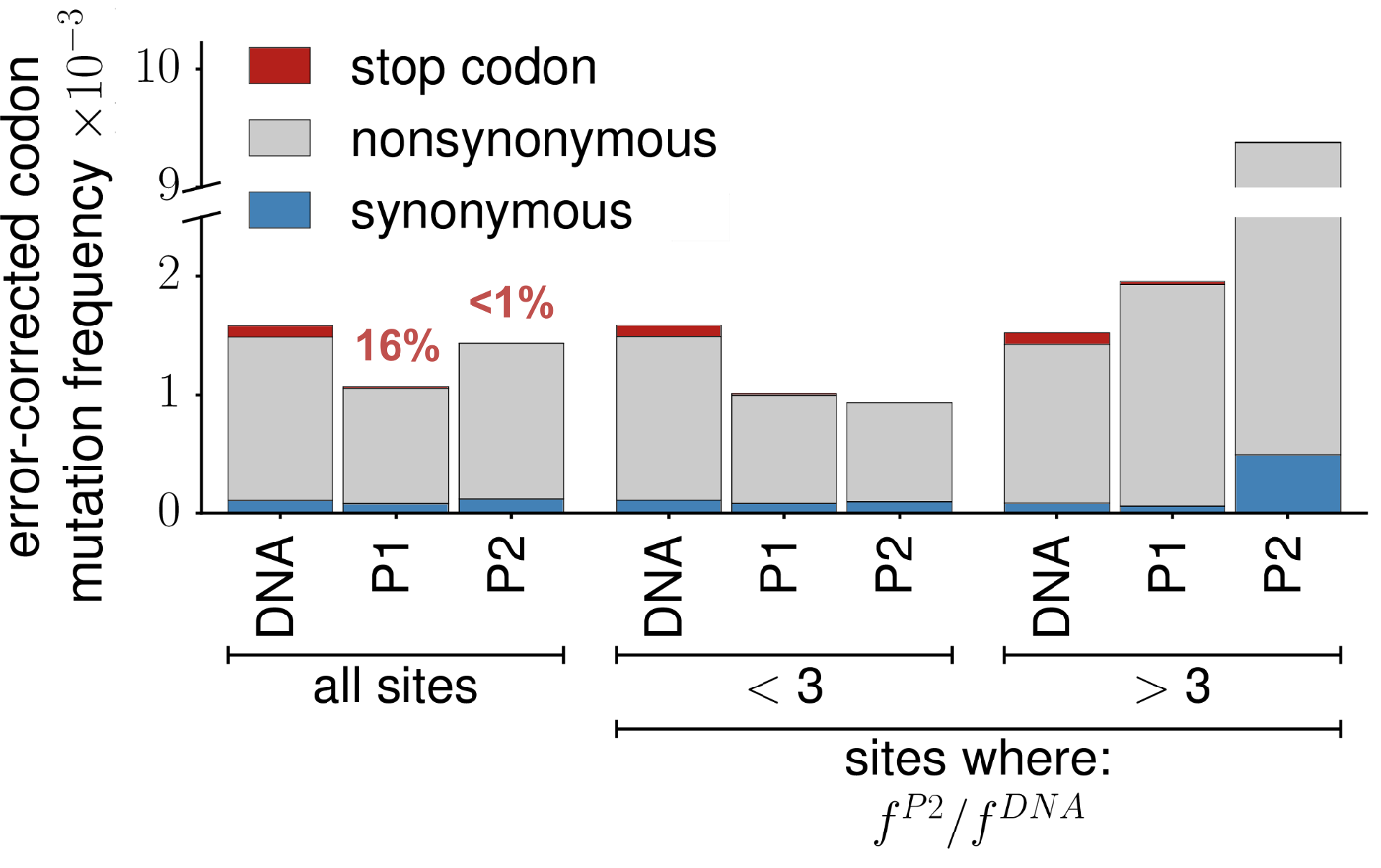

Supplement: S3 Fig — We deep sequenced the replicate 3 library after both one (P1) and two (P2) rounds of viral passaging. This figure is similar to Fig 2, but shows data for both P1 and P2. Purging of stop-codon mutations shows selection was only complete after two rounds of passaging. Whereas two rounds of passaging purged stop-codon mutations to <1% their frequency in the initial library (DNA), one round of passaging only purged stop-codon mutations to 16% their starting frequency (see the data for “all sites”, where the red numbers above the bars for P1 and P2 indicate the percentage of stop codons after each passage relative to the starting library). (TIFF) [file ppat.1006114.s006.tiff]

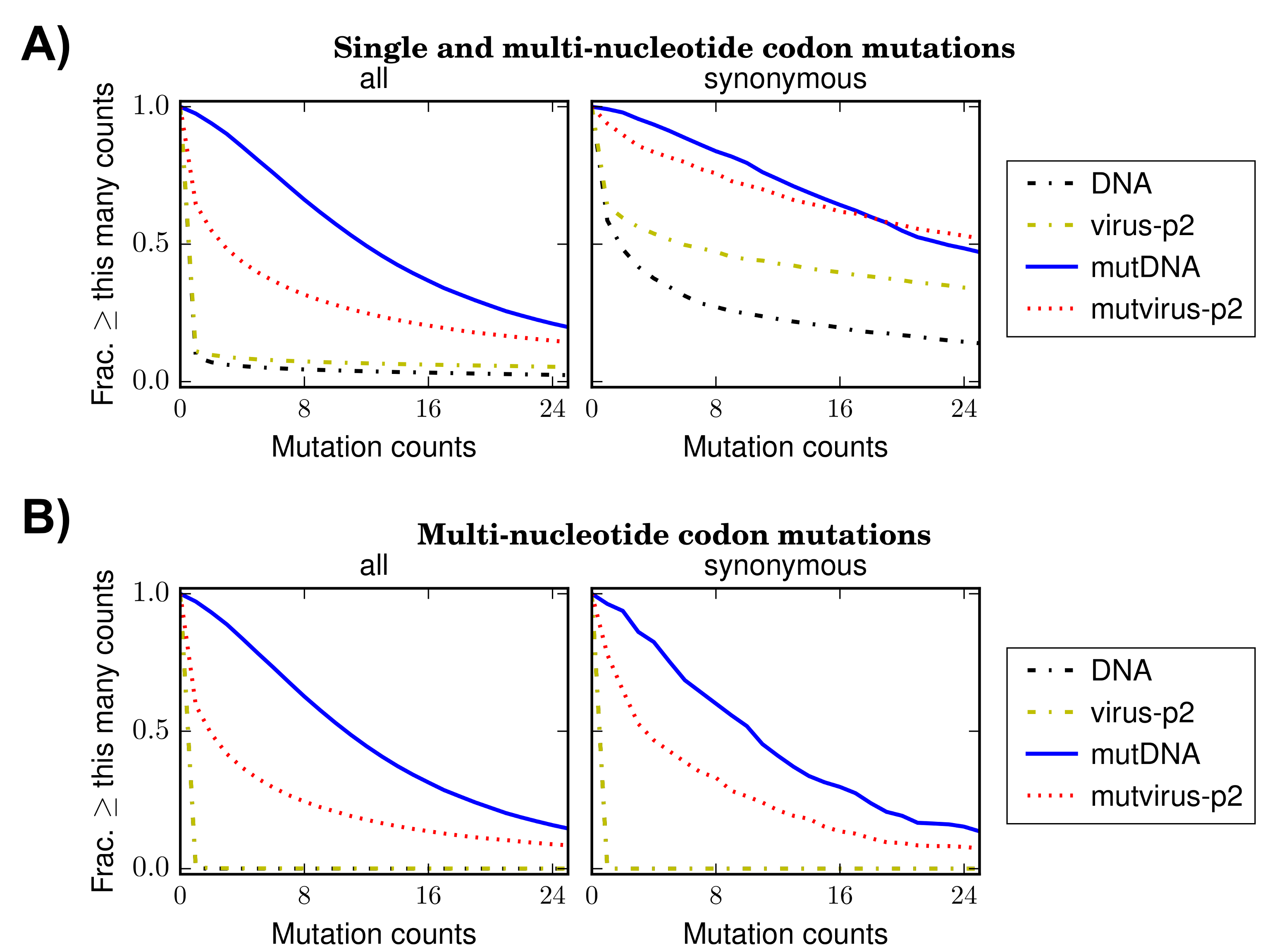

Supplement: S4 Fig — (A) Each plot shows the number of all (single and multi-nucleotide) codon mutations observed at least the indicated number of times in the sequencing of all replicates combined. We observed almost all mutations in the starting plasmid libraries (mutDNA), showing rich initial mutational diversity. Many mutations were depleted in the mutant virus libraries after two rounds of passaging (mutvirus-p2), consistent with purifying selection purging deleterious variants or bottlenecking diminishing library diversity. Examination of mutation counts in the wildtype plasmid (DNA) and wildtype virus (virus-p2) controls revealed a considerable fraction of mutations that were present at appreciable numbers due to errors from deep sequencing and PCR or de novo mutations from viral replication. This observation underscores the importance of using these wildtype controls to correct for background errors and de novo mutations. (B) If we examine only multi-nucleotide codon mutations, then there are negligible background errors in the wildtype controls. Similar data for each replicate individually are in S5 Fig. (TIFF) [file ppat.1006114.s007.tiff]

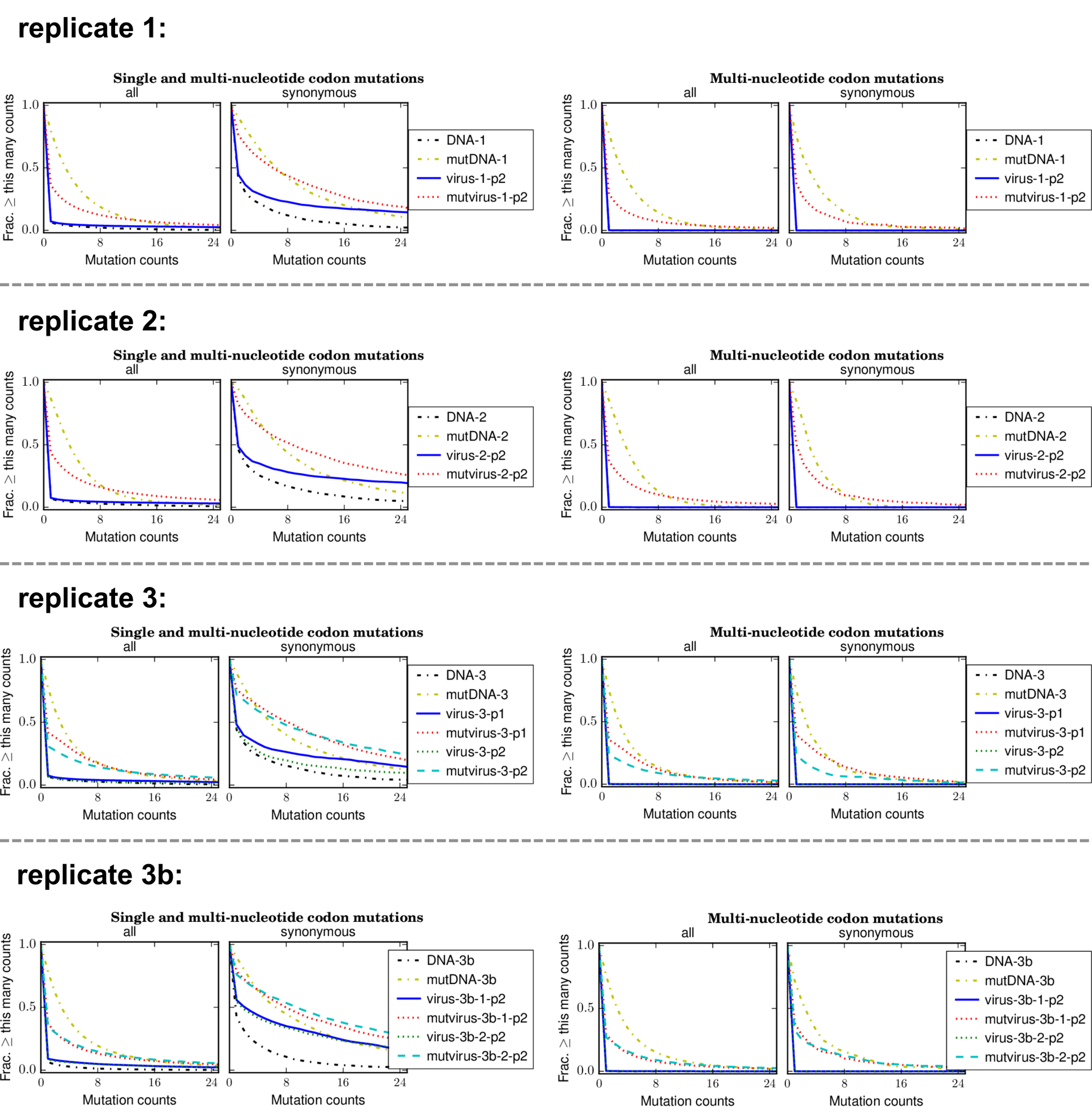

Supplement: S5 Fig — These plots are the same as in S4 but show each replicate individually. (TIFF) [file ppat.1006114.s008.tiff]

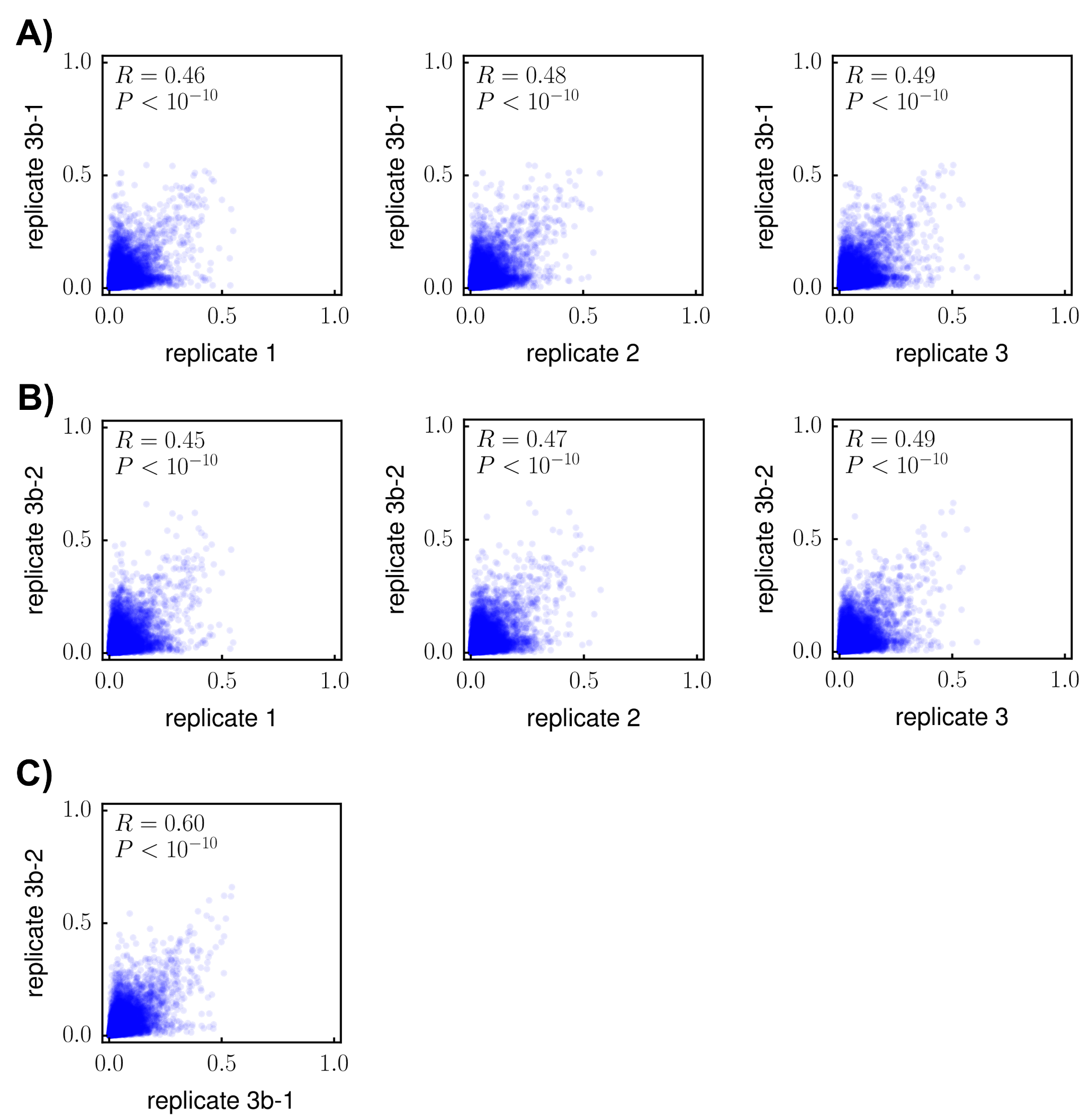

Supplement: S6 Fig — (A) The correlation between replicate 3b-1 and replicate 1, 2, or 3. (B) The correlation between replicate 3b-2 and replicate 1, 2, or 3. (C) The correlation between replicates 3b-1 and 3b-2. (TIFF) [file ppat.1006114.s009.tiff]

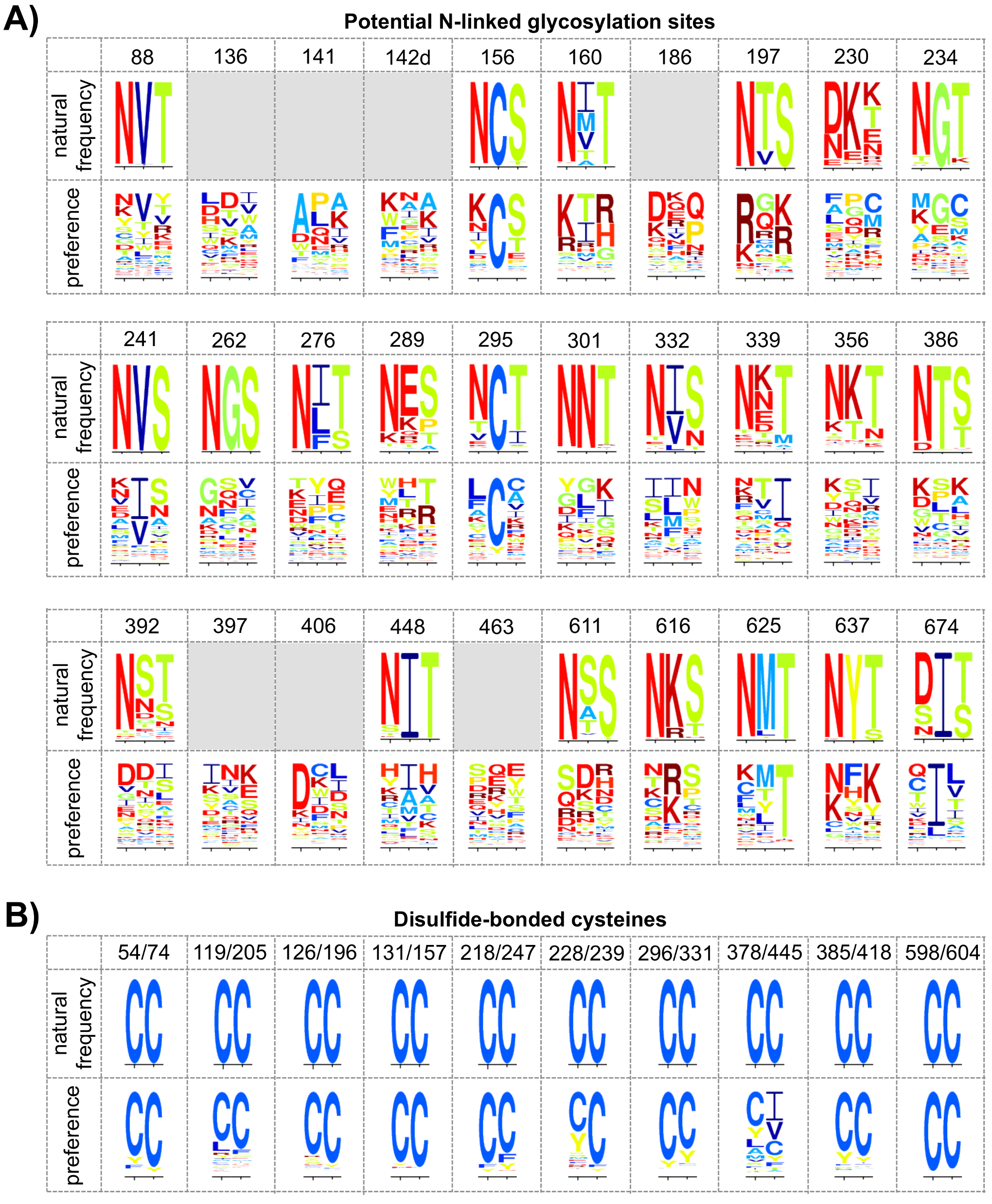

Supplement: S7 Fig — This figure is similar to Fig 8, but shows logo plots for all 30 glycosylation sites (defined using the N-GlycoSite tool [134] from the HIV sequence database, http://www.hiv.lanl.gov/) and all 10 disulfide bonds [135] in LAI. (A) Most glycosylation sites are highly conserved in natural sequences, but highly tolerant of mutations in our experiments. Logo plots showing amino-acid frequencies in nature are replaced by grey boxes for sites in the alignment of group-M sequences that were masked because the site had >5% deletions relative to HXB2 or because the region looked unalignable by eye (for details, see IPython notebook CurateLANLMultipleSequenceAlignment.ipynb within S3 File). (B) Disulfide-bonded cysteines are absolutely conserved in nature. Most of these positions have a strong preference for cysteine in our experiments. A previous study [49] found that only the C378-C445 disulfide bond tolerated alanine mutations at individual cysteines while supporting robust viral replication in cell culture. In accordance with this previous work, these cysteines are the most mutationally tolerant ones in our experiment. (TIFF) [file ppat.1006114.s010.tiff]
