# Supplementary material for: Experimental Estimation of the Effects of All Amino-Acid Mutations to HIV’s Envelope Protein on Viral Replication in Cell Culture
Source: PLoS Pathog. 2016 Dec 13;12(12):e1006114. doi: 10.1371/journal.ppat.1006114 (PMC5189966; doi:10.1371/journal.ppat.1006114)
Supplement: S3 File — (ZIP) [file ppat.1006114.s013.zip › S3_File_notebooks/AlignSequencingReadsAndComputeMutationalCounts.html]

AlignSequencingReadsAndComputeMutationalCounts


# Align sequencing reads to gene and compute mutational counts¶

## Contents¶

Imports

Write input files

Global variables

Align sequencing reads and make counts files

Make plots summarizing the alignments

Merge counts between replicates

Analyze mutational counts for sites both within and outside of the Rev-response element

## Imports¶

In [1]:

```
import os
import glob
from IPython.display import Image, display
```

## Write input files¶

- *LAI\_env.fasta*: is a FASTA file of LAI *env* codons 1-709. The deep mutational scan only included codons 31-707 (capitalized letters). Codons 1-30 are included here so that `dms_barcodedsubamplicons` uses the correct numbering. Codons 708 and 709 are included here since they are part of one of the subamplicons. I will remove codons 1-30, 708, and 709 from counts files before inferring the preferences.

In [2]:

```
%%writefile LAI_env.fasta
>LAI env codons 1-709 
atgagagtgaaggagaaatatcagcacttgtggagatgggggtggaaatggggcaccatgctccttgggatattgatgatctgtagtgctACAGAAAAATTGTGGGTCACAGTCTATTATGGGGTACCTGTGTGGAAGGAAGCAACCACCACTCTATTTTGTGCATCAGATGCTAAAGCATATGATACAGAGGTACATAATGTTTGGGCCACACATGCCTGTGTACCCACAGACCCCAACCCACAAGAAGTAGTATTGGTAAATGTGACAGAAAATTTTAACATGTGGAAAAATGACATGGTAGAACAGATGCATGAGGATATAATCAGTTTATGGGATCAAAGCCTAAAGCCATGTGTAAAATTAACCCCACTCTGTGTTAGTTTAAAGTGCACTGATTTGGGGAATGCTACTAATACCAATAGTAGTAATACCAATAGTAGTAGCGGGGAAATGATGATGGAGAAAGGAGAGATAAAAAACTGCTCTTTCAATATCAGCACAAGCATAAGAGGTAAGGTGCAGAAAGAATATGCATTTTTTTATAAACTTGATATAATACCAATAGATAATGATACTACCAGCTATACGTTGACAAGTTGTAACACCTCAGTCATTACACAGGCCTGTCCAAAGGTATCCTTTGAGCCAATTCCCATACATTATTGTGCCCCGGCTGGTTTTGCGATTCTAAAATGTAATAATAAGACGTTCAATGGAACAGGACCATGTACAAATGTCAGCACAGTACAATGTACACATGGAATTAGGCCAGTAGTATCAACTCAACTGCTGTTGAATGGCAGTCTAGCAGAAGAAGAGGTAGTAATTAGATCTGCCAATTTCACAGACAATGCTAAAACCATAATAGTACAGCTGAACCAATCTGTAGAAATTAATTGTACAAGACCCAACAACAATACAAGAAAAAGTATCCGTATCCAGAGGGGACCAGGGAGAGCATTTGTTACAATAGGAAAAATAGGAAATATGAGACAAGCACATTGTAACATTAGTAGAGCAAAATGGAATGCCACTTTAAAACAGATAGCTAGCAAATTAAGAGAACAATTTGGAAATAATAAAACAATAATCTTTAAGCAATCCTCAGGAGGGGACCCAGAAATTGTAACGCACAGTTTTAATTGTGGAGGGGAATTTTTCTACTGTAATTCAACACAACTGTTTAATAGTACTTGGTTTAATAGTACTTGGAGTACTGAAGGGTCAAATAACACTGAAGGAAGTGACACAATCACACTCCCATGCAGAATAAAACAATTTATAAACATGTGGCAGGAAGTAGGAAAAGCAATGTATGCCCCTCCCATCAGCGGACAAATTAGATGTTCATCAAATATTACAGGGCTGCTATTAACAAGAGATGGTGGTAATAACAACAATGGGTCCGAGATCTTCAGACCTGGAGGAGGAGATATGAGGGACAATTGGAGAAGTGAATTATATAAATATAAAGTAGTAAAAATTGAACCATTAGGAGTAGCACCCACCAAGGCAAAGAGAAGAGTGGTGCAGAGAGAAAAAAGAGCAGTGGGAATAGGAGCTTTGTTCCTTGGGTTCTTGGGAGCAGCAGGAAGCACTATGGGCGCAGCGTCAATGACGCTGACGGTACAGGCCAGACAATTATTGTCTGGTATAGTGCAGCAGCAGAACAATTTGCTGAGGGCTATTGAGGCGCAACAGCATCTGTTGCAACTCACAGTCTGGGGCATCAAGCAGCTCCAGGCAAGAATCCTGGCTGTGGAAAGATACCTAAAGGATCAACAGCTCCTGGGGATTTGGGGTTGCTCTGGAAAACTCATTTGCACCACTGCTGTGCCTTGGAATGCTAGTTGGAGTAATAAATCTCTGGAACAGATTTGGAATAACATGACCTGGATGGAGTGGGACAGAGAAATTAACAATTACACAAGCTTAATACATTCCTTAATTGAAGAATCGCAAAACCAGCAAGAAAAGAATGAACAAGAATTATTGGAATTAGATAAATGGGCAAGTTTGTGGAATTGGTTTAACATAACAAATTGGCTGTGGTATATAAAAATATTCATAATGATAGTAGGAGGCTTGGTAGGTTTAAGAATAGTTTTTGCTGTACTTtctata
```

```
Overwriting LAI_env.fasta
```

- *remove\_unmutated\_codons.txt*: is an input file for `dms_editsites` for removing codons 1-30, 708, and 709 from counts files. See above for reason for removal.

In [3]:

```
%%writefile remove_unmutated_codons.txt
# Codons to remove
1
2
3
4
5
6
7
8
9
10
11
12
13
14
15
16
17
18
19
20
21
22
23
24
25
26
27
28
29
30
708
709
```

```
Overwriting remove_unmutated_codons.txt
```

## Global variables¶

In [4]:

```
# Replicates and samples
replicates = [1, 2, 3, '3b']
samples = {}
samples[1] = ['DNA-1', 'mutDNA-1', 'virus-1-p2', 'mutvirus-1-p2']
samples[2] = ['DNA-2', 'mutDNA-2', 'virus-2-p2', 'mutvirus-2-p2']
samples[3] = ['DNA-3', 'mutDNA-3', 'virus-3-p1', 'mutvirus-3-p1', 'virus-3-p2', 'mutvirus-3-p2']
samples['3b'] = ['DNA-3b', 'mutDNA-3b', 'virus-3b-1-p2', 'mutvirus-3b-1-p2', 'virus-3b-2-p2', 'mutvirus-3b-2-p2']

# Reference sequence
refseq = 'LAI_env.fasta'

# Input file for dms_editsites
remove_unmutated_codons_file = 'remove_unmutated_codons.txt'

# Alignment specs
alignspecs = '90,433,39,39 421,769,38,35 765,1116,35,33 1117,1425,38,35 1424,1779,35,33 1780,2127,38,38'
```

## Download the sequencing data from the Sequence Read Archive¶

Here is a file with the accession numbers to download sequencing data for each sample from the Sequence Read Archive (http://www.ncbi.nlm.nih.gov/sra). We named samples using the convention in the following examples:

- "mutDNA-1-run-1" denotes the first sequencing run for the mutant plasmid library for replicate 1 (each sample has FASTQ files for between 2-3 MiSeq sequencing runs)
- "DNA-1-run-2" denotes the second sequencing run of the wild-type plasmid for replicate 1
- "mutvirus-p2-1-run-1" denotes the first sequencing run of the twice-passaged mutant viral libraries for replicate 1
- "virus-p2-3b-1-run-1" denotes the first sequencing run of the twice-passaged wild-type virus for replicate 3b-1.:

In [5]:

```
%%writefile upload_data_to_SRA/SRA_accession_numbers.txt
DNA-1-run-1	SRR3724872
DNA-1-run-2	SRR3724873
DNA-1-run-3	SRR3724874
virus-1-p2-run-1	SRR3724878
virus-1-p2-run-2	SRR3724879
virus-1-p2-run-3	SRR3724880
DNA-2-run-1	SRR3724884
DNA-2-run-2	SRR3724885
DNA-2-run-3	SRR3724886
virus-2-p2-run-1	SRR3724890
virus-2-p2-run-2	SRR3724891
virus-2-p2-run3	SRR3724892
DNA-3-run-1	SRR3724896
DNA-3-run-2	SRR3724897
DNA-3-run-3	SRR3724898
virus-3-p1-run-1	SRR3724902
virus-3-p1-run-2	SRR3724903
virus-3-p1-run-3	SRR3724904
virus-3-p2-run-1	SRR3724908
virus-3-p2-run-2	SRR3724909
virus-3-p2-run-3	SRR3724910
DNA-3b-run-1	SRR3724914
DNA-3b-run-2	SRR3724915
virus-3b-1-p2-run-1	SRR3724918
virus-3b-1-p2-run-2	SRR3724919
virus-3b-2-p2-run-1	SRR3724922
virus-3b-2-p2-run-2	SRR3724923
mutDNA-1-run-1	SRR3724875
mutDNA-1-run-2	SRR3724876
mutDNA-1-run-3	SRR3724877
mutvirus-1-p2-run-1	SRR3724881
mutvirus-1-p2-run-2	SRR3724882
mutvirus-1-p2-run-3	SRR3724883
mutDNA-2-run-1	SRR3724887
mutDNA-2-run-2	SRR3724888
mutDNA-2-run-3	SRR3724889
mutvirus-2-p2-run-1	SRR3724893
mutvirus-2-p2-run-2	SRR3724894
mutvirus-2-p2-run-3	SRR3724895
mutDNA-3-run-1	SRR3724899
mutDNA-3-run-2	SRR3724900
mutDNA-3-run-3	SRR3724901
mutvirus-3-p1-run-1	SRR3724905
mutvirus-3-p1-run-2	SRR3724906
mutvirus-3-p1-run-3	SRR3724907
mutvirus-3-p2-run-1	SRR3724911
mutvirus-3-p2-run-2	SRR3724912
mutvirus-3-p2-run-3	SRR3724913
mutDNA-3b-run-1	SRR3724916
mutDNA-3b-run-2	SRR3724917
mutvirus-3b-1-p2-run-1	SRR3724920
mutvirus-3b-1-p2-run-2	SRR3724921
mutvirus-3b-2-p2-run-1	SRR3724924
mutvirus-3b-2-p2-run-2	SRR3724925
```

```
Overwriting upload_data_to_SRA/SRA_accession_numbers.txt
```

Next, I will download FASTQ files from the SRA. (Note: In adapting this code, be aware that the `ftp` path I use will probably be different for your data, specifically at the subdirectory `../SRR366/..`, which you can probably just modify based on the first six characters of your file-specific accession numbers [e.g. SRR366]).

In [6]:

```
%%capture

# Read in samples and corresponding accession numbers
SRA_accession_numbers = []
with open('upload_data_to_SRA/SRA_accession_numbers.txt') as f:
    for line in f.readlines():
        SRA_accession_numbers.append(line.strip().split())

# Make a directory for the FASTQ files
FASTQdir = 'FASTQ_files/'
if not os.path.isdir(FASTQdir):
    os.makedirs(FASTQdir)
        
# Download FASTQ files from the SRA
for (samplename, accession) in SRA_accession_numbers:
    print "\nDownloading and converting file format for sample %s, which corresponds to accession %s." % (samplename, accession)
    log = !cd FASTQ_files; wget --no-clobber 'ftp://ftp-trace.ncbi.nih.gov/sra/sra-instant/reads/ByRun/sra/SRR/SRR372/'$accession'/'$accession'.sra'
    log = !mv "FASTQ_files/"$accession".sra" "FASTQ_files/"$samplename".sra"
    log = !fastq-dump -O FASTQ_files/ --split-files "FASTQ_files/"$samplename".sra"
```

## Align sequencing reads and make counts files¶

First, I use `dms_barcodedsubamplicons` to align deep-sequencing data to the LAI *env* gene and create mutational counts files.

In [7]:

```
print "The alignments and counts files will be made using:"
!dms_barcodedsubamplicons -v

print "\nThe counts files will be edited using:"
!dms_editsites -v

# Dictionary keyed by replicate and values corresponding to input for dms_summarizealignments
alignprefixes_names = {}
# Dictionary keyed by replicate and then by sample, returning the corresponding edited counts file
edited_counts_files = {}

# For each replicate, make the alignments, and then edit counts files, removing codons 1-30, 708 and 709
for replicate in replicates:
    print "\nAnalyzing replicate: %s" %replicate
    alignprefixes_names[replicate] = []
    edited_counts_files[replicate] = {}
    for sample in samples[replicate]:
        print "\nAnalyzing sample: %s" %sample
        
        # Make a list of R1 and R2 FASTQ files for the sample
        r1files = glob.glob('%s%s-run*_1.fastq' % (FASTQdir, sample))
        r1files.sort()
        r2files = glob.glob('%s%s-run*_2.fastq' % (FASTQdir, sample))
        r2files.sort()

        # Create directories for each replicate and sample, and specify file prefixes
        alignmentdir = 'replicate-%s/%s/' %(replicate, sample)
        if not os.path.isdir(alignmentdir):
            os.makedirs(alignmentdir)
        alignprefix = '%s%s_' %(alignmentdir, sample)
        edited_alignprefix = '%s%s_edited_' %(alignmentdir, sample)
        alignprefixes_names[replicate].append("%s,%s"%(edited_alignprefix, sample))
        countsfile = '%scounts.txt' %alignprefix
        edited_countsfile = '%scounts.txt' %edited_alignprefix
        statsfile = '%ssummarystats.txt' %alignprefix 
        edited_statsfile = '%ssummarystats.txt' %edited_alignprefix # same as statsfile, but with the edited_alignprefix extension that matches the edited_countsfile, which is required for alignmentsummaries to work properly
        
        # Add edited codon counts files to a dictionary for merging counts between replicates later on
        edited_counts_files[replicate][sample] = edited_countsfile
        
        # Filter and align sequencing reads using dms_barcodedsubamplicons
        cmd_barcodedsubamplicons = ' '.join([
                'dms_barcodedsubamplicons',
                alignprefix,
                refseq,
                ','.join(r1files),
                ','.join(r2files),
                alignspecs,
                '--R1trimlength 231',
                '--R2trimlength 191',
                '--barcodeinfo'])
        print ("\nAligning sequencing reads and making mutation counts files with the command:\n" + cmd_barcodedsubamplicons)
        log = !$cmd_barcodedsubamplicons
        
        # Edit the counts files, removing codons 1-30, 708 and 709
        cmd_editsites = ' '.join([
                'dms_editsites',
                countsfile, # input counts file
                edited_countsfile, # output counts file
                'remove', # edit command
                remove_unmutated_codons_file]) # renumbering file
        print ("\nRemoving unmutated sites with the command:\n" + cmd_editsites)
        log = !$cmd_editsites
        
        # Make a new statsfile with the same suffix as the corresponding edited counts file
        cmd_cp = 'cp %s %s'%(statsfile, edited_statsfile)
        print ("\nMaking a renamed version of the stats file with:\n" + cmd_cp)
        log = !$cmd_cp
```

```
The alignments and counts files will be made using:
dms_barcodedsubamplicons 1.1.dev16

The counts files will be edited using:
dms_editsites 1.1.dev16

Analyzing replicate: 1

Analyzing sample: DNA-1

Aligning sequencing reads and making mutation counts files with the command:
dms_barcodedsubamplicons replicate-1/DNA-1/DNA-1_ LAI_env.fasta FASTQ_files/DNA-1-run-1_1.fastq,FASTQ_files/DNA-1-run-2_1.fastq,FASTQ_files/DNA-1-run-3_1.fastq FASTQ_files/DNA-1-run-1_2.fastq,FASTQ_files/DNA-1-run-2_2.fastq,FASTQ_files/DNA-1-run-3_2.fastq 90,433,39,39 421,769,38,35 765,1116,35,33 1117,1425,38,35 1424,1779,35,33 1780,2127,38,38 --R1trimlength 231 --R2trimlength 191 --barcodeinfo

Removing unmutated sites with the command:
dms_editsites replicate-1/DNA-1/DNA-1_counts.txt replicate-1/DNA-1/DNA-1_edited_counts.txt remove remove_unmutated_codons.txt

Making a renamed version of the stats file with:
cp replicate-1/DNA-1/DNA-1_summarystats.txt replicate-1/DNA-1/DNA-1_edited_summarystats.txt

Analyzing sample: mutDNA-1

Aligning sequencing reads and making mutation counts files with the command:
dms_barcodedsubamplicons replicate-1/mutDNA-1/mutDNA-1_ LAI_env.fasta FASTQ_files/mutDNA-1-run-1_1.fastq,FASTQ_files/mutDNA-1-run-2_1.fastq,FASTQ_files/mutDNA-1-run-3_1.fastq FASTQ_files/mutDNA-1-run-1_2.fastq,FASTQ_files/mutDNA-1-run-2_2.fastq,FASTQ_files/mutDNA-1-run-3_2.fastq 90,433,39,39 421,769,38,35 765,1116,35,33 1117,1425,38,35 1424,1779,35,33 1780,2127,38,38 --R1trimlength 231 --R2trimlength 191 --barcodeinfo

Removing unmutated sites with the command:
dms_editsites replicate-1/mutDNA-1/mutDNA-1_counts.txt replicate-1/mutDNA-1/mutDNA-1_edited_counts.txt remove remove_unmutated_codons.txt

Making a renamed version of the stats file with:
cp replicate-1/mutDNA-1/mutDNA-1_summarystats.txt replicate-1/mutDNA-1/mutDNA-1_edited_summarystats.txt

Analyzing sample: virus-1-p2

Aligning sequencing reads and making mutation counts files with the command:
dms_barcodedsubamplicons replicate-1/virus-1-p2/virus-1-p2_ LAI_env.fasta FASTQ_files/virus-1-p2-run-1_1.fastq,FASTQ_files/virus-1-p2-run-2_1.fastq,FASTQ_files/virus-1-p2-run-3_1.fastq FASTQ_files/virus-1-p2-run-1_2.fastq,FASTQ_files/virus-1-p2-run-2_2.fastq,FASTQ_files/virus-1-p2-run-3_2.fastq 90,433,39,39 421,769,38,35 765,1116,35,33 1117,1425,38,35 1424,1779,35,33 1780,2127,38,38 --R1trimlength 231 --R2trimlength 191 --barcodeinfo

Removing unmutated sites with the command:
dms_editsites replicate-1/virus-1-p2/virus-1-p2_counts.txt replicate-1/virus-1-p2/virus-1-p2_edited_counts.txt remove remove_unmutated_codons.txt

Making a renamed version of the stats file with:
cp replicate-1/virus-1-p2/virus-1-p2_summarystats.txt replicate-1/virus-1-p2/virus-1-p2_edited_summarystats.txt

Analyzing sample: mutvirus-1-p2

Aligning sequencing reads and making mutation counts files with the command:
dms_barcodedsubamplicons replicate-1/mutvirus-1-p2/mutvirus-1-p2_ LAI_env.fasta FASTQ_files/mutvirus-1-p2-run-1_1.fastq,FASTQ_files/mutvirus-1-p2-run-2_1.fastq,FASTQ_files/mutvirus-1-p2-run-3_1.fastq FASTQ_files/mutvirus-1-p2-run-1_2.fastq,FASTQ_files/mutvirus-1-p2-run-2_2.fastq,FASTQ_files/mutvirus-1-p2-run-3_2.fastq 90,433,39,39 421,769,38,35 765,1116,35,33 1117,1425,38,35 1424,1779,35,33 1780,2127,38,38 --R1trimlength 231 --R2trimlength 191 --barcodeinfo

Removing unmutated sites with the command:
dms_editsites replicate-1/mutvirus-1-p2/mutvirus-1-p2_counts.txt replicate-1/mutvirus-1-p2/mutvirus-1-p2_edited_counts.txt remove remove_unmutated_codons.txt

Making a renamed version of the stats file with:
cp replicate-1/mutvirus-1-p2/mutvirus-1-p2_summarystats.txt replicate-1/mutvirus-1-p2/mutvirus-1-p2_edited_summarystats.txt

Analyzing replicate: 2

Analyzing sample: DNA-2

Aligning sequencing reads and making mutation counts files with the command:
dms_barcodedsubamplicons replicate-2/DNA-2/DNA-2_ LAI_env.fasta FASTQ_files/DNA-2-run-1_1.fastq,FASTQ_files/DNA-2-run-2_1.fastq,FASTQ_files/DNA-2-run-3_1.fastq FASTQ_files/DNA-2-run-1_2.fastq,FASTQ_files/DNA-2-run-2_2.fastq,FASTQ_files/DNA-2-run-3_2.fastq 90,433,39,39 421,769,38,35 765,1116,35,33 1117,1425,38,35 1424,1779,35,33 1780,2127,38,38 --R1trimlength 231 --R2trimlength 191 --barcodeinfo

Removing unmutated sites with the command:
dms_editsites replicate-2/DNA-2/DNA-2_counts.txt replicate-2/DNA-2/DNA-2_edited_counts.txt remove remove_unmutated_codons.txt

Making a renamed version of the stats file with:
cp replicate-2/DNA-2/DNA-2_summarystats.txt replicate-2/DNA-2/DNA-2_edited_summarystats.txt

Analyzing sample: mutDNA-2

Aligning sequencing reads and making mutation counts files with the command:
dms_barcodedsubamplicons replicate-2/mutDNA-2/mutDNA-2_ LAI_env.fasta FASTQ_files/mutDNA-2-run-1_1.fastq,FASTQ_files/mutDNA-2-run-2_1.fastq,FASTQ_files/mutDNA-2-run-3_1.fastq FASTQ_files/mutDNA-2-run-1_2.fastq,FASTQ_files/mutDNA-2-run-2_2.fastq,FASTQ_files/mutDNA-2-run-3_2.fastq 90,433,39,39 421,769,38,35 765,1116,35,33 1117,1425,38,35 1424,1779,35,33 1780,2127,38,38 --R1trimlength 231 --R2trimlength 191 --barcodeinfo

Removing unmutated sites with the command:
dms_editsites replicate-2/mutDNA-2/mutDNA-2_counts.txt replicate-2/mutDNA-2/mutDNA-2_edited_counts.txt remove remove_unmutated_codons.txt

Making a renamed version of the stats file with:
cp replicate-2/mutDNA-2/mutDNA-2_summarystats.txt replicate-2/mutDNA-2/mutDNA-2_edited_summarystats.txt

Analyzing sample: virus-2-p2

Aligning sequencing reads and making mutation counts files with the command:
dms_barcodedsubamplicons replicate-2/virus-2-p2/virus-2-p2_ LAI_env.fasta FASTQ_files/virus-2-p2-run-1_1.fastq,FASTQ_files/virus-2-p2-run-2_1.fastq,FASTQ_files/virus-2-p2-run3_1.fastq FASTQ_files/virus-2-p2-run-1_2.fastq,FASTQ_files/virus-2-p2-run-2_2.fastq,FASTQ_files/virus-2-p2-run3_2.fastq 90,433,39,39 421,769,38,35 765,1116,35,33 1117,1425,38,35 1424,1779,35,33 1780,2127,38,38 --R1trimlength 231 --R2trimlength 191 --barcodeinfo

Removing unmutated sites with the command:
dms_editsites replicate-2/virus-2-p2/virus-2-p2_counts.txt replicate-2/virus-2-p2/virus-2-p2_edited_counts.txt remove remove_unmutated_codons.txt

Making a renamed version of the stats file with:
cp replicate-2/virus-2-p2/virus-2-p2_summarystats.txt replicate-2/virus-2-p2/virus-2-p2_edited_summarystats.txt

Analyzing sample: mutvirus-2-p2

Aligning sequencing reads and making mutation counts files with the command:
dms_barcodedsubamplicons replicate-2/mutvirus-2-p2/mutvirus-2-p2_ LAI_env.fasta FASTQ_files/mutvirus-2-p2-run-1_1.fastq,FASTQ_files/mutvirus-2-p2-run-2_1.fastq,FASTQ_files/mutvirus-2-p2-run-3_1.fastq FASTQ_files/mutvirus-2-p2-run-1_2.fastq,FASTQ_files/mutvirus-2-p2-run-2_2.fastq,FASTQ_files/mutvirus-2-p2-run-3_2.fastq 90,433,39,39 421,769,38,35 765,1116,35,33 1117,1425,38,35 1424,1779,35,33 1780,2127,38,38 --R1trimlength 231 --R2trimlength 191 --barcodeinfo

Removing unmutated sites with the command:
dms_editsites replicate-2/mutvirus-2-p2/mutvirus-2-p2_counts.txt replicate-2/mutvirus-2-p2/mutvirus-2-p2_edited_counts.txt remove remove_unmutated_codons.txt

Making a renamed version of the stats file with:
cp replicate-2/mutvirus-2-p2/mutvirus-2-p2_summarystats.txt replicate-2/mutvirus-2-p2/mutvirus-2-p2_edited_summarystats.txt

Analyzing replicate: 3

Analyzing sample: DNA-3

Aligning sequencing reads and making mutation counts files with the command:
dms_barcodedsubamplicons replicate-3/DNA-3/DNA-3_ LAI_env.fasta FASTQ_files/DNA-3-run-1_1.fastq,FASTQ_files/DNA-3-run-2_1.fastq,FASTQ_files/DNA-3-run-3_1.fastq FASTQ_files/DNA-3-run-1_2.fastq,FASTQ_files/DNA-3-run-2_2.fastq,FASTQ_files/DNA-3-run-3_2.fastq 90,433,39,39 421,769,38,35 765,1116,35,33 1117,1425,38,35 1424,1779,35,33 1780,2127,38,38 --R1trimlength 231 --R2trimlength 191 --barcodeinfo

Removing unmutated sites with the command:
dms_editsites replicate-3/DNA-3/DNA-3_counts.txt replicate-3/DNA-3/DNA-3_edited_counts.txt remove remove_unmutated_codons.txt

Making a renamed version of the stats file with:
cp replicate-3/DNA-3/DNA-3_summarystats.txt replicate-3/DNA-3/DNA-3_edited_summarystats.txt

Analyzing sample: mutDNA-3

Aligning sequencing reads and making mutation counts files with the command:
dms_barcodedsubamplicons replicate-3/mutDNA-3/mutDNA-3_ LAI_env.fasta FASTQ_files/mutDNA-3-run-1_1.fastq,FASTQ_files/mutDNA-3-run-2_1.fastq,FASTQ_files/mutDNA-3-run-3_1.fastq FASTQ_files/mutDNA-3-run-1_2.fastq,FASTQ_files/mutDNA-3-run-2_2.fastq,FASTQ_files/mutDNA-3-run-3_2.fastq 90,433,39,39 421,769,38,35 765,1116,35,33 1117,1425,38,35 1424,1779,35,33 1780,2127,38,38 --R1trimlength 231 --R2trimlength 191 --barcodeinfo

Removing unmutated sites with the command:
dms_editsites replicate-3/mutDNA-3/mutDNA-3_counts.txt replicate-3/mutDNA-3/mutDNA-3_edited_counts.txt remove remove_unmutated_codons.txt

Making a renamed version of the stats file with:
cp replicate-3/mutDNA-3/mutDNA-3_summarystats.txt replicate-3/mutDNA-3/mutDNA-3_edited_summarystats.txt

Analyzing sample: virus-3-p1

Aligning sequencing reads and making mutation counts files with the command:
dms_barcodedsubamplicons replicate-3/virus-3-p1/virus-3-p1_ LAI_env.fasta FASTQ_files/virus-3-p1-run-1_1.fastq,FASTQ_files/virus-3-p1-run-2_1.fastq,FASTQ_files/virus-3-p1-run-3_1.fastq FASTQ_files/virus-3-p1-run-1_2.fastq,FASTQ_files/virus-3-p1-run-2_2.fastq,FASTQ_files/virus-3-p1-run-3_2.fastq 90,433,39,39 421,769,38,35 765,1116,35,33 1117,1425,38,35 1424,1779,35,33 1780,2127,38,38 --R1trimlength 231 --R2trimlength 191 --barcodeinfo

Removing unmutated sites with the command:
dms_editsites replicate-3/virus-3-p1/virus-3-p1_counts.txt replicate-3/virus-3-p1/virus-3-p1_edited_counts.txt remove remove_unmutated_codons.txt

Making a renamed version of the stats file with:
cp replicate-3/virus-3-p1/virus-3-p1_summarystats.txt replicate-3/virus-3-p1/virus-3-p1_edited_summarystats.txt

Analyzing sample: mutvirus-3-p1

Aligning sequencing reads and making mutation counts files with the command:
dms_barcodedsubamplicons replicate-3/mutvirus-3-p1/mutvirus-3-p1_ LAI_env.fasta FASTQ_files/mutvirus-3-p1-run-1_1.fastq,FASTQ_files/mutvirus-3-p1-run-2_1.fastq,FASTQ_files/mutvirus-3-p1-run-3_1.fastq FASTQ_files/mutvirus-3-p1-run-1_2.fastq,FASTQ_files/mutvirus-3-p1-run-2_2.fastq,FASTQ_files/mutvirus-3-p1-run-3_2.fastq 90,433,39,39 421,769,38,35 765,1116,35,33 1117,1425,38,35 1424,1779,35,33 1780,2127,38,38 --R1trimlength 231 --R2trimlength 191 --barcodeinfo

Removing unmutated sites with the command:
dms_editsites replicate-3/mutvirus-3-p1/mutvirus-3-p1_counts.txt replicate-3/mutvirus-3-p1/mutvirus-3-p1_edited_counts.txt remove remove_unmutated_codons.txt

Making a renamed version of the stats file with:
cp replicate-3/mutvirus-3-p1/mutvirus-3-p1_summarystats.txt replicate-3/mutvirus-3-p1/mutvirus-3-p1_edited_summarystats.txt

Analyzing sample: virus-3-p2

Aligning sequencing reads and making mutation counts files with the command:
dms_barcodedsubamplicons replicate-3/virus-3-p2/virus-3-p2_ LAI_env.fasta FASTQ_files/virus-3-p2-run-1_1.fastq,FASTQ_files/virus-3-p2-run-2_1.fastq,FASTQ_files/virus-3-p2-run-3_1.fastq FASTQ_files/virus-3-p2-run-1_2.fastq,FASTQ_files/virus-3-p2-run-2_2.fastq,FASTQ_files/virus-3-p2-run-3_2.fastq 90,433,39,39 421,769,38,35 765,1116,35,33 1117,1425,38,35 1424,1779,35,33 1780,2127,38,38 --R1trimlength 231 --R2trimlength 191 --barcodeinfo

Removing unmutated sites with the command:
dms_editsites replicate-3/virus-3-p2/virus-3-p2_counts.txt replicate-3/virus-3-p2/virus-3-p2_edited_counts.txt remove remove_unmutated_codons.txt

Making a renamed version of the stats file with:
cp replicate-3/virus-3-p2/virus-3-p2_summarystats.txt replicate-3/virus-3-p2/virus-3-p2_edited_summarystats.txt

Analyzing sample: mutvirus-3-p2

Aligning sequencing reads and making mutation counts files with the command:
dms_barcodedsubamplicons replicate-3/mutvirus-3-p2/mutvirus-3-p2_ LAI_env.fasta FASTQ_files/mutvirus-3-p2-run-1_1.fastq,FASTQ_files/mutvirus-3-p2-run-2_1.fastq,FASTQ_files/mutvirus-3-p2-run-3_1.fastq FASTQ_files/mutvirus-3-p2-run-1_2.fastq,FASTQ_files/mutvirus-3-p2-run-2_2.fastq,FASTQ_files/mutvirus-3-p2-run-3_2.fastq 90,433,39,39 421,769,38,35 765,1116,35,33 1117,1425,38,35 1424,1779,35,33 1780,2127,38,38 --R1trimlength 231 --R2trimlength 191 --barcodeinfo

Removing unmutated sites with the command:
dms_editsites replicate-3/mutvirus-3-p2/mutvirus-3-p2_counts.txt replicate-3/mutvirus-3-p2/mutvirus-3-p2_edited_counts.txt remove remove_unmutated_codons.txt

Making a renamed version of the stats file with:
cp replicate-3/mutvirus-3-p2/mutvirus-3-p2_summarystats.txt replicate-3/mutvirus-3-p2/mutvirus-3-p2_edited_summarystats.txt

Analyzing replicate: 3b

Analyzing sample: DNA-3b

Aligning sequencing reads and making mutation counts files with the command:
dms_barcodedsubamplicons replicate-3b/DNA-3b/DNA-3b_ LAI_env.fasta FASTQ_files/DNA-3b-run-1_1.fastq,FASTQ_files/DNA-3b-run-2_1.fastq FASTQ_files/DNA-3b-run-1_2.fastq,FASTQ_files/DNA-3b-run-2_2.fastq 90,433,39,39 421,769,38,35 765,1116,35,33 1117,1425,38,35 1424,1779,35,33 1780,2127,38,38 --R1trimlength 231 --R2trimlength 191 --barcodeinfo

Removing unmutated sites with the command:
dms_editsites replicate-3b/DNA-3b/DNA-3b_counts.txt replicate-3b/DNA-3b/DNA-3b_edited_counts.txt remove remove_unmutated_codons.txt

Making a renamed version of the stats file with:
cp replicate-3b/DNA-3b/DNA-3b_summarystats.txt replicate-3b/DNA-3b/DNA-3b_edited_summarystats.txt

Analyzing sample: mutDNA-3b

Aligning sequencing reads and making mutation counts files with the command:
dms_barcodedsubamplicons replicate-3b/mutDNA-3b/mutDNA-3b_ LAI_env.fasta FASTQ_files/mutDNA-3b-run-1_1.fastq,FASTQ_files/mutDNA-3b-run-2_1.fastq FASTQ_files/mutDNA-3b-run-1_2.fastq,FASTQ_files/mutDNA-3b-run-2_2.fastq 90,433,39,39 421,769,38,35 765,1116,35,33 1117,1425,38,35 1424,1779,35,33 1780,2127,38,38 --R1trimlength 231 --R2trimlength 191 --barcodeinfo

Removing unmutated sites with the command:
dms_editsites replicate-3b/mutDNA-3b/mutDNA-3b_counts.txt replicate-3b/mutDNA-3b/mutDNA-3b_edited_counts.txt remove remove_unmutated_codons.txt

Making a renamed version of the stats file with:
cp replicate-3b/mutDNA-3b/mutDNA-3b_summarystats.txt replicate-3b/mutDNA-3b/mutDNA-3b_edited_summarystats.txt

Analyzing sample: virus-3b-1-p2

Aligning sequencing reads and making mutation counts files with the command:
dms_barcodedsubamplicons replicate-3b/virus-3b-1-p2/virus-3b-1-p2_ LAI_env.fasta FASTQ_files/virus-3b-1-p2-run-1_1.fastq,FASTQ_files/virus-3b-1-p2-run-2_1.fastq FASTQ_files/virus-3b-1-p2-run-1_2.fastq,FASTQ_files/virus-3b-1-p2-run-2_2.fastq 90,433,39,39 421,769,38,35 765,1116,35,33 1117,1425,38,35 1424,1779,35,33 1780,2127,38,38 --R1trimlength 231 --R2trimlength 191 --barcodeinfo

Removing unmutated sites with the command:
dms_editsites replicate-3b/virus-3b-1-p2/virus-3b-1-p2_counts.txt replicate-3b/virus-3b-1-p2/virus-3b-1-p2_edited_counts.txt remove remove_unmutated_codons.txt

Making a renamed version of the stats file with:
cp replicate-3b/virus-3b-1-p2/virus-3b-1-p2_summarystats.txt replicate-3b/virus-3b-1-p2/virus-3b-1-p2_edited_summarystats.txt

Analyzing sample: mutvirus-3b-1-p2

Aligning sequencing reads and making mutation counts files with the command:
dms_barcodedsubamplicons replicate-3b/mutvirus-3b-1-p2/mutvirus-3b-1-p2_ LAI_env.fasta FASTQ_files/mutvirus-3b-1-p2-run-1_1.fastq,FASTQ_files/mutvirus-3b-1-p2-run-2_1.fastq FASTQ_files/mutvirus-3b-1-p2-run-1_2.fastq,FASTQ_files/mutvirus-3b-1-p2-run-2_2.fastq 90,433,39,39 421,769,38,35 765,1116,35,33 1117,1425,38,35 1424,1779,35,33 1780,2127,38,38 --R1trimlength 231 --R2trimlength 191 --barcodeinfo

Removing unmutated sites with the command:
dms_editsites replicate-3b/mutvirus-3b-1-p2/mutvirus-3b-1-p2_counts.txt replicate-3b/mutvirus-3b-1-p2/mutvirus-3b-1-p2_edited_counts.txt remove remove_unmutated_codons.txt

Making a renamed version of the stats file with:
cp replicate-3b/mutvirus-3b-1-p2/mutvirus-3b-1-p2_summarystats.txt replicate-3b/mutvirus-3b-1-p2/mutvirus-3b-1-p2_edited_summarystats.txt

Analyzing sample: virus-3b-2-p2

Aligning sequencing reads and making mutation counts files with the command:
dms_barcodedsubamplicons replicate-3b/virus-3b-2-p2/virus-3b-2-p2_ LAI_env.fasta FASTQ_files/virus-3b-2-p2-run-1_1.fastq,FASTQ_files/virus-3b-2-p2-run-2_1.fastq FASTQ_files/virus-3b-2-p2-run-1_2.fastq,FASTQ_files/virus-3b-2-p2-run-2_2.fastq 90,433,39,39 421,769,38,35 765,1116,35,33 1117,1425,38,35 1424,1779,35,33 1780,2127,38,38 --R1trimlength 231 --R2trimlength 191 --barcodeinfo

Removing unmutated sites with the command:
dms_editsites replicate-3b/virus-3b-2-p2/virus-3b-2-p2_counts.txt replicate-3b/virus-3b-2-p2/virus-3b-2-p2_edited_counts.txt remove remove_unmutated_codons.txt

Making a renamed version of the stats file with:
cp replicate-3b/virus-3b-2-p2/virus-3b-2-p2_summarystats.txt replicate-3b/virus-3b-2-p2/virus-3b-2-p2_edited_summarystats.txt

Analyzing sample: mutvirus-3b-2-p2

Aligning sequencing reads and making mutation counts files with the command:
dms_barcodedsubamplicons replicate-3b/mutvirus-3b-2-p2/mutvirus-3b-2-p2_ LAI_env.fasta FASTQ_files/mutvirus-3b-2-p2-run-1_1.fastq,FASTQ_files/mutvirus-3b-2-p2-run-2_1.fastq FASTQ_files/mutvirus-3b-2-p2-run-1_2.fastq,FASTQ_files/mutvirus-3b-2-p2-run-2_2.fastq 90,433,39,39 421,769,38,35 765,1116,35,33 1117,1425,38,35 1424,1779,35,33 1780,2127,38,38 --R1trimlength 231 --R2trimlength 191 --barcodeinfo

Removing unmutated sites with the command:
dms_editsites replicate-3b/mutvirus-3b-2-p2/mutvirus-3b-2-p2_counts.txt replicate-3b/mutvirus-3b-2-p2/mutvirus-3b-2-p2_edited_counts.txt remove remove_unmutated_codons.txt

Making a renamed version of the stats file with:
cp replicate-3b/mutvirus-3b-2-p2/mutvirus-3b-2-p2_summarystats.txt replicate-3b/mutvirus-3b-2-p2/mutvirus-3b-2-p2_edited_summarystats.txt
```

## Make plots summarizing the alignments¶

Next, I use `dms_summarizealignments` to make summary plots of the alignments.

In [8]:

```
print "\nThe results of the alignment will be summarized using:"
!dms_summarizealignments -v

for replicate in replicates:
    
    print "\nAnalyzing replicate: %s" %replicate
    
    # Make the alignment summaries
    outprefix = 'replicate-%s/alignmentsummary_'%replicate    
    cmd_summarizealignments = ' '.join([
            'dms_summarizealignments',
            outprefix,
            'barcodedsubamplicons',
            ' '.join(alignprefixes_names[replicate]),
            '--writemutfreqs'])
    print ("Making alignment summary plots with the command:\n" + cmd_summarizealignments)
    log = !$cmd_summarizealignments
    
    for suffix in ['reads.pdf', 'barcodes.pdf', 'depth.pdf', 'mutdepth.pdf', 'mutfreqs.pdf', 'mutcounts_all.pdf', 'mutcounts_multi_nt.pdf']:
        pdf = outprefix + suffix
        png = os.path.splitext(pdf)[0] + '.png'
        !convert -density 192 -trim $pdf $png
        print("\nHere is %s" % png)
        display(Image(png, width=500))
```

```
The results of the alignment will be summarized using:
dms_summarizealignments 1.1.dev16

Analyzing replicate: 1
Making alignment summary plots with the command:
dms_summarizealignments replicate-1/alignmentsummary_ barcodedsubamplicons replicate-1/DNA-1/DNA-1_edited_,DNA-1 replicate-1/mutDNA-1/mutDNA-1_edited_,mutDNA-1 replicate-1/virus-1-p2/virus-1-p2_edited_,virus-1-p2 replicate-1/mutvirus-1-p2/mutvirus-1-p2_edited_,mutvirus-1-p2 --writemutfreqs

Here is replicate-1/alignmentsummary_reads.png
```

```
Here is replicate-1/alignmentsummary_barcodes.png
```

```
Here is replicate-1/alignmentsummary_depth.png
```

```
Here is replicate-1/alignmentsummary_mutdepth.png
```

```
Here is replicate-1/alignmentsummary_mutfreqs.png
```

```
Here is replicate-1/alignmentsummary_mutcounts_all.png
```

```
Here is replicate-1/alignmentsummary_mutcounts_multi_nt.png
```

```
Analyzing replicate: 2
Making alignment summary plots with the command:
dms_summarizealignments replicate-2/alignmentsummary_ barcodedsubamplicons replicate-2/DNA-2/DNA-2_edited_,DNA-2 replicate-2/mutDNA-2/mutDNA-2_edited_,mutDNA-2 replicate-2/virus-2-p2/virus-2-p2_edited_,virus-2-p2 replicate-2/mutvirus-2-p2/mutvirus-2-p2_edited_,mutvirus-2-p2 --writemutfreqs

Here is replicate-2/alignmentsummary_reads.png
```

```
Here is replicate-2/alignmentsummary_barcodes.png
```

```
Here is replicate-2/alignmentsummary_depth.png
```

```
Here is replicate-2/alignmentsummary_mutdepth.png
```

```
Here is replicate-2/alignmentsummary_mutfreqs.png
```

```
Here is replicate-2/alignmentsummary_mutcounts_all.png
```

```
Here is replicate-2/alignmentsummary_mutcounts_multi_nt.png
```

```
Analyzing replicate: 3
Making alignment summary plots with the command:
dms_summarizealignments replicate-3/alignmentsummary_ barcodedsubamplicons replicate-3/DNA-3/DNA-3_edited_,DNA-3 replicate-3/mutDNA-3/mutDNA-3_edited_,mutDNA-3 replicate-3/virus-3-p1/virus-3-p1_edited_,virus-3-p1 replicate-3/mutvirus-3-p1/mutvirus-3-p1_edited_,mutvirus-3-p1 replicate-3/virus-3-p2/virus-3-p2_edited_,virus-3-p2 replicate-3/mutvirus-3-p2/mutvirus-3-p2_edited_,mutvirus-3-p2 --writemutfreqs

Here is replicate-3/alignmentsummary_reads.png
```

```
Here is replicate-3/alignmentsummary_barcodes.png
```

```
Here is replicate-3/alignmentsummary_depth.png
```

```
Here is replicate-3/alignmentsummary_mutdepth.png
```

```
Here is replicate-3/alignmentsummary_mutfreqs.png
```

```
Here is replicate-3/alignmentsummary_mutcounts_all.png
```

```
Here is replicate-3/alignmentsummary_mutcounts_multi_nt.png
```

```
Analyzing replicate: 3b
Making alignment summary plots with the command:
dms_summarizealignments replicate-3b/alignmentsummary_ barcodedsubamplicons replicate-3b/DNA-3b/DNA-3b_edited_,DNA-3b replicate-3b/mutDNA-3b/mutDNA-3b_edited_,mutDNA-3b replicate-3b/virus-3b-1-p2/virus-3b-1-p2_edited_,virus-3b-1-p2 replicate-3b/mutvirus-3b-1-p2/mutvirus-3b-1-p2_edited_,mutvirus-3b-1-p2 replicate-3b/virus-3b-2-p2/virus-3b-2-p2_edited_,virus-3b-2-p2 replicate-3b/mutvirus-3b-2-p2/mutvirus-3b-2-p2_edited_,mutvirus-3b-2-p2 --writemutfreqs

Here is replicate-3b/alignmentsummary_reads.png
```

```
Here is replicate-3b/alignmentsummary_barcodes.png
```

```
Here is replicate-3b/alignmentsummary_depth.png
```

```
Here is replicate-3b/alignmentsummary_mutdepth.png
```

```
Here is replicate-3b/alignmentsummary_mutfreqs.png
```

```
Here is replicate-3b/alignmentsummary_mutcounts_all.png
```

```
Here is replicate-3b/alignmentsummary_mutcounts_multi_nt.png
```

I will also use `dms_summarizealignments` to generate a *mutfreqs.txt* plot for all samples from all replicates:

In [9]:

```
# Same thing as above, but for all samples from all replicates
all_alignprefixes_names = []
for replicate in replicates:
    all_alignprefixes_names.extend(alignprefixes_names[replicate])

print "\nThe results of the alignment will be summarized using:"
!dms_summarizealignments -v
    
# Make the alignment summaries
outprefix = 'alignmentsummary_'
cmd_summarizealignments = ' '.join([
        'dms_summarizealignments',
        outprefix,
        'barcodedsubamplicons',
        ' '.join(all_alignprefixes_names),
        '--writemutfreqs'])
print ("Making alignment summary plots with the command:\n" + cmd_summarizealignments)
log = !$cmd_summarizealignments

suffix = 'mutfreqs.pdf'
pdf = outprefix + suffix
png = os.path.splitext(pdf)[0] + '.png'
!convert -density 192 -trim $pdf $png
print("\nHere is %s" % png)
display(Image(png, width=500))
```

```
The results of the alignment will be summarized using:
dms_summarizealignments 1.1.dev16
Making alignment summary plots with the command:
dms_summarizealignments alignmentsummary_ barcodedsubamplicons replicate-1/DNA-1/DNA-1_edited_,DNA-1 replicate-1/mutDNA-1/mutDNA-1_edited_,mutDNA-1 replicate-1/virus-1-p2/virus-1-p2_edited_,virus-1-p2 replicate-1/mutvirus-1-p2/mutvirus-1-p2_edited_,mutvirus-1-p2 replicate-2/DNA-2/DNA-2_edited_,DNA-2 replicate-2/mutDNA-2/mutDNA-2_edited_,mutDNA-2 replicate-2/virus-2-p2/virus-2-p2_edited_,virus-2-p2 replicate-2/mutvirus-2-p2/mutvirus-2-p2_edited_,mutvirus-2-p2 replicate-3/DNA-3/DNA-3_edited_,DNA-3 replicate-3/mutDNA-3/mutDNA-3_edited_,mutDNA-3 replicate-3/virus-3-p1/virus-3-p1_edited_,virus-3-p1 replicate-3/mutvirus-3-p1/mutvirus-3-p1_edited_,mutvirus-3-p1 replicate-3/virus-3-p2/virus-3-p2_edited_,virus-3-p2 replicate-3/mutvirus-3-p2/mutvirus-3-p2_edited_,mutvirus-3-p2 replicate-3b/DNA-3b/DNA-3b_edited_,DNA-3b replicate-3b/mutDNA-3b/mutDNA-3b_edited_,mutDNA-3b replicate-3b/virus-3b-1-p2/virus-3b-1-p2_edited_,virus-3b-1-p2 replicate-3b/mutvirus-3b-1-p2/mutvirus-3b-1-p2_edited_,mutvirus-3b-1-p2 replicate-3b/virus-3b-2-p2/virus-3b-2-p2_edited_,virus-3b-2-p2 replicate-3b/mutvirus-3b-2-p2/mutvirus-3b-2-p2_edited_,mutvirus-3b-2-p2 --writemutfreqs

Here is alignmentsummary_mutfreqs.png
```

## Merge codon counts between replicates and plot the results of the merged data¶

First, I will merge codon counts between replicates without normalizing the counts beforehand.

In [10]:

```
print "Codon counts will be merged using:"
!dms_merge -v

combined_counts_dir_prefix = 'combined_counts'
# combined_samples = ['DNA', 'mutDNA', 'virus-p2', 'mutvirus-p2']
combined_samples = ['DNA', 'virus-p2', 'mutDNA', 'mutvirus-p2']
for combined_sample in combined_samples:
    if not os.path.isdir('%s/%s/'%(combined_counts_dir_prefix, combined_sample)):
        os.makedirs('%s/%s/'%(combined_counts_dir_prefix, combined_sample))
    
# First, I will sum the counts files for 3b-1 and 3b-2
# ... for the mutant virus
combined_counts_file_mutvirus_p2_3b1_3b2 = '%s/mutvirus-p2/mutvirus-3b-p2_edited_counts.txt'%combined_counts_dir_prefix
cmd_merge = ' '.join([
                'dms_merge',
                combined_counts_file_mutvirus_p2_3b1_3b2, # outfile
                'sum',
                ' '.join([edited_counts_files['3b'][sample] for sample in ['mutvirus-3b-1-p2', 'mutvirus-3b-2-p2']])
                ])
print ("\nMerging counts files with the command:\n" + cmd_merge)
log = !$cmd_merge

# ... and for the wild-type virus
combined_counts_file_virus_p2_3b1_3b2 = '%s/virus-p2/virus-3b-p2_edited_counts.txt'%combined_counts_dir_prefix
cmd_merge = ' '.join([
                'dms_merge',
                combined_counts_file_virus_p2_3b1_3b2, # outfile
                'sum',
                ' '.join([edited_counts_files['3b'][sample] for sample in ['virus-3b-1-p2', 'virus-3b-2-p2']])
                ])
print ("\nMerging counts files with the command:\n" + cmd_merge)
log = !$cmd_merge

# Next, I will sum the counts files for all replicates. This time I will normalize the counts files before
# summing them together. For the virus-p2 and mutvirus-p2 samples for the 3b replicates, I will use the
# counts files from above that are the sum of replicates 3b-1 and 3b-2.

# ... for the DNA samples
combined_sample = 'DNA'
combined_counts_file_DNA = '%s/%s/%s_edited_counts.txt'%(combined_counts_dir_prefix, combined_sample, combined_sample)
cmd_merge = ' '.join([
                'dms_merge',
                combined_counts_file_DNA, # outfile
                'sum',
                ' '.join([edited_counts_files[replicate]['DNA-%s'%replicate] for replicate in replicates]),
                '--normalize'
                ])
print ("\nMerging counts files with the command:\n" + cmd_merge)
log = !$cmd_merge

# ... for the mutDNA samples
combined_sample = 'mutDNA'
combined_counts_file_DNA = '%s/%s/%s_edited_counts.txt'%(combined_counts_dir_prefix, combined_sample, combined_sample)
cmd_merge = ' '.join([
                'dms_merge',
                combined_counts_file_DNA, # outfile
                'sum',
                ' '.join([edited_counts_files[replicate]['mutDNA-%s'%replicate] for replicate in replicates]),
                '--normalize'
                ])
print ("\nMerging counts files with the command:\n" + cmd_merge)
log = !$cmd_merge

# ... for the virus-p2 samples
combined_sample = 'virus-p2'
combined_counts_file_DNA = '%s/%s/%s_edited_counts.txt'%(combined_counts_dir_prefix, combined_sample, combined_sample)
cmd_merge = ' '.join([
                'dms_merge',
                combined_counts_file_DNA, # outfile
                'sum',
                ' '.join([edited_counts_files[replicate]['virus-%s-p2'%replicate] for replicate in [1, 2, 3]] + [combined_counts_file_virus_p2_3b1_3b2]),
                '--normalize'
                ])
print ("\nMerging counts files with the command:\n" + cmd_merge)
log = !$cmd_merge

# ... for the mutvirus-p2 samples
combined_sample = 'mutvirus-p2'
combined_counts_file_DNA = '%s/%s/%s_edited_counts.txt'%(combined_counts_dir_prefix, combined_sample, combined_sample)
cmd_merge = ' '.join([
                'dms_merge',
                combined_counts_file_DNA, # outfile
                'sum',
                ' '.join([edited_counts_files[replicate]['mutvirus-%s-p2'%replicate] for replicate in [1, 2, 3]] + [combined_counts_file_mutvirus_p2_3b1_3b2]),
                '--normalize'
                ])
print ("\nMerging counts files with the command:\n" + cmd_merge)
log = !$cmd_merge
```

```
Codon counts will be merged using:
dms_merge 1.1.dev16

Merging counts files with the command:
dms_merge combined_counts/mutvirus-p2/mutvirus-3b-p2_edited_counts.txt sum replicate-3b/mutvirus-3b-1-p2/mutvirus-3b-1-p2_edited_counts.txt replicate-3b/mutvirus-3b-2-p2/mutvirus-3b-2-p2_edited_counts.txt

Merging counts files with the command:
dms_merge combined_counts/virus-p2/virus-3b-p2_edited_counts.txt sum replicate-3b/virus-3b-1-p2/virus-3b-1-p2_edited_counts.txt replicate-3b/virus-3b-2-p2/virus-3b-2-p2_edited_counts.txt

Merging counts files with the command:
dms_merge combined_counts/DNA/DNA_edited_counts.txt sum replicate-1/DNA-1/DNA-1_edited_counts.txt replicate-2/DNA-2/DNA-2_edited_counts.txt replicate-3/DNA-3/DNA-3_edited_counts.txt replicate-3b/DNA-3b/DNA-3b_edited_counts.txt --normalize

Merging counts files with the command:
dms_merge combined_counts/mutDNA/mutDNA_edited_counts.txt sum replicate-1/mutDNA-1/mutDNA-1_edited_counts.txt replicate-2/mutDNA-2/mutDNA-2_edited_counts.txt replicate-3/mutDNA-3/mutDNA-3_edited_counts.txt replicate-3b/mutDNA-3b/mutDNA-3b_edited_counts.txt --normalize

Merging counts files with the command:
dms_merge combined_counts/virus-p2/virus-p2_edited_counts.txt sum replicate-1/virus-1-p2/virus-1-p2_edited_counts.txt replicate-2/virus-2-p2/virus-2-p2_edited_counts.txt replicate-3/virus-3-p2/virus-3-p2_edited_counts.txt combined_counts/virus-p2/virus-3b-p2_edited_counts.txt --normalize

Merging counts files with the command:
dms_merge combined_counts/mutvirus-p2/mutvirus-p2_edited_counts.txt sum replicate-1/mutvirus-1-p2/mutvirus-1-p2_edited_counts.txt replicate-2/mutvirus-2-p2/mutvirus-2-p2_edited_counts.txt replicate-3/mutvirus-3-p2/mutvirus-3-p2_edited_counts.txt combined_counts/mutvirus-p2/mutvirus-3b-p2_edited_counts.txt --normalize
```

Next, I will use `dms_summarizealignments` to summarize the merged counts files. To do so, I must first create dummy *summarystats.txt* files:

In [11]:

```
# Make a new statsfile with the same suffix as the corresponding edited counts file
replicate = 1
for (combined_sample, sample) in zip(combined_samples, samples[replicate]):
    # Specify the name of the dummy statsfile
    dummy_statsfile = '%s/%s/%s_edited_summarystats.txt'%(combined_counts_dir_prefix, combined_sample, combined_sample)
    
    # Specify the name of the statsfile that will be used to make the dummy statsfile
    alignmentdir = 'replicate-%s/%s/' %(replicate, sample)
    alignprefix = '%s%s_' %(alignmentdir, sample)
    edited_alignprefix = '%s%s_edited_' %(alignmentdir, sample)
    edited_statsfile = '%ssummarystats.txt' %edited_alignprefix # same as statsfile, but with the edited_alignprefix extension that matches the edited_countsfile, which is required for alignmentsummaries to work properly
    
    # Make the dummy stats file
    cmd_cp = 'cp %s %s'%(edited_statsfile, dummy_statsfile)
    print ("\nMaking a dummy stats file with the command:\n" + cmd_cp)
    !$cmd_cp
```

```
Making a dummy stats file with the command:
cp replicate-1/DNA-1/DNA-1_edited_summarystats.txt combined_counts/DNA/DNA_edited_summarystats.txt

Making a dummy stats file with the command:
cp replicate-1/mutDNA-1/mutDNA-1_edited_summarystats.txt combined_counts/virus-p2/virus-p2_edited_summarystats.txt

Making a dummy stats file with the command:
cp replicate-1/virus-1-p2/virus-1-p2_edited_summarystats.txt combined_counts/mutDNA/mutDNA_edited_summarystats.txt

Making a dummy stats file with the command:
cp replicate-1/mutvirus-1-p2/mutvirus-1-p2_edited_summarystats.txt combined_counts/mutvirus-p2/mutvirus-p2_edited_summarystats.txt
```

Next, I will generate plots summarizing the merged counts:

In [12]:

```
print "\nThe results of the alignment will be summarized using:"
!dms_summarizealignments -v

# Specify the input and output prefixes
alignprefixes_names = []
for combined_sample in combined_samples:
    alignprefix = '%s/%s/%s_edited_' %(combined_counts_dir_prefix, combined_sample, combined_sample)
    alignprefixes_names.append("%s,%s"%(alignprefix, combined_sample))
outprefix = '%s/alignmentsummary_'%combined_counts_dir_prefix

# Run dms_summarizealignments
cmd_summarizealignments = ' '.join([
        'dms_summarizealignments',
        outprefix,
        'barcodedsubamplicons',
        ' '.join(alignprefixes_names),
        '--writemutfreqs'])
print ("Making alignment summary plots with the command:\n" + cmd_summarizealignments)
log = !$cmd_summarizealignments

# Display some of the output files
for suffix in ['mutdepth.pdf', 'mutfreqs.pdf', 'mutcounts_all.pdf', 'mutcounts_multi_nt.pdf']:
    pdf = outprefix + suffix
    png = os.path.splitext(pdf)[0] + '.png'
    !convert -density 192 -trim $pdf $png
    print("\nHere is %s" % png)
    display(Image(png, width=500))

# Remove output files generated by the dummy statsfiles
for suffix in ['reads.pdf', 'barcodes.pdf', 'depth.pdf']:
    rm_cmd = 'rm %s%s'%(outprefix, suffix)
    print ("Removing file with the command:\n" + rm_cmd)
    !$rm_cmd
```

```
The results of the alignment will be summarized using:
dms_summarizealignments 1.1.dev16
Making alignment summary plots with the command:
dms_summarizealignments combined_counts/alignmentsummary_ barcodedsubamplicons combined_counts/DNA/DNA_edited_,DNA combined_counts/virus-p2/virus-p2_edited_,virus-p2 combined_counts/mutDNA/mutDNA_edited_,mutDNA combined_counts/mutvirus-p2/mutvirus-p2_edited_,mutvirus-p2 --writemutfreqs

Here is combined_counts/alignmentsummary_mutdepth.png
```

```
Here is combined_counts/alignmentsummary_mutfreqs.png
```

```
Here is combined_counts/alignmentsummary_mutcounts_all.png
```

```
Here is combined_counts/alignmentsummary_mutcounts_multi_nt.png
```

```
Removing file with the command:
rm combined_counts/alignmentsummary_reads.pdf
Removing file with the command:
rm combined_counts/alignmentsummary_barcodes.pdf
Removing file with the command:
rm combined_counts/alignmentsummary_depth.pdf
```

In [ ]:

```

```
