# Supplementary material for: Experimental Estimation of the Effects of All Amino-Acid Mutations to HIV’s Envelope Protein on Viral Replication in Cell Culture
Source: PLoS Pathog. 2016 Dec 13;12(12):e1006114. doi: 10.1371/journal.ppat.1006114 (PMC5189966; doi:10.1371/journal.ppat.1006114)
Supplement: S3 File — (ZIP) [file ppat.1006114.s013.zip › S3_File_notebooks/analyze_site_specific_mutation_frequencies/AnalyzeSiteSpecificMutationFrequencies.html]

AnalyzeSiteSpecificMutationFrequencies


# Analyzing site-specific mutation frequencies¶

The analyses I carry out in this notebook include:

- Testing how efficiently the library sampled all possible codon and amino-acid mutations
- Comparing the purging of synonymous and nonsynonymous multi-nucleotide codon changes across *env*'s primary sequence.
- Binning sites based on whether the change in "error-corrected" per-codon mutation frequency at that site increased by more than three fold, suggesting these sites are adapting to cell culture.
- Measuring the overlap between replicates in sites of putative cell-culture adaptation, plotting the results as a Venn diagram.
- Examining the change in site-averaged per-codon "error-corrected" mutation frequency for all sites and for subsets of sites: sites of putative cell-culture adaptation vs. other sites.

Hugh Haddox, January-12-2016

In [1]:

```
import sys
import os
sys.path.append('../scripts/')
import prefsutils
import matplotlib
from matplotlib.ticker import ScalarFormatter
matplotlib.use('pdf')
matplotlib.rc('text', usetex=True)
import pylab
from matplotlib_venn import venn3
import random
import doctest
from IPython.display import Image, display
import math
```

## Compute site-specific mutation frequencies¶

I will compute site-specific mutation frequencies for each replicate mutant library and wild-type control before and after selection. To do so, I will first read in mutation counts from a `_counts.txt` file for each replicate as generated in the IPython notebook: `../AlignSequencingReadsAndComputeMutationalCounts.ipynb`. I will then compute site-specific mutation frequencies (the frequency of all mutant codons) based on raw mutation counts. I will also compute the site-specific frequencies of just multi-nucleotide codon mutations, which are not expected to be influenced very much by errors from PCR and deep sequencing, as well as de-novo mutations from viral replication.

In [2]:

```
# Specify sample names and initiate lists and dictionaries
replicates = [1, 2, 3, '3b-1', '3b-2']
samples = {}
samples[1] = ['DNA-1', 'mutDNA-1', 'virus-1-p2', 'mutvirus-1-p2']
samples[2] = ['DNA-2', 'mutDNA-2', 'virus-2-p2', 'mutvirus-2-p2']
samples[3] = ['DNA-3', 'mutDNA-3', 'virus-3-p1', 'mutvirus-3-p1', 'virus-3-p2', 'mutvirus-3-p2']
samples['3b-1'] = ['DNA-3b', 'mutDNA-3b', 'virus-3b-1-p2', 'mutvirus-3b-1-p2']
samples['3b-2'] = ['DNA-3b', 'mutDNA-3b', 'virus-3b-2-p2', 'mutvirus-3b-2-p2']
samples['combined'] = ['DNA', 'mutDNA', 'virus-p2', 'mutvirus-p2']
countsfiles = {}
counts = {}
mutfreqs = {}
multi_nt_mutfreqs = {}

for rep in samples:
    # Initiation of sub-lists and sub-dictionaries
    countsfiles[rep] = {}
    counts[rep] = {}
    mutfreqs[rep] = {}
    multi_nt_mutfreqs[rep] = {}
    
    # Account for fact that the "super" replicate 3b has "sub" replicates 3b-1 and 3b-2, while the other
    # replicates do not have sub replicates
    if rep in ['3b-1', '3b-2']:
        super_rep = '3b'
    else:
        super_rep = rep
    
    # I will specify the counts files
    for sample in samples[rep]:
        if rep == 'combined':
            counts_file_prefix = '../combined_counts/%s/%s' %(sample, sample)
            countsfiles[rep][sample] = '%s_edited_counts.txt'%counts_file_prefix
        else:
            counts_file_prefix = '../replicate-%s/%s/%s' %(super_rep, sample, sample)
            countsfiles[rep][sample] = '%s_edited_counts.txt'%counts_file_prefix
    
    # Next, I will compute site-specific mutation frequencies
    for sample in countsfiles[rep]:
        counts[rep][sample] = prefsutils.ConvertCodonCountsFileIntoDictionary(countsfiles[rep][sample])
        mutfreqs[rep][sample] = prefsutils.ComputeSiteSpecificMutationFrequencies(counts[rep][sample])        
        multi_nt_mutfreqs[rep][sample] = prefsutils.ComputeSiteSpecificMutationFrequencies(counts[rep][sample], MultiNT=True)
```

I will also make a version of the dictionaries specifying mutation frequencies (*mutfreqs* and *multi\_nt\_mutfreqs*) in HXB2 numbering, leaving out the sites in LAI that aren't in HXB2 (i.e. 143-147 in LAI).

In [3]:

```
mutfreqs_hxb2 = {}
multi_nt_mutfreqs_hxb2 = {}
for rep in mutfreqs:
    mutfreqs_hxb2[rep] = {}
    multi_nt_mutfreqs_hxb2[rep] = {}
    for sample in mutfreqs[rep]:
        mutfreqs_hxb2[rep][sample] = {}
        multi_nt_mutfreqs_hxb2[rep][sample] = {}
        for site in mutfreqs[rep][sample]:
            if int(site) in [143, 144, 145, 146, 147]:
                continue
            hxb2_site = prefsutils.ConvertNumberingLAItoHXBII(site)
            mutfreqs_hxb2[rep][sample][hxb2_site] = mutfreqs[rep][sample][site]
            multi_nt_mutfreqs_hxb2[rep][sample][hxb2_site] = multi_nt_mutfreqs[rep][sample][site]
```

Next, I will make a dictionary of "error-corrected" site-specific mutation frequencies, where the mutation frequencies of mutant samples are subtracted by the mutation frequencies in the corresponding wild-type controls. This dictionary will only contain mutDNA and mutvirus-p2 samples.

In [4]:

```
corr_mutfreqs_hxb2 = {}

# Compute "error-corrected" mutation frequencies after two passages
for rep in mutfreqs_hxb2:
    
    # Get sample names and initiate dictionary
    DNA_sample = samples[rep][0]
    mutDNA_sample = samples[rep][1]
    virus_p2_sample = samples[rep][-2]
    mutvirus_p2_sample = samples[rep][-1]
    corr_mutfreqs_hxb2[rep] = {}
    corr_mutfreqs_hxb2[rep][mutDNA_sample] = {}
    corr_mutfreqs_hxb2[rep][mutvirus_p2_sample] = {}
    
    # Compute error-corrected mutation frequencies for each site for the overall, syn., and nonsyn. mutation rates
    sites = mutfreqs_hxb2[rep][samples[rep][0]].keys()
    for site in sites:
        corr_mutfreqs_hxb2[rep][mutDNA_sample][site] = {}
        corr_mutfreqs_hxb2[rep][mutvirus_p2_sample][site] = {}
        for mut_type in ['overall', 'N', 'S']:
            DNAfreq = mutfreqs_hxb2[rep][DNA_sample][site][mut_type]
            mutDNAfreq = mutfreqs_hxb2[rep][mutDNA_sample][site][mut_type]
            virus_p2_freq = mutfreqs_hxb2[rep][virus_p2_sample][site][mut_type]
            mutvirus_p2_freq = mutfreqs_hxb2[rep][mutvirus_p2_sample][site][mut_type]
            
            # Add data to dictionary, watching out for sites without any possible synonymous mutations
            if mutvirus_p2_freq == 'na': # i.e. there aren't any possible mutations at this site
                corr_mutfreqs_hxb2[rep][mutDNA_sample][site][mut_type] = 'na'
                corr_mutfreqs_hxb2[rep][mutvirus_p2_sample][site][mut_type] = 'na'
            else:
                corr_mutfreqs_hxb2[rep][mutDNA_sample][site][mut_type] = mutDNAfreq - DNAfreq
                corr_mutfreqs_hxb2[rep][mutvirus_p2_sample][site][mut_type] = mutvirus_p2_freq - virus_p2_freq
```

## Test how efficiently the individual replicate libraries and the combined replicate libraries sample all possible mutations¶

The question I wish to answer is: what percentage of the possible amino-acid mutations were sampled at least X times in the starting mutant DNA libraries before functional selection? First, I will make a dictionary of amino-acid counts using the codon-counts dictionary from above for just the mutant libraries before selection.

In [5]:

```
# Make a dictionary of amino-acid counts from the dictionary of codon counts above
aa_counts = {}
for rep in counts:
    aa_counts[rep]={}
    samples_to_analyze = [samples[rep][1]] # the mutDNA sample
    for sample in samples_to_analyze:
        aa_counts[rep][sample]={}
        for site in counts[rep][sample]:
            aa_counts[rep][sample][site]={}
            for codon in counts[rep][sample][site]:
                if codon in ['WT', 'total_counts']:
                    continue
                aa = prefsutils.TranslateCodon(codon)
                if aa in aa_counts[rep][sample][site]:
                    aa_counts[rep][sample][site][aa] += counts[rep][sample][site][codon]
                else:
                    aa_counts[rep][sample][site][aa] = counts[rep][sample][site][codon]
            assert len(aa_counts[rep][sample][site]) == 21
            aa_counts[rep][sample][site]['WT'] = prefsutils.TranslateCodon(counts[rep][sample][site]['WT'])
```

Next, I will determine what percentage of both codon and amino-acid mutations are observed greater than X number of times in each replicate and in all replicates combined. X is a cutoff specified by the variable *counts\_cutoff*.

In [6]:

```
# Bin mutations based on whether their counts are greater than or equal to a given threshold
counts_cutoff = 3

# Do this for different counts dictionaries, one for codons and one for amino acids
counts_dicts = [(counts, 'codon counts'), (aa_counts, 'amino-acid counts')]
for (counts_dict, label) in counts_dicts:
    print "\n############################"
    print "Analyzing sampling of %s"%label
    print "############################"
    for rep in counts_dict:
        print "\n... for replicate: %s"%rep
        samples_to_analyze = [samples[rep][1]] # the mutDNA sample
        for sample in samples_to_analyze:
            total_mutations_analyzed = 0
            mutations_observed_gtoet_counts_cutoff = 0
            sites = counts_dict[rep][sample].keys()
            for site in sites:
                
                # Make a list of mutations to consider, leaving out stops and the wild-type character
                if label in ['codon counts']: # i.e. codon counts
                    mut_chars = [char for char in counts_dict[rep][sample][site] if char not in ['WT', 'total_counts', 'TAA', 'TAG', 'TGA', counts_dict[rep][sample][site]['WT']]]
                    assert len(mut_chars) == 60 # number of mutant characters
                if label in ['amino-acid counts']: # i.e. amino-acid counts
                    mut_chars = [char for char in counts_dict[rep][sample][site] if char not in ['WT', 'total_counts', '*', counts_dict[rep][sample][site]['WT']]]
                    assert len(mut_chars) == 19 # number of mutant characters
                
                # Bin mutations in above list based on counts
                for char in mut_chars:                    
                    if counts_dict[rep][sample][site][char] >= counts_cutoff:
                        mutations_observed_gtoet_counts_cutoff += 1
                        total_mutations_analyzed += 1
                    else:
                        total_mutations_analyzed += 1
            
            # Compute percent of sites greater than or equal to cutoff and print the results
            if label == 'codon counts':
                assert len(sites) * 60 == total_mutations_analyzed
            if label == 'amino-acid counts':
                assert len(sites) * 19 == total_mutations_analyzed
            print "total possible mutations = %s"%total_mutations_analyzed
            print "number of mutations observed >= %s times = %s"%(counts_cutoff, mutations_observed_gtoet_counts_cutoff)
            print "fraction of mutations observed >= %s times = %s"%(counts_cutoff, float(mutations_observed_gtoet_counts_cutoff)/float(total_mutations_analyzed))
```

```
############################
Analyzing sampling of codon counts
############################

... for replicate: 1
total possible mutations = 40620
number of mutations observed >= 3 times = 20382
fraction of mutations observed >= 3 times = 0.501772525849

... for replicate: 2
total possible mutations = 40620
number of mutations observed >= 3 times = 23238
fraction of mutations observed >= 3 times = 0.572082717873

... for replicate: 3
total possible mutations = 40620
number of mutations observed >= 3 times = 18612
fraction of mutations observed >= 3 times = 0.458197932053

... for replicate: 3b-1
total possible mutations = 40620
number of mutations observed >= 3 times = 19592
fraction of mutations observed >= 3 times = 0.482323978336

... for replicate: 3b-2
total possible mutations = 40620
number of mutations observed >= 3 times = 19592
fraction of mutations observed >= 3 times = 0.482323978336

... for replicate: combined
total possible mutations = 40620
number of mutations observed >= 3 times = 36531
fraction of mutations observed >= 3 times = 0.899335302806

############################
Analyzing sampling of amino-acid counts
############################

... for replicate: 1
total possible mutations = 12863
number of mutations observed >= 3 times = 10566
fraction of mutations observed >= 3 times = 0.821425794916

... for replicate: 2
total possible mutations = 12863
number of mutations observed >= 3 times = 11174
fraction of mutations observed >= 3 times = 0.868693150898

... for replicate: 3
total possible mutations = 12863
number of mutations observed >= 3 times = 10215
fraction of mutations observed >= 3 times = 0.794138225919

... for replicate: 3b-1
total possible mutations = 12863
number of mutations observed >= 3 times = 10395
fraction of mutations observed >= 3 times = 0.808131851046

... for replicate: 3b-2
total possible mutations = 12863
number of mutations observed >= 3 times = 10395
fraction of mutations observed >= 3 times = 0.808131851046

... for replicate: combined
total possible mutations = 12863
number of mutations observed >= 3 times = 12571
fraction of mutations observed >= 3 times = 0.977299230351
```

## Compare the purging of synonymous and non-synonymous mutaitons for sites within vs. outside of the Rev-response element (RRE)¶

I will test whether synonymous selection is higher in the RRE using the following steps:

1) For each site within a sliding window of a given size, I will compute F\_N as the fraction of reads reporting a nonsynonymous multi-nucleotide codon mutation in the mutvirus-p2 library. I will then sum F\_N values for all sites in the sliding window. Next, I will compute F\_N values for the mutDNA library and sum these values across sites in the window. Then, to determine the fold-change in average F\_N upon selection, I will divide the F\_N sum for the mutvirus-p2 library by the F\_N sum for the mutDNA library (mutvirus-p2:mutDNA).

```
* Note: the above algorithm is the default setting for the below function *PlotSiteFreqs*. To change the way this statistic is computed to dividing the site-specific F_N value in the mutvirus-p2 library by the corresponding value in the mutDNA library before summing across sites, then set the variable *avg_freqs_before_dividing* to be False.
```

2) Similarly, I will repeat the above step for synonymous mutations (F\_S instead of F\_N) at those sites that have synonymous multi-nucleotide mutations (many won't).

3) Plot the distribution of these statistics as a function of primary sequence.

Note, the location of the RRE in LAI numbering is:

- codons within the RRE: 500-618 (LAI numbering)
- codons not within the RRE: 31-500 and 619-707 (LAI numbering)

In [7]:

```
def PlotSiteFreqs(mut_type, plotfile, plot_type='raw', plot_ratio=False, consider_all_sites=False, ylim_max=None, window_size=19, avg_freqs_before_dividing=True):
    """
    This function plots the frequencies of site-specific multi-nucleotide codon changes for either synonymous
    or nonsynonymous mutations.
    
    *mut_type* : a string specifying whether to plot data for synonymous mutations ('S'), nonsynonymous mutations
    ('N'), or both ('both')
    
    *plotfile* : the name of the output file (string ending in '.pdf')
    
    *plot_type* : a string specifying one of the below options:
        'raw' : plots the raw data without any smoothing. The x-axis is the codon number in full-length env.
        
        'smooth_and_remove_na_sites' : plots data after smoothing it by plotting the average statistic of all
        sites in a window of length *window_size* centered on the position in question. The x-axis is the consecutive
        numbering of all codons that have a particular statistic assigned to them. Not all sites will have
        non-synonymous multi-nucleotide codon mutations (they have 'na' as their value instead). These sites are
        removed and the remaining sites are numbered consecutively.
    
    *plot_ratio* : a boolean specifying whether to plot mutDNA and mutvirus-p2 seperately or whether to plot the
    ratio of mutvirus-p2:mutDNA
        
    *window_size* : an odd integer specifying the window size for smoothing the data. The smoothing will involve
    taking the average across the window centered on a point, and assigning the average to that central point.
        
    """
    # Specify the boundaries of the RRE in LAI numbering
    RRE_boundaries = (495, 613) # (500, 618)
    
    # Initiate figure
    pylab.figure(figsize=(15,5))
    ax = pylab.axes()

    # Compile a list of sample names and samples for plotting
    mutDNA_sample = [sample for sample in samples[rep] if 'mutDNA' in sample][0]
    mutvirus_sample = [sample for sample in samples[rep] if 'mutvirus' in sample and 'p2' in sample][0] # Note: If there are multiple mutvirus-p2 samples, I will only make the plot using the first one in the list
    ratio_samples = 'mutvirus-p2:mutDNA'
    if plot_ratio:
        samples_to_plot = [ratio_samples]
    else:
        samples_to_plot = [mutDNA_sample, mutvirus_sample]
        
    # Compile a list of sites for plotting and a list of sites with usable data for averaging over sliding windows
    sites_to_plot = {}
    sites_with_usable_data = {}
    if consider_all_sites: # make a plot with all sites in Env
        for mut in ['N', 'S']:
            sites_to_plot[mut] = [int(site) for site in multi_nt_mutfreqs_hxb2[rep][mutDNA_sample]]
            sites_to_plot[mut].sort() # must order sites for plotting
            if plot_ratio: # discount sites without data for *mut* and sites for which the mutDNA frequency = 0, making taking a mutvirus:mutDNA ratio impossible
                sites_with_usable_data[mut] = [site for site in sites_to_plot[mut] if multi_nt_mutfreqs_hxb2[rep][mutDNA_sample][str(site)][mut] != 'na' and multi_nt_mutfreqs_hxb2[rep][mutDNA_sample][str(site)][mut] > 0]
            else: # only discount sites without data for *mut*
                sites_with_usable_data[mut] = [site for site in sites_to_plot[mut] if multi_nt_mutfreqs_hxb2[rep][mutDNA_sample][str(site)][mut] != 'na']
    else: # only plot sites with usable data. The x-axis will no longer be codon number in Env; instead, it will be codons in primary sequence indexed starting at 1, 2, 3, ..., n_usable_sites
        for mut in ['N', 'S']:
            if mut_type in ['S', 'both']: # only plot sites with usable data for synonymous multi-nt changes
                relevant_mut = 'S'
            else:
                relevant_mut = 'N'
            if plot_ratio: # discount sites without data for *mut* and sites for which the mutDNA frequency = 0, making taking a mutvirus:mutDNA ratio impossible
                sites_to_plot[mut] = [int(site) for site in multi_nt_mutfreqs_hxb2[rep][mutDNA_sample] if multi_nt_mutfreqs_hxb2[rep][mutDNA_sample][str(site)][relevant_mut] != 'na' and multi_nt_mutfreqs_hxb2[rep][mutDNA_sample][str(site)][relevant_mut] > 0]
            else: # only discount sites without data for *mut*
                sites_to_plot[mut] = [int(site) for site in multi_nt_mutfreqs_hxb2[rep][mutDNA_sample] if multi_nt_mutfreqs_hxb2[rep][mutDNA_sample][str(site)][relevant_mut] != 'na']
            sites_to_plot[mut].sort() # must order sites for plotting
            sites_with_usable_data[mut] = sites_to_plot[mut]
        
    # Compute data for plotting and make the plot
    Fs = {}
    maxF = 0
    minF = 1e6 # arbitrarily high number
    if mut_type == 'both':
        mut_type = ['N', 'S']
    for mut in mut_type:

        # Compile mutation frequencies
        print "mut = %s"%mut
        Fs[mut] = {}
        for sample in [mutDNA_sample, mutvirus_sample]:
            Fs[mut][sample] = dict((site, multi_nt_mutfreqs_hxb2[rep][sample][str(site)][mut]) for site in sites_with_usable_data[mut])

        # Compute fractions if plotting fractions
        if plot_ratio:
            assert len(Fs[mut][mutvirus_sample]) == len(Fs[mut][mutDNA_sample])
            Fs[mut][ratio_samples] = dict((site, Fs[mut][mutvirus_sample][site]/Fs[mut][mutDNA_sample][site]) for site in sites_with_usable_data[mut])

        # Plot the mutation frequencies as a function of primary sequences
        for sample in samples_to_plot:
            # Plot the data
            if plot_type == 'raw':
                xvalues = sites_to_plot[mut]
                yvalues = [Fs[mut][sample][site] for site in xvalues]
                pylab.plot(xvalues, yvalues, label = "F_%s_%s"%(mut, sample), linewidth = 2)
                pylab.plot((RRE_boundaries[0], RRE_boundaries[0]), (0, 1), 'k--', linewidth = 1)
                pylab.plot((RRE_boundaries[1], RRE_boundaries[1]), (0, 1), 'k--', linewidth = 1)
                xlabel = 'codon position'
                pylab.xlim([31, 707])
                if max(Fs[mut][sample].values()) > maxF:
                    maxF = max(Fs[mut][sample].values())
                if min(Fs[mut][sample].values()) < minF:
                    minF = min(Fs[mut][sample].values())

            elif plot_type == 'smooth_and_remove_na_sites':

                # Smooth the data
                assert window_size%2.0 > 0.0, "window_size must be an odd integer"
                
                # Define the points in each sliding window
                first_center_point = sites_to_plot[mut][(window_size-1)/2]
                last_center_point = sites_to_plot[mut][-1-(window_size-1)/2]
                avg_Fs_in_window = {}
                lengths_Fs_in_window = []
                for center_i in range(len(sites_to_plot[mut])):
                    center_point = sites_to_plot[mut][center_i]
                    if first_center_point <= center_point <= last_center_point:
                        sites_in_window = [site for site in sites_to_plot[mut][center_i-(window_size-1)/2 : center_i+1+(window_size-1)/2] if site in sites_with_usable_data[mut]]
                        if avg_freqs_before_dividing:
                            mutvirus_p2_values = [Fs[mut][mutvirus_sample][site] for site in sites_in_window]
                            mutDNA_values = [Fs[mut][mutDNA_sample][site] for site in sites_in_window] 
                            avg_mutvirus_p2_values = sum(mutvirus_p2_values)/len(mutvirus_p2_values)
                            avg_mutDNA_values = sum(mutDNA_values)/len(mutDNA_values)
                            avg_Fs_in_window[center_point] = avg_mutvirus_p2_values/avg_mutDNA_values
                            lengths_Fs_in_window.append(len(mutDNA_values))
                        if not avg_freqs_before_dividing:
                            Fs_in_window = [Fs[mut][sample][site] for site in sites_in_window if site in Fs[mut][sample]] # excludes sites for which there is no usable data since this dictionary lacks those sites
                            lengths_Fs_in_window.append(len(Fs_in_window))
                            assert len(Fs_in_window) > 0, "There are no sites with usable data in the window centered on site: %s"%(center_point)
                            avg_Fs_in_window[center_point] = (sum(Fs_in_window)/float(len(Fs_in_window)))
                        if avg_Fs_in_window[center_point] > maxF:
                            maxF = avg_Fs_in_window[center_point]
                        if avg_Fs_in_window[center_point] < minF:
                            minF = avg_Fs_in_window[center_point]
                
                print "Average number of datapoints per sliding window = %s"%(sum(lengths_Fs_in_window)/len(lengths_Fs_in_window))
                
                # Determine which sites are within the RRE to draw a window
                sites_before_RRE = [site for site in sites_to_plot[mut] if site < RRE_boundaries[0]]
                sites_after_RRE = [site for site in sites_to_plot[mut] if site > RRE_boundaries[1]]

                # Plot the data
                if mut_type == 'N' or consider_all_sites: # use the numbering provided in *sites_to_plot[mut]*
                    xvalues = [site for site in avg_Fs_in_window]
                    xvalues.sort()
                    yvalues = [avg_Fs_in_window[site] for site in xvalues]
                    xlabel = 'codon position'
                    pylab.xlim([min(xvalues), max(xvalues)])
                    RRE_boundaries_plot = RRE_boundaries
                else: # number xvalues consecutively starting at 1, 2, 3, ...
                    ordered_sites = [site for site in avg_Fs_in_window]
                    ordered_sites.sort()
                    enumerated_ordered_sites = list(enumerate(ordered_sites, start=1))
                    xvalues = [x for (x, site) in enumerated_ordered_sites]
                    yvalues = [avg_Fs_in_window[site] for (x, site) in enumerated_ordered_sites]
                    start_RRE_x = None
                    end_RRE_x = None
                    for (x, site) in enumerated_ordered_sites:
                        if site >= RRE_boundaries[0]:
                            if not start_RRE_x:
                                start_RRE_x = x
                                print "start_RRE_x = %s" %start_RRE_x
                                print "site = %s" %site
                        if site > RRE_boundaries[1]:
                            if not end_RRE_x:
                                end_RRE_x = x-1
                                print "end_RRE_x = %s" %end_RRE_x
                                print "site = %s" %site
                    RRE_boundaries_plot = (start_RRE_x, end_RRE_x)
                    xlabel = 'codons ordered in primary sequence'
                    pylab.xlim([min(xvalues), max(xvalues)])
                if plot_ratio == True:
                    if mut == 'N':
                        label = 'nonsynonymous'
                    if mut == 'S':
                        label = 'synonymous'
                else:
                    label = "F_%s_%s"%(mut, sample)
                pylab.plot(xvalues, yvalues, label=label, linewidth = 3)

        # Plot vertical lines showing the boundaries of the RRE
        if plot_ratio:
            pylab.plot((RRE_boundaries_plot[0], RRE_boundaries_plot[0]), (0, 10), 'k--', linewidth = 1)
            pylab.plot((RRE_boundaries_plot[1], RRE_boundaries_plot[1]), (0, 10), 'k--', linewidth = 1)
            pylab.axvspan(RRE_boundaries_plot[0], RRE_boundaries_plot[1], color='0.75', alpha=0.25)
            pylab.plot((min(xvalues), max(xvalues)), (1, 1), 'k--', linewidth = 1)
        else:
            pylab.plot((RRE_boundaries_plot[0], RRE_boundaries_plot[0]), (0, 1), 'k--', linewidth = 1)
            pylab.plot((RRE_boundaries_plot[1], RRE_boundaries_plot[1]), (0, 1), 'k--', linewidth = 1)

    # Additional plot parameters
    pylab.legend(loc = 3, fontsize = 25) #bbox_to_anchor=(1.4, 1.0),
    if ylim_max:
        pylab.ylim([0, ylim_max])
    else:
        if plot_ratio: # Set ylim and log scale if plotting ratios
            ylabel = 'multi-nucleotide mutation\nfrequency fold change'
            base = 2
            ax.set_yscale('log', basey=base)
            pylab.ylim([minF*0.9, maxF*1.1])
            for axis in [ax.xaxis, ax.yaxis]:
                axis.set_major_formatter(ScalarFormatter())
                pylab.yticks([0.125, 0.25, 0.5, 1, 2])
        else:
            ylabel = 'mutation frequency'
            if plot_type == 'smooth_and_remove_na_sites':
                pylab.ylim([0, max(avg_Fs_in_window)+0.1*max(avg_Fs_in_window)])
            else:
                pylab.ylim([0, maxF+0.1*maxF])
    
    ax.set_ylabel(ylabel, fontsize=25)
    ax.set_xlabel(xlabel, fontsize=25)
    ax.yaxis.labelpad = 15
    ax.xaxis.labelpad = 15
    pylab.yticks(fontsize=25)
    pylab.xticks(fontsize=25)
    pylab.rcParams['xtick.major.pad']='10'
    pylab.rcParams['ytick.major.pad']='10'
    ax.xaxis.set_tick_params(width=2.0, length = 5)
    ax.yaxis.set_tick_params(width=2.0, length = 5)
    [i.set_linewidth(2.0) for i in ax.spines.itervalues()]

    # Display the plot
    pylab.savefig(plotfile, bbox_inches = 'tight')
    pylab.close()
    pdf = plotfile
    png = os.path.splitext(pdf)[0] + '.png'
    !convert -density 192 -trim $pdf $png
    print("\nHere is %s" % png)
    display(Image(png, width=900))
```

This cell specifies plotting parameters such as:

- *rep* : the name of the replicate to plot
- *window\_size* : the size of the sliding window (integer)

In [8]:

```
# Analysis parameters
rep = 'combined'
mut_type = 'both' # can be: 'both', 'S', 'N'
window_size = 51
plot_ratio = True
consider_all_sites = True
plotfile = 'multi_nt_%s_mut_freq_primary_seq_rep_%s.pdf'%(mut_type, rep)
plot_type = 'smooth_and_remove_na_sites' # 'raw'
ylim_max = None
PlotSiteFreqs(mut_type, plotfile, plot_type, plot_ratio=plot_ratio, consider_all_sites=consider_all_sites, ylim_max=ylim_max, window_size = window_size)
```

```
mut = N
Average number of datapoints per sliding window = 51
mut = S
Average number of datapoints per sliding window = 9

Here is multi_nt_both_mut_freq_primary_seq_rep_combined.png
```

## Find sites of putative cell-culture adaptation where the mutation frequency (or "error-corrected" mutation frequency) underwent a large increase upon selection¶

The average per-codon mutation frequency decreases only slightly upon selection, despite the expectation that many mutations should be deleterious and purged from the library. Since stop codons are effeciently purged from the library, one explanation for the slight decrease in overall mutation frequency is that there are some sites where the mutation frequency is increasing upon selection, possibly due to cell-culture adaptation. I will test this hypothesis by binning sites based on whether or not their chage in mutation frequency (mutvirus-p2:mutDNA) increases by more than some cutoff (currently, the cutoff [see *frac\_cutoff* from below] is a 3-fold increase). To account for errors from PCR and deep sequencing, as well as *de novo* mutations from viral replication, I will conduct this analysis with "error-corrected" mutation frequencies, computed by subtracting mutation frequencies from mutant libraries by the mutation frequencies in their respective wild-type controls.

First, I will compute site-specific ratios of the mutation frequency after:before selection (mutvirus:mutDNA). I will also compute the ratio after correcting for mutation frequencies observed in the wild-type controls([mutvirus-virus]/[mutDNA-DNA]). Next, I will bin sites based on whether the ratio is above or below some specified threshold *frac\_cutoff*.

In [9]:

```
# Specify the cutoff for binning sites by their fractional increase or decrease upon selection
frac_cutoff = 3.0

# Initiate dictionaries
mutratios = {} # mutratios[rep][site] = mutvirus_freq / mutDNA_freq
corrmutratios = {} # corrmutratios[rep][site] = (mutvirus_freq - virus_freq) / (mutDNA_freq - DNA_freq)
binned_sites = {}
site_bins = ['mutfreqs_increased', 'mutfreqs_decreased', 'corr_mutfreqs_increased', 'corr_mutfreqs_decreased']

# Compute ratios and "corrected" ratios after two passages
for rep in replicates:
    mutratios[rep] = {}
    corrmutratios[rep] = {}
    binned_sites[rep] = dict((site_bin, []) for site_bin in site_bins)
    
    # Get a list of sites from one of the samples
    sites = mutfreqs[rep][samples[rep][0]].keys()
    
    # Compute site-specific ratios after two passages
    for site in sites:
        DNAfreq = mutfreqs[rep][samples[rep][0]][site]['overall']
        mutDNAfreq = mutfreqs[rep][samples[rep][1]][site]['overall']
        virusfreq = mutfreqs[rep][samples[rep][-2]][site]['overall']
        mutvirusfreq = mutfreqs[rep][samples[rep][-1]][site]['overall']
        
        # Compute ratio of mutvirus:mutDNA mutation frequencies
        mutratios[rep][site] = mutvirusfreq / mutDNAfreq
        if mutratios[rep][site] < frac_cutoff:
            binned_sites[rep]['mutfreqs_decreased'].append(site)
        elif mutratios[rep][site] > frac_cutoff:
            binned_sites[rep]['mutfreqs_increased'].append(site)
        else:
            print "This site had a ratio of 1.0: %s"%site
            
        # Compute "corrected" ratios
        corrmutratios[rep][site] = (mutvirusfreq - virusfreq) / (mutDNAfreq - DNAfreq)
        if corrmutratios[rep][site] < frac_cutoff:
            binned_sites[rep]['corr_mutfreqs_decreased'].append(site)
        elif corrmutratios[rep][site] > frac_cutoff:
            binned_sites[rep]['corr_mutfreqs_increased'].append(site)
        else:
            print "This site had a ratio of %s: %s"%(frac_cutoff, site)
    
    # Make sure there isn't an intersection between mutually exclusive lists
    assert 677 == len(binned_sites[rep]['mutfreqs_decreased']) + len(binned_sites[rep]['mutfreqs_increased'])
    assert 677 == len(binned_sites[rep]['corr_mutfreqs_decreased']) + len(binned_sites[rep]['corr_mutfreqs_increased'])
    for site in binned_sites[rep]['mutfreqs_decreased']:
        assert site not in binned_sites[rep]['mutfreqs_increased']
    for site in binned_sites[rep]['mutfreqs_increased']:
        assert site not in binned_sites[rep]['mutfreqs_decreased']
    for site in binned_sites[rep]['corr_mutfreqs_decreased']:
        assert site not in binned_sites[rep]['corr_mutfreqs_increased']
    for site in binned_sites[rep]['corr_mutfreqs_increased']:
        assert site not in binned_sites[rep]['corr_mutfreqs_decreased']
```

## Import data on site-specific amino-acid preferences and relative solvent accessibility for analyzing sites of putative cell-culture adaptation¶

Read in site-specific amino-acid preferences averaged between replicates (hxb2 numbering) inferred using the IPython notebook `../InferPreferences.ipynb`. Then rescale the preferences with a stringency parameter that maximized the correlation between the averaged preferences and natural amino-acid frequencies. Then, remove stop codons and renormalize the remaining preferences to one, before computing a hydropathy score for each site based on the preferences. Also determine the hydropathy of the wild-type amino acid at each site.

In [10]:

```
# preferences input file
prefsfile = '../averaged_preferences/avg_prefs_p2_nhxb2.txt'
CIs_present = False

# read in the preferences (hxb2 numbering)
prefs = prefsutils.ConvertPrefsFileToDictionary(prefsfile, CIs_present=CIs_present)

# rescale the preferences with a stringency parameter that optimizes the correlation with natural sequences
beta = 2.1
rescaled_prefs = prefsutils.RescalePreferences(prefs, beta)

# remove stop codons and normalize the remaining preferences to one
renormalized_prefs = prefsutils.NormalizePrefsMinusStop(rescaled_prefs)

# compute the hydropathy of the preferences
renormalized_prefs_hydropathy = prefsutils.CalculateAAPreferenceHydropathy(renormalized_prefs)

# determine the hydropathy of the WT amino acid at each site
prefs_wt_1 = {}
for site in prefs:
    prefs_wt_1[site] = {}
    for char in prefs[site]:
        if char == prefs[site]['WT']:
            prefs_wt_1[site][char] = 1.0
        else:
            prefs_wt_1[site][char] = 0.0
WT_aa_hydropathy = prefsutils.CalculateAAPreferenceHydropathy(prefs_wt_1)
entropies = [rescaled_prefs[site]['entropy'] for site in rescaled_prefs]
```

Read in site-specific RSA values computed from the PDB structure 4TVP in the IPython notebook `../secondary_structure_and_RSA_analysis/secondary_structure_and_RSA_analysis.ipynb`:

In [11]:

```
RSAfile = open('../secondary_structure_and_RSA_analysis/RSA_4tvp_renamed_chains_rmTERandHETATM_rm321A.txt', 'r')
RSAs = {}
for line in RSAfile:
    if line[0] == '#':
        continue
    (site, RSA) = line.strip().split()
    RSAs[site] = RSA

# add null entries for sites that lack RSA values
for i in range(31, 708):
    i = str(i)
    if i not in RSAs:
        RSAs[i] = 'nd'
```

## Make venn diagrams showing the overlap of sites of putative cell-culture adaptation between replicates¶

Here, I will measure the overlap between replicates in sites where the error-corrected per-codon mutation frequency increased upon selection by more than a specified cutoff (currently >3-fold).

First, I will plot the overlap between replicates 1, 2, and 3 as a Venn diagram. Next, I will plot the expected overlap if the sites in each replicate were randomly drawn from *env*'s primary sequence and test whether there is a significant difference between the random "null" overlap and the observed overlap.

In addition to making the Venn diagrams, I will also print to the terminal data on putative sites of cell-culture adaptation, including wild-type residue, the site-specific entropy of the preferences, and the site-speicifc hydropathy of both the wild-type residue and the preferences. I will do so for sites that overlap between all three replicates as well as a separate analysis of sites that overlap between at least two of the replicates.

In [12]:

```
def RandomizationSignificanceForTripartiteOverlap(n1, n2, n3, totn, nrand, obs_overlap):
    """
    This function uses randomization to compute the probabiliy of an overalp equal to or
    greater than *obs_overlap* for three groups of sites given the null hypothesis that
    the three groups of sites were drawn at random from *totn* sites (without replacement).
    
    Input:
    
        *n1*, *n2*, and *n3* : the number of sites in groups 1, 2, and 3

        *totn* : the total number of sites that n1, n2, and n3 were "drawn" from

        *nrand* : the number of randomization tests

        *obs_overlap* : the observed number of overlapping sites betwee the three groups

    Output:
    
        *P* : the probability that an overlap equal to or greater than *obs_overlap*
        would have occured by random drawing of sites without replacement.
    """
    # Make a list of sites that will be sampled from
    sites = [i for i in range(totn)]
    # List that keeps track of 
    n_tripartite_overlap = []
    
    # Randomization tests
    for n in range(nrand):
        
        # Randomly select integers with the range 1 to totn, inclusive
        s1 = random.sample(sites, n1)
        s2 = random.sample(sites, n2)
        s3 = random.sample(sites, n3)
        assert len(s1) == n1
        assert len(s2) == n2
        assert len(s3) == n3
        tots = s1 + s2 + s3
        
        # Determine number of sites that are present in all three groups
        o123 = []
        for s in tots:
            if s in s1 and s in s2 and s in s3:
                if s not in o123:
                    o123.append(s)
        n_tripartite_overlap.append(len(o123))

    # print sum(n_tripartite_overlap)/float(len(n_tripartite_overlap))
    # print n_tripartite_overlap
    
    n_less_than_obs_overlap = [n for n in n_tripartite_overlap if n < obs_overlap]
    
    if len(n_less_than_obs_overlap) == len(n_tripartite_overlap):
        P = 'P $<$ %.4f'%(1.0/len(n_tripartite_overlap))
    else:
        P = 'P = %.3f'%(float(len(n_less_than_obs_overlap))/len(n_tripartite_overlap))
    
    return P
    
doctest.testmod()
```

Out[12]:

```
TestResults(failed=0, attempted=0)
```

In [13]:

```
def ComputeExpectedOverlapThreeSamples(totn, n1, n2, n3):
    """
    This function is for computing the expected overlap between three samples given:
    
    Input:    
        *totn* : the total number of sites each sample is drawn from

        *n1*, *n2*, *n3* : the number of sites in each sample
        
    Output:
        *expected_dist* : a list giving the expected number of counts in each category.
        Categories are named by a letter "o" followed by one to three numbers. A single number
        indicates a category where a site was only found in the specified replicate. Two numbers
        give the intersection between the two specified replicates to the exclusion of the third.
        Three numbers give the intersection between all three replicates.
    
    Code for doctest:
    >>> (totn, n1, n2, n3) = (677, 47, 52, 68)
    >>> expected_dist = ComputeExpectedOverlapThreeSamples(totn, n1, n2, n3)
    >>> expected_dist[-1]
    0.4
    """
    totn = float(totn)
    
    o123 = totn * n1/totn * n2/totn * n3/totn
    o12 = (totn * n1/totn * n2/totn) - o123
    o13 = (totn * n1/totn * n3/totn) - o123
    o23 = (totn * n2/totn * n3/totn) - o123
    o1 = n1 - o12 - o13 - o123
    o2 = n2 - o12 - o23 - o123
    o3 = n3 - o13 - o23 - o123
    
    expected_dist = [o1, o2, o12, o3, o13, o23, o123]
    expected_dist = [round(i, 1) for i in expected_dist]
    
    assert round(n1, 5) == round(sum([o1, o12, o13, o123]), 5)
    assert round(n2, 5) == round(sum([o2, o12, o23, o123]), 5)
    assert round(n3, 5) == round(sum([o3, o13, o23, o123]), 5)
    
    return (expected_dist)

doctest.testmod()
```

Out[13]:

```
TestResults(failed=0, attempted=3)
```

In [14]:

```
def PlotThreePartVennDiagram(repA, repB, repC, plotfile):
    """
    This function makes a venn diagram showing the overlap between a given three replicates. The overlap is in
    terms of the identity of sites binned in the category 'corr_mutfreqs_increased' (see above), which is where a
    site's "error-corrected" mutation frequency increases upon selection.
    """
    sites1 = binned_sites[repA]['corr_mutfreqs_increased']
    sites2 = binned_sites[repB]['corr_mutfreqs_increased']
    sites3 = binned_sites[repC]['corr_mutfreqs_increased']
    totn = 677
    n1 = len(sites1)
    n2 = len(sites2)
    n3 = len(sites3)

    # Determine the intersection between all three groups and for pairwise groups
    o123 = []
    o12 = []
    o13 = []
    o23 = []
    tots = sites1 + sites2 + sites3
    for s in tots:
        if s in sites1 and s in sites2 and s in sites3:
            if s not in o123:
                o123.append(s)
        elif s in sites1 and s in sites2:
            if s not in o12:
                o12.append(s)
        elif s in sites1 and s in sites3:
            if s not in o13:
                o13.append(s)
        elif s in sites2 and s in sites3:
            if s not in o23:
                o23.append(s)
    obs_overlap = len(o123)
    
    # Sort sites
    o123 = [int(site) for site in o123]
    o123.sort()
    o123 = [str(site) for site in o123]
    
    # Print sites (hxb2 numbering) and their corrected mutant ratios to the terminal
    o123_hxb2 = [prefsutils.ConvertNumberingLAItoHXBII('%s'%site) for site in o123]
    o12_hxb2 = [prefsutils.ConvertNumberingLAItoHXBII('%s'%site) for site in o12]
    o13_hxb2 = [prefsutils.ConvertNumberingLAItoHXBII('%s'%site) for site in o13]
    o23_hxb2 = [prefsutils.ConvertNumberingLAItoHXBII('%s'%site) for site in o23]
    
    # ...for sites that overlapped between at least two replicates
    o_at_least_two_reps = o123 + o12 + o13 + o23
    o_at_least_two_reps = [int(site) for site in o_at_least_two_reps]
    o_at_least_two_reps.sort()
    o_at_least_two_reps_hxb2 = o123_hxb2 + o12_hxb2 + o13_hxb2 + o23_hxb2
    o_at_least_two_reps_hxb2 = [int(site) for site in o_at_least_two_reps_hxb2]
    o_at_least_two_reps_hxb2.sort()
    o_at_least_two_reps_hxb2 = [str(site) for site in o_at_least_two_reps_hxb2]
    print "\nThe number of sites that overlapped between at least two replicates is: %s"%len(o_at_least_two_reps)
    print "Here are the sites (hxb2 numbering): %s)"%o_at_least_two_reps_hxb2
    print '+'.join([str(site) for site in o_at_least_two_reps_hxb2])
    print "\nsite\tnhxb2\tcorr_mut_ratio_A\tcorr_mut_ratio_B\tcorr_mut_ratio_C\tWT_amino_acid\tRSA\thydropathy_WT\thydropathy_prefs\tentropy"
    for (site, site_hxb2) in zip(o_at_least_two_reps, o_at_least_two_reps_hxb2):
        site = str(site)
        print "%s\t%s\t%s\t%s\t%s\t%s\t%s\t%s\t%s\t%s"%(site, site_hxb2, corrmutratios[repA][site], corrmutratios[repB][site], corrmutratios[repC][site], prefs[site_hxb2]['WT'], RSAs[site_hxb2], WT_aa_hydropathy[site_hxb2], renormalized_prefs_hydropathy[site_hxb2], rescaled_prefs[site_hxb2]['entropy'])
    
    # Compute a P value using randomization testing to measure the significance of the tripartite overlap.
    nrand = 10000
    P = RandomizationSignificanceForTripartiteOverlap(n1, n2, n3, totn, nrand, obs_overlap)
    
    # Compute the expected distribution
    expected_dist = ComputeExpectedOverlapThreeSamples(totn, n1, n2, n3)

    # Plot the observed and expected distributions
    figure, axes = pylab.subplots(2, 1, figsize = (10, 12))
    matplotlib.rc('font', size=30)
    axes[0].set_title('observed', fontsize = 30, fontweight='bold', y=1.05)
    venn3([set(sites1), set(sites2), set(sites3)], set_labels = ('replicate-%s'%repA, 'replicate-%s'%repB, 'replicate-%s'%repC), ax = axes[0])
    axes[1].set_title('random expectation', fontsize = 30, fontweight='bold', y=1.05)
    venn3(expected_dist, set_labels = ('replicate-%s'%repA, 'replicate-%s'%repB, 'replicate-%s'%repC), ax = axes[1])
    
    # Print the p value
    print "\nP value from the randomization test:"
    print P
    
    pylab.savefig(plotfile, bbox_inches = 'tight')
    pylab.close()
    pdf = plotfile
    png = os.path.splitext(pdf)[0] + '.png'
    !convert -density 192 -trim $pdf $png
    print("\nHere is %s" % png)
    display(Image(png, width=600))
```

In [15]:

```
def PlotSitesAlongPrimarySeq(sites, start_seq, end_seq, plotfile, highlight_sites=[]):
    """
    This function plots the location of sites along a primary sequence
    
    *sites* : a list of sites (integers) to be plotted
    
    *start_seq* and *end_seq* : indices of the first and last sites in the sequence, respectively (integers)
    
    *plotfile* : the name of the output file (must end in .pdf)
    
    *highlight_sites* : same as *sites*, but these sites will be highlighted on the plot
    """
    plotfile = 'temp.pdf'
    pylab.figure(figsize = (10, 1))
    ax = pylab.axes()
    
    for site in sites:
        if site in highlight_sites:
            #print site
            pylab.plot((site, site), (0, 1), 'r', linewidth = 0.25)
        else:
            pylab.plot((site, site), (0, 1), 'k', linewidth = 0.25)

    # Additional plot parameters
    pylab.ylim([0, 1])
    pylab.yticks([])
    pylab.xlim([start_seq, end_seq])
    
    xlabel = 'codon position'
    #ax.set_ylabel(ylabel, fontsize=25)
    ax.set_xlabel(xlabel, fontsize=25)
    ax.yaxis.labelpad = 15
    ax.xaxis.labelpad = 15
    pylab.yticks(fontsize=25)
    pylab.xticks(fontsize=25)
    pylab.rcParams['xtick.major.pad']='10'
    pylab.rcParams['ytick.major.pad']='10'
    ax.xaxis.set_tick_params(width=2.0, length = 5)
    ax.yaxis.set_tick_params(width=2.0, length = 5)
    [i.set_linewidth(2.0) for i in ax.spines.itervalues()]

    # Display the plot
    pylab.savefig(plotfile, bbox_inches = 'tight')
    pylab.close()
    pdf = plotfile
    png = os.path.splitext(pdf)[0] + '.png'
    !convert -density 192 -trim $pdf $png
    print("\nHere is %s" % png)
    display(Image(png, width=900))
```

Make a venn diagram showing the overlap between replicates 1, 2, and 3:

In [16]:

```
PlotThreePartVennDiagram(1, 2, 3, 'venn_123.pdf')
```

```
The number of sites that overlapped between at least two replicates is: 25
Here are the sites (hxb2 numbering): ['48', '62', '64', '65', '66', '81', '105', '162', '188', '203', '207', '212', '377', '420', '433', '436', '443', '557', '558', '560', '564', '588', '591', '653', '655'])
48+62+64+65+66+81+105+162+188+203+207+212+377+420+433+436+443+557+558+560+564+588+591+653+655

site	nhxb2	corr_mut_ratio_A	corr_mut_ratio_B	corr_mut_ratio_C	WT_amino_acid	RSA	hydropathy_WT	hydropathy_prefs	entropy
48	48	3.36109706353	2.10709522993	3.40205049397	A	0.190075767529	1.8	-0.436073912577	2.91567534945
62	62	1.586966509	4.22014265953	8.1565891035	D	0.486939778572	-3.5	-1.61415249248	3.6334993619
64	64	14.4402723927	6.78802912105	10.2774739358	E	0.502647513364	-3.5	-1.61455452939	2.80045463864
65	65	1.27647108666	3.28660429717	3.05407779807	V	0.508354878866	4.2	-2.06451159175	3.33559218268
66	66	6.06867284073	3.0329832719	13.732323907	H	0.523859967716	-3.2	-0.181061822913	3.48123481661
81	81	4.06306364299	4.49061170249	4.13024150233	P	0.523220694311	-1.6	-1.93618885198	2.91988926673
105	105	2.20001272641	3.09820943909	6.96103234046	H	0.027365819209	-3.2	0.69168430052	3.04358951986
167	162	11.2508982522	4.9167435504	1.20592685668	S	0.168013401655	-0.8	-2.64834599619	2.67982357652
193	188	5.8117613498	2.86962290859	4.7793793384	T	0.476024916703	-0.7	-2.06728206325	2.85182547848
208	203	-8.55860684564	6.7129593916	5.36363377506	Q	0.0116760828625	-3.5	0.00813654696101	2.50199352109
212	207	15.6213636267	19.2300238161	21.0750130563	K	0.448984966006	-3.9	2.74320841552	2.68468341481
217	212	5.17953071334	-27.198710775	9.76417903164	P	0.198273105213	-1.6	-0.28903369039	3.40008456764
382	377	10.4761145172	1.06917786741	3.87761067024	N	0.215558452847	-3.5	-2.73642449401	2.21569921577
425	420	2.96286318607	3.8310123648	4.83818560883	I	0.0311164644813	4.5	-1.70288353722	2.82229248404
438	433	7.22477303787	7.21136800895	8.4036179997	A	0.0	1.8	2.24736151015	2.18320121728
441	436	3.09461556807	2.82344283015	5.14925153421	A	0.0	1.8	1.06642026842	2.42611553992
448	443	-5.38468824203	4.16540333662	3.17930358805	I	0.0711233473859	4.5	-0.941624519155	3.39084171525
562	557	4.69893498585	11.359648957	5.55990749259	R	nd	-4.5	0.556842959896	3.54464456163
563	558	2.89840755807	6.02587824332	3.82042531298	A	nd	1.8	-0.727165857575	2.18208315043
565	560	5.91250884227	5.23423793297	6.04124612489	E	nd	-3.5	0.667216157912	3.21545645481
569	564	42.535343543	6.29657133307	-3.9687878919	H	nd	-3.2	-0.805319659704	4.13333596135
593	588	9.36580948653	11.3858296812	11.1559940122	K	0.210936533465	-3.9	1.4752142717	3.03846709018
596	591	6.16770049488	4.16796048083	5.03624482498	Q	0.0350282485876	-3.5	2.19091814814	2.32485989038
658	653	1.62777953831	3.43410410431	3.1900081587	Q	0.467043314501	-3.5	1.68201961798	3.05734199281
660	655	10.7698730795	8.19916740231	4.68639322013	K	0.103897347506	-3.9	1.48586082663	3.277137566

P value from the randomization test:
P $<$ 0.0001

Here is venn_123.png
```

## Use `dms_tools` to analyze changes in mutation frequencies at sites of putative cell-culture adaptation sperately from all other sites in the protein¶

To do so, I will first make new counts files with different subsets of sites based on whether or not the site's error-corrected mutation frequency increased by more than a given threshold upon selection (the threshold is currently >3-fold).

In [17]:

```
def MakeNewCountsFileRetainingSites(file_name, sites_to_retain):
    """This function makes an input file for `dms_editsites`. The input file specifies
    sites to retain in making a new counts file
    
    *file_name* is the name of the file to be created
    
    *sites_to_retain* is a list of sites to retain
    """
    
    # Write sites to retain to a file
    retain_file = open(file_name, 'w')
    retain_file.write('# sites to retain\n')
    for site in sites_to_retain:
        retain_file.write('%s\n'%site)
    retain_file.close()
    
    return None
```

First, I will make new counts files and a mock \*summarystats.txt file for each sample from each replicate:

In [18]:

```
%%capture
print "\nThe counts files will be edited using:"
!dms_editsites -v

# Make a directory to store counts files for subsets of sites
new_counts_files_dir_prefix = 'counts_files_for_sites_sorted_by_delta_mut_freq'
if not os.path.isdir('%s/'%new_counts_files_dir_prefix):
    os.makedirs('%s/'%new_counts_files_dir_prefix)

# Make new "*_counts.txt" files and a mock "*_summarystats.txt" file for each sample from each replicate
mock_summarystats_file = '../replicate-1/DNA-1/DNA-1_summarystats.txt'
alignments = {}
for site_bin in site_bins:
    print "\nBin: %s"%site_bin
    alignments[site_bin] = {}
    for rep in replicates:
        print "\nReplicate: %s"%rep
        
        # Initiate list of input for `dms_summarizealignments`
        alignments[site_bin][rep] = []
        
        # Make an input file for `dms_editsites`
        counts_files_dir = "%s/%s/replicate-%s/"%(new_counts_files_dir_prefix, site_bin, rep)
        if not os.path.isdir(counts_files_dir):
            os.makedirs(counts_files_dir)
        retain_sites_file = "%ssites_to_retain.txt"%(counts_files_dir)
        sites_to_retain = binned_sites[rep][site_bin]
        MakeNewCountsFileRetainingSites(retain_sites_file, sites_to_retain)
        
        for sample in samples[rep]:
            print "\nSample: %s"%sample
            # Record info for `dms_summarizealignments`
            alignprefix = "%s%s_"%(counts_files_dir, sample)
            alignments[site_bin][rep].append('%s,%s'%(alignprefix, sample))
            
            # Make new counts file
            original_countsfile = countsfiles[rep][sample]
            new_countsfile = "%scounts.txt"%(alignprefix)
            cmd_editsites = ' '.join([
                    'dms_editsites',
                    original_countsfile, # input counts file
                    new_countsfile, # output counts file
                    'retain', # edit command
                    retain_sites_file]) # renumbering file
            print ("\nRetaining sites with the command:\n" + cmd_editsites)
            log = !$cmd_editsites
            
            # Make a mock summary stats file
            new_mock_summary_stats_file = "%ssummarystats.txt"%(alignprefix)
            cmd_summarystats = 'cp %s %s'%(mock_summarystats_file, new_mock_summary_stats_file)
            print ("\nMaking a mock summary stats file with the command:\n" + cmd_summarystats)
            log = !$cmd_summarystats
```

Next, I will use `dms_summarizealignments` to generate plots for the different classes of sites. I will do this first for each replicate individually:

In [19]:

```
%%capture
print "\nThe the mutation counts will be summarized using:"
!dms_summarizealignments -v

# Suffixes of files from summarizealignments to delete since they rely on the mock "*_summarizestats.txt" files
delete_suffixes = ['reads.pdf', 'barcodes.pdf', 'depth.pdf', 'mutdepth.pdf']

# Summarize the new counts files
for site_bin in site_bins:
    print "\n############################################"
    print "Processing the bin: %s"%site_bin
    print "############################################"
    pdfs_for_display = []
    for rep in replicates:
        print "\nProcessing replicate: %s"%rep
        outprefix = "%s/%s/replicate-%s/alignmentsummary_"%(new_counts_files_dir_prefix, site_bin, rep)
        cmd_summarizealignments = ' '.join([
                                        'dms_summarizealignments',
                                        outprefix,
                                        'barcodedsubamplicons',
                                        ' '.join(alignments[site_bin][rep]),
                                        '--writemutfreqs'])
        print ("Making alignment summary plots with the command:\n" + cmd_summarizealignments)
        log = !$cmd_summarizealignments
        
        # Make a list of pdfs for display for this bin
        suffix = 'mutfreqs.pdf'
        pdfs_for_display.append(outprefix + suffix)
        
        # Delete files that based on the mock "*_summarystats.txt" files
        for delete_suffix in delete_suffixes:
            cmd_delete = 'rm %s%s'%(outprefix, delete_suffix)
            print ("Deleting file with the command:\n" + cmd_delete)
            !$cmd_delete
```

... and then for all replicates combined:

In [20]:

```
# Make a master plot of mut fracs
print "The the mutation counts will be summarized using:"
!dms_summarizealignments -v

for site_bin in ['corr_mutfreqs_increased', 'corr_mutfreqs_decreased']:
    if 'increased' in site_bin:
        print "\n####################################################################################"
        print "Plot for sites where the error-corrected mutation frequency increased by > %s"%(frac_cutoff)
        print "####################################################################################"
    if 'decreased' in site_bin:
        print "\n####################################################################################"
        print "Plot for sites where the error-corrected mutation frequency decreased or, if it increased, increased by > %s"%(frac_cutoff)
        print "####################################################################################"
    
    # Make a list of input arguments for all replicates for a given bin
    all_alignments = []
    for rep in replicates:
        # So that I don't append the same DNA and mutDNA samples twice for replicate 3b
        if rep == '3b-2':
            all_alignments.extend(alignments[site_bin][rep][2:])
        else:
            all_alignments.extend(alignments[site_bin][rep])
    
    # Make the alignment summaries
    outprefix = "%s/%s/alignmentsummary_"%(new_counts_files_dir_prefix, site_bin)
    cmd_summarizealignments = ' '.join([
            'dms_summarizealignments',
            outprefix,
            'barcodedsubamplicons',
            ' '.join(all_alignments),
            '--writemutfreqs'])
    print ("Making alignment summary plots with the command:\n" + cmd_summarizealignments)
    log = !$cmd_summarizealignments
    
    suffix = 'mutfreqs.pdf'
    pdf = outprefix + suffix
    png = os.path.splitext(pdf)[0] + '.png'
    !convert -density 192 -trim $pdf $png
    print("\nHere is %s" % png)
    display(Image(png, width=500))
```

```
The the mutation counts will be summarized using:
dms_summarizealignments 1.1.dev16

####################################################################################
Plot for sites where the error-corrected mutation frequency increased by > 3.0
####################################################################################
Making alignment summary plots with the command:
dms_summarizealignments counts_files_for_sites_sorted_by_delta_mut_freq/corr_mutfreqs_increased/alignmentsummary_ barcodedsubamplicons counts_files_for_sites_sorted_by_delta_mut_freq/corr_mutfreqs_increased/replicate-1/DNA-1_,DNA-1 counts_files_for_sites_sorted_by_delta_mut_freq/corr_mutfreqs_increased/replicate-1/mutDNA-1_,mutDNA-1 counts_files_for_sites_sorted_by_delta_mut_freq/corr_mutfreqs_increased/replicate-1/virus-1-p2_,virus-1-p2 counts_files_for_sites_sorted_by_delta_mut_freq/corr_mutfreqs_increased/replicate-1/mutvirus-1-p2_,mutvirus-1-p2 counts_files_for_sites_sorted_by_delta_mut_freq/corr_mutfreqs_increased/replicate-2/DNA-2_,DNA-2 counts_files_for_sites_sorted_by_delta_mut_freq/corr_mutfreqs_increased/replicate-2/mutDNA-2_,mutDNA-2 counts_files_for_sites_sorted_by_delta_mut_freq/corr_mutfreqs_increased/replicate-2/virus-2-p2_,virus-2-p2 counts_files_for_sites_sorted_by_delta_mut_freq/corr_mutfreqs_increased/replicate-2/mutvirus-2-p2_,mutvirus-2-p2 counts_files_for_sites_sorted_by_delta_mut_freq/corr_mutfreqs_increased/replicate-3/DNA-3_,DNA-3 counts_files_for_sites_sorted_by_delta_mut_freq/corr_mutfreqs_increased/replicate-3/mutDNA-3_,mutDNA-3 counts_files_for_sites_sorted_by_delta_mut_freq/corr_mutfreqs_increased/replicate-3/virus-3-p1_,virus-3-p1 counts_files_for_sites_sorted_by_delta_mut_freq/corr_mutfreqs_increased/replicate-3/mutvirus-3-p1_,mutvirus-3-p1 counts_files_for_sites_sorted_by_delta_mut_freq/corr_mutfreqs_increased/replicate-3/virus-3-p2_,virus-3-p2 counts_files_for_sites_sorted_by_delta_mut_freq/corr_mutfreqs_increased/replicate-3/mutvirus-3-p2_,mutvirus-3-p2 counts_files_for_sites_sorted_by_delta_mut_freq/corr_mutfreqs_increased/replicate-3b-1/DNA-3b_,DNA-3b counts_files_for_sites_sorted_by_delta_mut_freq/corr_mutfreqs_increased/replicate-3b-1/mutDNA-3b_,mutDNA-3b counts_files_for_sites_sorted_by_delta_mut_freq/corr_mutfreqs_increased/replicate-3b-1/virus-3b-1-p2_,virus-3b-1-p2 counts_files_for_sites_sorted_by_delta_mut_freq/corr_mutfreqs_increased/replicate-3b-1/mutvirus-3b-1-p2_,mutvirus-3b-1-p2 counts_files_for_sites_sorted_by_delta_mut_freq/corr_mutfreqs_increased/replicate-3b-2/virus-3b-2-p2_,virus-3b-2-p2 counts_files_for_sites_sorted_by_delta_mut_freq/corr_mutfreqs_increased/replicate-3b-2/mutvirus-3b-2-p2_,mutvirus-3b-2-p2 --writemutfreqs

Here is counts_files_for_sites_sorted_by_delta_mut_freq/corr_mutfreqs_increased/alignmentsummary_mutfreqs.png
```

```
####################################################################################
Plot for sites where the error-corrected mutation frequency decreased or, if it increased, increased by > 3.0
####################################################################################
Making alignment summary plots with the command:
dms_summarizealignments counts_files_for_sites_sorted_by_delta_mut_freq/corr_mutfreqs_decreased/alignmentsummary_ barcodedsubamplicons counts_files_for_sites_sorted_by_delta_mut_freq/corr_mutfreqs_decreased/replicate-1/DNA-1_,DNA-1 counts_files_for_sites_sorted_by_delta_mut_freq/corr_mutfreqs_decreased/replicate-1/mutDNA-1_,mutDNA-1 counts_files_for_sites_sorted_by_delta_mut_freq/corr_mutfreqs_decreased/replicate-1/virus-1-p2_,virus-1-p2 counts_files_for_sites_sorted_by_delta_mut_freq/corr_mutfreqs_decreased/replicate-1/mutvirus-1-p2_,mutvirus-1-p2 counts_files_for_sites_sorted_by_delta_mut_freq/corr_mutfreqs_decreased/replicate-2/DNA-2_,DNA-2 counts_files_for_sites_sorted_by_delta_mut_freq/corr_mutfreqs_decreased/replicate-2/mutDNA-2_,mutDNA-2 counts_files_for_sites_sorted_by_delta_mut_freq/corr_mutfreqs_decreased/replicate-2/virus-2-p2_,virus-2-p2 counts_files_for_sites_sorted_by_delta_mut_freq/corr_mutfreqs_decreased/replicate-2/mutvirus-2-p2_,mutvirus-2-p2 counts_files_for_sites_sorted_by_delta_mut_freq/corr_mutfreqs_decreased/replicate-3/DNA-3_,DNA-3 counts_files_for_sites_sorted_by_delta_mut_freq/corr_mutfreqs_decreased/replicate-3/mutDNA-3_,mutDNA-3 counts_files_for_sites_sorted_by_delta_mut_freq/corr_mutfreqs_decreased/replicate-3/virus-3-p1_,virus-3-p1 counts_files_for_sites_sorted_by_delta_mut_freq/corr_mutfreqs_decreased/replicate-3/mutvirus-3-p1_,mutvirus-3-p1 counts_files_for_sites_sorted_by_delta_mut_freq/corr_mutfreqs_decreased/replicate-3/virus-3-p2_,virus-3-p2 counts_files_for_sites_sorted_by_delta_mut_freq/corr_mutfreqs_decreased/replicate-3/mutvirus-3-p2_,mutvirus-3-p2 counts_files_for_sites_sorted_by_delta_mut_freq/corr_mutfreqs_decreased/replicate-3b-1/DNA-3b_,DNA-3b counts_files_for_sites_sorted_by_delta_mut_freq/corr_mutfreqs_decreased/replicate-3b-1/mutDNA-3b_,mutDNA-3b counts_files_for_sites_sorted_by_delta_mut_freq/corr_mutfreqs_decreased/replicate-3b-1/virus-3b-1-p2_,virus-3b-1-p2 counts_files_for_sites_sorted_by_delta_mut_freq/corr_mutfreqs_decreased/replicate-3b-1/mutvirus-3b-1-p2_,mutvirus-3b-1-p2 counts_files_for_sites_sorted_by_delta_mut_freq/corr_mutfreqs_decreased/replicate-3b-2/virus-3b-2-p2_,virus-3b-2-p2 counts_files_for_sites_sorted_by_delta_mut_freq/corr_mutfreqs_decreased/replicate-3b-2/mutvirus-3b-2-p2_,mutvirus-3b-2-p2 --writemutfreqs

Here is counts_files_for_sites_sorted_by_delta_mut_freq/corr_mutfreqs_decreased/alignmentsummary_mutfreqs.png
```

## Make my own bar graphs of changes in "error-corrected" mutation frequencies for all sites or subdividing sites into sites of putative cell-culture adaptation vs. other sites¶

For each subset of sites, I will compute "error-corrected" per-codon mutation frequencies using the mutation frequencies reported in the \*mutfreqs.txt files generated above by `dms_tools`. I will then average these mutation frequencies across all sites in a given subset and compare how those average mutation frequencies compare between subsets. First, I will make a test input file to make sure my code is working as expected.

In [21]:

```
test_files_directory = 'test_files/'
if not os.path.isdir(test_files_directory):
    os.makedirs(test_files_directory)
```

In [22]:

```
%%writefile test_files/test_mutfreqs.txt
#sample synonymous nonsynonymous stop_codon 1_nucleotide_mutation 2_nucleotide_mutations 3_nucleotide_mutations
DNA-1 5.11376e-05 0.0001498 1.51254e-05 0.000213561 1.37892e-06 1.12238e-06
mutDNA-1 0.000160582 0.00160057 0.00011653 0.000618354 0.000656673 0.000602652
```

```
Overwriting test_files/test_mutfreqs.txt
```

In [23]:

```
def ReadInMutFreqs(mutfreqs_file):
    """
    This function reads in mutation frequencies from a *mutfreqs.txt file (*mutfreqs_file*) generated by `dms_tools`
    
    Code for doctest:
    >>> mutfreqs_file = 'test_files/test_mutfreqs.txt'
    >>> sample_mutfreqs = ReadInMutFreqs(mutfreqs_file)
    >>> specs = ['synonymous', 'nonsynonymous', 'stop_codon', '1_nucleotide_mutation', '2_nucleotide_mutations', '3_nucleotide_mutations']
    >>> sample = 'DNA-1'
    >>> [sample_mutfreqs[sample][spec] for spec in specs]
    [5.11376e-05, 0.0001498, 1.51254e-05, 0.000213561, 1.37892e-06, 1.12238e-06]
    >>> sample = 'mutDNA-1'
    >>> [sample_mutfreqs[sample][spec] for spec in specs]
    [0.000160582, 0.00160057, 0.00011653, 0.000618354, 0.000656673, 0.000602652]
    """
    mutfreqs_file = open(mutfreqs_file, 'r')
    sample_mutfreqs = {}
    for line in mutfreqs_file:
        line = line.strip()
        if line[0] == '#':
            assert line == '#sample synonymous nonsynonymous stop_codon 1_nucleotide_mutation 2_nucleotide_mutations 3_nucleotide_mutations'
            specs = line[1:].split()
            assert len(specs) == 7
        else:
            sample_data = line.split()
            assert len(sample_data) == len(specs)
            sample = sample_data[0]
            sample_mutfreqs[sample] = dict((specs[i], float(sample_data[i])) for i in range(1, len(sample_data)))
    
    mutfreqs_file.close()
    return sample_mutfreqs
doctest.testmod()
```

Out[23]:

```
TestResults(failed=0, attempted=10)
```

In [24]:

```
def ComputeErrorCorrectedSampleMutFreqs(sample_mutfreqs, sample, control_sample):
    """
    This function computes error-corrected mutation frequences given:
    
    *sample_mutfreqs* A dictionary of mutation frequences generated by the above function *ReadInMutFreqs*
    
    *sample* Is the sample for error correction
    
    *control_sample* Is the sample that will be used to correct the mutation frequencies of the control sample, where:
        corrected_freq = sample_freq - control_sample_freq
    
    Any error-corrected mutation frequencies < 0.0 are set equal to 0.0
    
    The output of this function is a dictionary of error-corrected mutation frequences for the input sample: *sample*
    
    Code for doctest:
    >>> mutfreqs_file = 'test_files/test_mutfreqs.txt'
    >>> sample_mutfreqs = ReadInMutFreqs(mutfreqs_file)
    >>> specs = ['synonymous', 'nonsynonymous', 'stop_codon', '1_nucleotide_mutation', '2_nucleotide_mutations', '3_nucleotide_mutations']
    >>> corr_sample_mutfreqs = ComputeErrorCorrectedSampleMutFreqs(sample_mutfreqs, 'mutDNA-1', 'DNA-1')
    >>> [corr_sample_mutfreqs[spec] for spec in specs]
    [0.00010944439999999999, 0.0014507699999999999, 0.0001014046, 0.00040479299999999997, 0.00065529408, 0.00060152962]
    """
    specs = ['synonymous', 'nonsynonymous', 'stop_codon', '1_nucleotide_mutation', '2_nucleotide_mutations', '3_nucleotide_mutations']
    corrected_sample_mutfreqs = dict((spec, sample_mutfreqs[sample][spec] - sample_mutfreqs[control_sample][spec]) for spec in specs)
    for spec in specs:
        if corrected_sample_mutfreqs[spec] < 0.0:
            corrected_sample_mutfreqs[spec] = 0.0
    
    return corrected_sample_mutfreqs

doctest.testmod()
```

Out[24]:

```
TestResults(failed=0, attempted=15)
```

The below cell computes error-corrected mutation frequencies:

In [25]:

```
# Initiate dictionaries
site_bins_sample_mutfreqs = {}
corr_site_bins_sample_mutfreqs = {}

# Specify wild-type error-correction controls for each mutant library sample
error_correction_samples = {}
for rep in replicates:
    error_correction_samples[rep] = {}
    # ... for mutDNA
    if rep in [1, 2, 3]:
        error_correction_samples[rep]['mutDNA-%s'%rep] = 'DNA-%s'%rep
    elif rep in ['3b-1', '3b-2']:
        error_correction_samples[rep]['mutDNA-3b'] = 'DNA-3b'
    # ... for mutvirus-p1
    if rep == 3:
        error_correction_samples[rep]['mutvirus-%s-p1'%rep] = 'virus-%s-p1'%rep
    # ... for mutvirus-p2
    error_correction_samples[rep]['mutvirus-%s-p2'%rep] = 'virus-%s-p2'%rep

# Read in mutation frequencies and compute "error-corrected" mutation frequencies
for site_bin in site_bins + ['all_sites']:
    site_bins_sample_mutfreqs[site_bin] = {}
    corr_site_bins_sample_mutfreqs[site_bin] = {}
    for rep in replicates:
        # Identify the bin- and replicate-specific mutfreqs.txt input file
        if site_bin == 'all_sites':
            if rep in ['3b-1', '3b-2']:
                all_sites_rep = '3b'
            else:
                all_sites_rep = rep
            site_bin_rep_mutfreqs_file = '../replicate-%s/alignmentsummary_mutfreqs.txt'%all_sites_rep
        else:
            site_bin_rep_mutfreqs_file = "%s/%s/replicate-%s/alignmentsummary_mutfreqs.txt"%(new_counts_files_dir_prefix, site_bin, rep)
        
        # Read mutfreqs.txt into a dictionary for a given site_bin and replicate
        site_bins_sample_mutfreqs[site_bin][rep] = ReadInMutFreqs(site_bin_rep_mutfreqs_file)
        
        # Compute error-corrected mutation frequencies
        corr_site_bins_sample_mutfreqs[site_bin][rep] = {}
        for sample in error_correction_samples[rep]:
            control_sample = error_correction_samples[rep][sample]
            corr_site_bins_sample_mutfreqs[site_bin][rep][sample] = ComputeErrorCorrectedSampleMutFreqs(site_bins_sample_mutfreqs[site_bin][rep], sample, control_sample)
```

Plot error-corrected sample mutation frequencies for all sites and the different subsets of sites:

In [32]:

```
def PlotMutFracsBrokenAxis(sample_mutfreqs, samples, names, modelgroups, rep, plotfile, ylabel, ylim, yticks):
    """
    This function is a modified version of *PlotPairedMutFracs* from *dms_tools.plot.PlotPairedMutFracs*
    
    This version differs from the original `dms_tools` function in the following ways:
        * it takes a dictionary as input instead of a list of counts files
        * makes a plot with only a single set of bars
        * the bar for stop codons is black instead of green
    """
    
    if os.path.splitext(plotfile)[1].lower() != '.pdf':
        raise ValueError("plotfile must end in .pdf: %s" % plotfile)
    if len(sample_mutfreqs) != len(samples):
        raise ValueError("sample_mutfreqs and samples differ in length")
    bar1 = ['synonymous', 'nonsynonymous', 'stop codon']
    d = dict([(key, []) for key in bar1])
    
    # Append data to a list ordered by samples
    for sample in samples:
        for key in bar1:
            if key == 'nonsynonymous':
                d[key].append(sample_mutfreqs[sample]['nonsynonymous'] * 1e3)
            elif key == 'synonymous':
                d[key].append(sample_mutfreqs[sample]['synonymous'] * 1e3)
            elif key == 'stop codon':
                d[key].append(sample_mutfreqs[sample]['stop_codon'] * 1e3)
            else:
                raise ValueError("Invalid key of %s" % key)
    
    matplotlib.rc('text', usetex=True)
    matplotlib.rc('font', size=35)
    matplotlib.rc('legend', fontsize=15)
    matplotlib.rc('xtick', labelsize=35)
    matplotlib.rc('patch', linewidth=0.5)
    
    # Specify locations of bars and lines groups
    barwidth = 0.9 # width of each bar
    widthper = 1.5 * barwidth # width between left sides of bars from DIFFERENT groups
    withingroupspacing = 1.1 * barwidth # width between left sides of bars from SAME groups
    models = samples
    firstmodel = True
    lastgroup = None
    xs = []
    indices = [] # A list specifying the positions of left sides of bars
    for (imodel, igroup) in zip(models, modelgroups):
        if not firstmodel and (igroup == lastgroup):
            xs.append(xs[-1] + withingroupspacing)
            indices.append(indices[-1] + withingroupspacing)
        elif firstmodel:
            firstmodel = False
            xs.append(0) #1 - barwidth + ibar * barwidth)
            indices.append(0.25 * barwidth) # barwidth
        else:
            xs.append(xs[-1] + widthper)
            indices.append(indices[-1] + widthper)
        lastgroup = igroup
    totwidth = indices[-1] + barwidth + 0.25 * barwidth
    
    # Initiate figure
    (lmargin, rmargin, bmargin, tmargin) = (0.07, 0.01, 0.43, 0.05) # Specify inset axes for the bargraph
    fig, (ax1, ax2) = pylab.subplots(2, 1, sharex=True, figsize=(totwidth + lmargin + rmargin, 7.5))
    pylab.subplots_adjust(hspace = .1) # Makes it so there's less space making up the break in the y-axis
    
    # Plot the bars
    bars = [] # bars is a list for keeping track of each bar in the plot, with one per sample (used to be two per sample)
    colors = ['steelblue', '0.75', 'firebrick']# 'brg'
    nsamples = len(samples)
    bottoms = [0] * nsamples # bottoms is a list for keeping track of the cumulative height of each bar, as different keys are processed
    totalheights = [0 for i in range(nsamples)] # For keeping track of the total height of each bar
    for (key, color) in zip(bar1, colors):
        totalheights = [totalheights[i] + d[key][i] for i in range(nsamples)]
        b = ax1.bar(indices, d[key], width=barwidth, bottom=bottoms, color=color) # indices is a list specifying the left sides of bars; d[key] is a list specifying the heights of bars
        b = ax2.bar(indices, d[key], width=barwidth, bottom=bottoms, color=color) # indices is a list specifying the left sides of bars; d[key] is a list specifying the heights of bars
        bars.append(b)
        for i in range(nsamples):
            bottoms[i] += d[key][i]

    # A dictionary specifying group labels for plotting using LaTex formatting
    group_text = {}
    group_text['< 3'] = '$<$ 3'
    group_text['> 3'] = '$>$ 3'
    group_text['all sites'] = 'all sites'
    
    # Plot the group labels
    start_and_end_i_list = []
    for group in set(modelgroups): # group is a common string in modelgroups
        # Index start and end of each type of group in modelgroups
        start_i = min([i for (i, g) in enumerate(modelgroups) if g == group]) # index of first instance of group
        end_i = max([i for (i, g) in enumerate(modelgroups) if g == group]) # index of last instance of group
        start_and_end_i_list.append((start_i, end_i))
        
        # Specify the start and end of the line
        start_x = indices[start_i] / (totwidth)
        end_x = (indices[end_i] + barwidth) / (totwidth)
        line_y = -0.48 # in axes coordinates
        cap_height = 0.03
        line = pylab.Line2D([start_x, end_x], [line_y, line_y], transform=pylab.gca().transAxes, color='black', linewidth=2, solid_capstyle='butt')
        line.set_clip_on(False)
        pylab.gca().add_line(line)
        for x in [start_x, end_x]: # caps on end of lines
            line = pylab.Line2D([x, x], [line_y + cap_height, line_y - cap_height], transform=pylab.gca().transAxes, color='black', linewidth=2, solid_capstyle='butt')
            line.set_clip_on(False)
            pylab.gca().add_line(line)
        pylab.text((start_x + end_x) / 2.0, line_y - 0.04, group_text[group], transform=pylab.gca().transAxes, horizontalalignment='center', verticalalignment='top')
        
    # Plot the bottom line
    bottom_line_text = 'sites where:\n$f^{P2}/f^{DNA}$'
    start_i = start_and_end_i_list[1][0]
    end_i = start_and_end_i_list[2][1]
    start_x = indices[start_i] / (totwidth)
    end_x = (indices[end_i] + barwidth) / (totwidth)
    line_y = line_y - 0.25 # in axes coordinates
    cap_height = 0.03
    line = pylab.Line2D([start_x, end_x], [line_y, line_y], transform=pylab.gca().transAxes, color='black', linewidth=2, solid_capstyle='butt')
    line.set_clip_on(False)
    pylab.gca().add_line(line)
    for x in [start_x, end_x]: # caps on end of lines
        line = pylab.Line2D([x, x], [line_y + cap_height, line_y - cap_height], transform=pylab.gca().transAxes, color='black', linewidth=2, solid_capstyle='butt')
        line.set_clip_on(False)
        pylab.gca().add_line(line)
    pylab.text((start_x + end_x) / 2.0, line_y - 0.04, bottom_line_text, transform=pylab.gca().transAxes, horizontalalignment='center', verticalalignment='top')
    
    # Additional specs
    pylab.rcParams['xtick.major.pad']='11'
    pylab.rcParams['ytick.major.pad']='11'
    pylab.ylabel(ylabel, fontsize = 35, labelpad=15)
    fig.suptitle('replicate %s'%rep, fontsize=20, fontweight='bold')
    [i.set_linewidth(2) for i in ax1.spines.itervalues()]
    [i.set_linewidth(2) for i in ax2.spines.itervalues()]
    ax1.spines['right'].set_visible(False) # hide top and right borders
    ax1.spines['top'].set_visible(False)
    ax1.spines['bottom'].set_visible(False)
    ax2.spines['right'].set_visible(False) # hide top and right borders
    ax2.spines['top'].set_visible(False)
    ax1.xaxis.set_ticks_position('none') # hide top and right ticks
    ax2.xaxis.set_ticks_position('bottom')
    ax1.yaxis.set_ticks_position('left')
    ax2.yaxis.set_ticks_position('left')
    ax2.tick_params(axis='x', direction='out', width = 2) # tick direction
    ax1.tick_params(axis='y', direction='out', width = 2)
    ax2.tick_params(axis='y', direction='out', width = 2)

    # Make the broken axis
    ax1.set_ylim(ylim)  # outliers only
    ax2.set_ylim(0, 2.5)  # most of the data
    d = .015  # how big to make the diagonal lines in axes coordinates
    # arguments to pass plot, just so we don't keep repeating them
    kwargs = dict(transform=ax1.transAxes, color='k', clip_on=False, linewidth=2)
    ax1.plot((-d, +d), (-d, +d), **kwargs)        # top-left diagonal
    kwargs.update(transform=ax2.transAxes)  # switch to the bottom axes
    ax2.plot((-d, +d), (1 - d, 1 + d), **kwargs)  # bottom-left diagonal
    
    # yticker = matplotlib.ticker.MaxNLocator(3)
    # pylab.gca().yaxis.set_major_locator(yticker)
    ax1.set_yticks(yticks)
    ax2.set_yticks([0, 1, 2])
    pylab.xticks([i+barwidth/2 for i in indices], names, rotation=90, fontsize = 35)
    pylab.gca().set_xlim(0, totwidth)
    ax1.set_xlim(0, totwidth)
    ax2.set_xlim(0, totwidth)

    # reorder bar labels since pylab puts them down columns
    barmarks = [b[0] for b in bars]
    barlabels = bar1
    barmarks = [barmarks[2], barmarks[1], barmarks[0]]
    barlabels = [barlabels[2], barlabels[1], barlabels[0]]
    ax1.legend(barmarks, barlabels, handlelength=1.2, ncol=1, frameon=False, bbox_transform=pylab.gcf().transFigure, fontsize=35)
    pylab.ylabel(ylabel)
    pylab.savefig(plotfile, bbox_inches = 'tight')
    pylab.close()
```

In [33]:

```
for rep in [1, 2, 3, '3b-1', '3b-2']:
    
    if rep in [1, 2]:
        names = 3 * ['DNA', 'P2']
        samples = ['mutDNA-%s'%rep, 'mutvirus-%s-p2'%rep]
        modelgroups = 2*['all sites'] + 2*['< 3'] + 2*['> 3']
        
        if rep == 1:
            ylim = (13, 15.5)
            yticks = [13, 14, 15]
        else:
            ylim = (11, 13.5)
            yticks = [11, 12, 13]
    
    elif rep == 3:
        names = 3 * ['DNA', 'P1', 'P2']
        samples = ['mutDNA-%s'%rep, 'mutvirus-%s-p1'%rep, 'mutvirus-%s-p2'%rep]
        modelgroups = 3*['all sites'] + 3*['< 3'] + 3*['> 3']
        ylim = (9.0, 11.5)
        yticks = [9, 10, 11]
    
    else:
        names = 3 * ['DNA', 'P2']
        samples = ['mutDNA-3b', 'mutvirus-%s-p2'%rep]
        modelgroups = 2*['all sites'] + 2*['< 3'] + 2*['> 3']
        if rep == '3b-1':
            ylim = (10.0, 12.5)
            yticks = [10, 11, 12]
        if rep == '3b-2':
            ylim = (8.0, 10.5)
            yticks = [8, 9, 10]
    
    all_samples = []
    d_plot = {}

    for site_bin in ['all_sites', 'corr_mutfreqs_decreased', 'corr_mutfreqs_increased']:
        for sample in samples:
            d_plot['%s_%s'%(site_bin, sample)] = corr_site_bins_sample_mutfreqs[site_bin][rep][sample]
            all_samples.append('%s_%s'%(site_bin, sample))
    
    ylabel = 'error-corrected codon\nmutation frequency $\\times10^{-3}$'
    print "Plotting error-corrected codon mutation frequencies for replicate: %s"%rep
    plotfile = '%s/rep%s_mutfreqs.pdf'%(new_counts_files_dir_prefix, rep)
    PlotMutFracsBrokenAxis(d_plot, all_samples, names, modelgroups, rep, plotfile=plotfile, ylabel=ylabel, ylim=ylim, yticks=yticks)
    pdf = plotfile
    png = os.path.splitext(pdf)[0] + '.png'
    !convert -density 192 -trim $pdf $png
    print("\nHere is %s" % png)
    display(Image(png, width=500))
```

```
Plotting error-corrected codon mutation frequencies for replicate: 1

Here is counts_files_for_sites_sorted_by_delta_mut_freq/rep1_mutfreqs.png
```

```
Plotting error-corrected codon mutation frequencies for replicate: 2

Here is counts_files_for_sites_sorted_by_delta_mut_freq/rep2_mutfreqs.png
```

```
Plotting error-corrected codon mutation frequencies for replicate: 3

Here is counts_files_for_sites_sorted_by_delta_mut_freq/rep3_mutfreqs.png
```

```
Plotting error-corrected codon mutation frequencies for replicate: 3b-1

Here is counts_files_for_sites_sorted_by_delta_mut_freq/rep3b-1_mutfreqs.png
```

```
Plotting error-corrected codon mutation frequencies for replicate: 3b-2

Here is counts_files_for_sites_sorted_by_delta_mut_freq/rep3b-2_mutfreqs.png
```

In [ ]:

```

```
