# Supplementary material for: Experimental Estimation of the Effects of All Amino-Acid Mutations to HIV’s Envelope Protein on Viral Replication in Cell Culture
Source: PLoS Pathog. 2016 Dec 13;12(12):e1006114. doi: 10.1371/journal.ppat.1006114 (PMC5189966; doi:10.1371/journal.ppat.1006114)
Supplement: S3 File — (ZIP) [file ppat.1006114.s013.zip › S3_File_notebooks/correlate_preferences_with_amino_acid_frequencies_in_nature/alignments/CurateLANLMultipleSequenceAlignment.html]

CurateLANLMultipleSequenceAlignment


# Curating a multiple sequence alignment from LANL¶

This IPython notebook curates an alignment from the Los Alamos National Labs HIV sequence database (www.hiv.lanl.gov) in the following ways:

- First, I downloaded multiple sequence alignments of *env* from the database.
- Then, I removed sequences that differed in length from HXB2 or contained a premature stop codon, ambiguous residue, or frame-shift mutation.
- Next, I removed columns in the alignment for which we lacked deep mutational scanning data, columns that had $>$5\% gap characters, or columns in variable loops that appeared poorly aligned by eye. I will further curate this alignment in the IPython notebook: `CorrelatePrefsAndNaturalSequenceDiversity.ipynb`, such as randomly selecting 30 group M sequences per subtype, before using the alignment in the `pyvolve` simulations.
- I will also create a new .txt file of amino-acid preferences averaged between replicates that lack sites corresponding to the low-quality columns I removed above, and where the preferences are renumbered to match the indices of corresponding sites in the alignment with (1, 2, 3, ...) numbering.

Note: this IPython notebook uses `pips`: https://github.com/jbloomlab/pips-1.0

## Imports¶

In [1]:

```
import os
import sys
sys.path.append('../../scripts/')
import prefsutils
import pips.fasta
import pips.align
import doctest
import re
import random
import matplotlib
matplotlib.use('pdf')
%matplotlib inline
import pylab
```

## I downloaded the following alignments of *env* from the HIV sequence database¶

I downloaded the following protein-level and DNA-level alignments of Env from the Los Alamos National Lab's HIV sequence database (http://www.hiv.lanl.gov/).

- *HIV1\_FLT\_2014\_env\_PRO.fasta*
  - Alignment type: Filtered web alignment
  - Year: 2014
  - Organism: HIV-1/SIVcpz
  - DNA/Protein: PRO
  - Region: env
  - Subtype: ALL
  - Format: FASTA
  - Number of sequences: 4173

- *HIV1\_FLT\_2014\_env\_DNA.fasta*
  - Alignment type: Filtered web alignment
  - Year: 2014
  - Organism: HIV-1/SIVcpz
  - DNA/Protein: DNA
  - Region: env
  - Subtype: ALL
  - Format: FASTA
  - Number of sequences: 4173

## Read in starting alignments¶

First, I will read in the alignments and make sure that each pair of protein/DNA FASTA files have the same exact headers in the same order.

In [2]:

```
def AssertHeadersMatch(headers_seqs_1, headers_seqs_2):
    """This function asserts that two FASTA files have the exact same headers in the same order"""
    for i in range(len(headers_seqs_1)):
        if headers_seqs_1[i][0] != headers_seqs_2[i][0]:
            raise ValueError("The headers for entry %s don't match: %s %s"%(i+1, lanl[i][0], dna_lanl[i][0]))
    assert len(headers_seqs_1) == len(headers_seqs_2)
    
    pass
```

In [3]:

```
# Read headers and sequences from the protein alignment
protein_alignment_prefix = 'HIV1_FLT_2014_env_PRO'
lanl = pips.fasta.Read('%s.fasta' %protein_alignment_prefix)
hxb2_lanl = lanl[0]
if hxb2_lanl[0] != 'B.FR.83.HXB2_LAI_IIIB_BRU.K03455':
    raise ValueError("Expected the first sequence to be HXB2")
    
# Read headers and sequences from the DNA alignment
dna_alignment_prefix = 'HIV1_FLT_2014_env_DNA'
dna_lanl = pips.fasta.Read('%s.fasta'%dna_alignment_prefix)
dna_hxb2_lanl = lanl[0]
if dna_hxb2_lanl[0] != 'B.FR.83.HXB2_LAI_IIIB_BRU.K03455':
    raise ValueError("Expected the first sequence to be HXB2")

# Make sure the headers of the DNA and protein alignments match
AssertHeadersMatch(lanl, dna_lanl)
```

## Filter out problematic sequences from the filtered web alignment¶

I will filter out sequences from both the DNA and protein alignments that have one of the following properties as determined using the protein alignment:

- are not the same length as hxb2 (including gaps)
- contain a premature stop codon (i.e. a '\*' character before the final position)
- contain an ambiguous residue ('X')
- contain a frame-shift mutation ('#')

In [4]:

```
def FilterAlignmentbyLengthandDisallowedCharacters(headers_seqs, disallowed_characters, length):
    """
    This function removes sequences from an alignment if they don't match the specified
    length, if they contain any of the specified disallowed characters, or if they have
    premature stop codons.
    
    Code for doctest:
    >>> disallowed_characters = ['#', 'X']
    >>> test_headers_seqs = [('s1', 'A-G-T*'), ('s2', 'AGG-A'), ('s3', 'AGG-A*'), ('s4', 'A-#-T*'), ('s5', 'X-G-T*'), ('s6', 'A*G-T*')] 
    >>> length = len(test_headers_seqs[0][1])
    >>> (a, d) = FilterAlignmentbyLengthandDisallowedCharacters(test_headers_seqs, disallowed_characters, length)
    >>> a
    [('s1', 'A-G-T*'), ('s3', 'AGG-A*')]
    >>> d
    [('s2', 'AGG-A'), ('s4', 'A-#-T*'), ('s5', 'X-G-T*'), ('s6', 'A*G-T*')]
    """
    discarded_seqs = []
    filtered_headers_seqs = []
    for (header, seq) in headers_seqs:
        discard_seq = False
        seq = seq.upper()

        # Filter sequences with premature stop codons
        if '*' in seq[:-1]:
            discard_seq = True

        # Filter sequences with disallowed characters
        for char in disallowed_characters:
            if char in seq:
                discard_seq = True

        # Filter sequences that don't match the specified length
        if len(seq) != length:
            discard_seq = True

        # Append to the filtered alignment sequences that passed the filtering steps
        if discard_seq == False:
            filtered_headers_seqs.append((header, seq))
        else:
            discarded_seqs.append((header,seq))
        
    assert len(headers_seqs) == len(filtered_headers_seqs)+len(discarded_seqs)
    return (filtered_headers_seqs, discarded_seqs)

doctest.testmod()
```

Out[4]:

```
TestResults(failed=0, attempted=6)
```

In [5]:

```
# Filter sequences from the protein alignment
len_hxbii_lanl = len(lanl[0][1])
disallowed_characters = ['#', 'X']
(filtered_lanl, discarded_seqs) = FilterAlignmentbyLengthandDisallowedCharacters(lanl, disallowed_characters, len_hxbii_lanl)

# Next, I will filter the same sequences from the corresponding DNA alignment
discarded_headers = [header for (header, seq) in discarded_seqs]
filtered_dna_lanl = [(header, seq) for (header, seq) in dna_lanl if header not in discarded_headers]

# Make sure the new protein and DNA alignments have the same sequence headers in the same order    
AssertHeadersMatch(filtered_lanl, filtered_dna_lanl)

# Write FASTA files of the protein sequences that passed the filter and those that were discarded
pips.fasta.Write(filtered_lanl, '%s_filteredseqs.fasta'%protein_alignment_prefix)
pips.fasta.Write(discarded_seqs, '%s_discardedseqs.fasta'%protein_alignment_prefix)
pips.fasta.Write(filtered_dna_lanl, '%s_filteredseqs.fasta'%dna_alignment_prefix)
        
# Summary of filtering step
print "Here's a summary of the filtering step"
print "The total number of sequences in the starting alignment is: %s"%(len(lanl))
print "The total number of sequences in the filtered alignment is: %s"%(len(filtered_lanl))
print "The total number of discareded sequences is: %s"%(len(discarded_seqs))
```

```
Here's a summary of the filtering step
The total number of sequences in the starting alignment is: 4173
The total number of sequences in the filtered alignment is: 3842
The total number of discareded sequences is: 331
```

## Remove all columns in the alignment for which HXB2 has a gap character¶

I will remove columns in the filtered web alignments for which the HXB2 sequence has a gap character. Columns will now be numbered by the HXB2 numbering system. This will result in 5 positions in LAI to be removed, but these are in highly variable columns that I don't plan to use in downstream analyses anyways because of how uncertain alignments are in this region.

In [6]:

```
stripped_gaps_lanl = pips.align.StripGapsToFirstSequence(filtered_lanl)
stripped_gaps_dna_lanl = pips.align.StripGapsToFirstSequence(filtered_dna_lanl)

assert len(stripped_gaps_lanl[0][1]) * 3.0 == len(stripped_gaps_dna_lanl[0][1]) # hxbii sequence length comparison, with stop codon/symbol included in both
```

## Make a list of columns in the alignment for which I lack deep mutational scanning data or which fall in low-confidence regions in the alignment¶

Starting from the alignments stripped to the HXB2 sequence, I will compile a list of columns in the alignment that fall into at least one of the following categories and will be masked in downstream analyses:

- columns corresponding to sites for which I have no deep mutational scanning data
  - 1-30 and 703-end
- columns corresponding to all or some of the sites in V1, V2, V4, and V5 that, by eye, look like low-confidence regions due to there being many indels.
  - all of variable loop #1: 132-156
  - part of variable loop #2: 185-190
  - part of variable loop #4: 396-413
  - part of variable loop #5: 460-465
- columns in the alignment where >5% of sequences have a gap where HXB2 (and LAI) have a non-gap character.
  - computationally determined below. Almost all of these columns were columns that I was going to remove anyways for the above reasons.

Ultimately, I will write this list of columns to a .txt file. I will read in the columns from this file in other IPython notebooks in downstream analyses.

In [7]:

```
def IdentifyColumnsInAlignmentWithNumberOfGapsAboveThreshold(headers_seqs, f_threshold):
    """
    This function identifies columns in an alignment where the number of gaps in
    that column is greater than some threshold.
    
    Code for doctest:
    >>> test_headers_seqs = []
    >>> test_headers_seqs.append(('s1', 'A-G-T*'))
    >>> test_headers_seqs.append(('s2', 'AGG--*'))
    >>> test_headers_seqs.append(('s3', 'AC---*'))
    >>> test_headers_seqs.append(('s4', 'AG---*'))
    >>> IdentifyColumnsInAlignmentWithNumberOfGapsAboveThreshold(test_headers_seqs, 0.51)
    [4, 5]
    >>> IdentifyColumnsInAlignmentWithNumberOfGapsAboveThreshold(test_headers_seqs, 0.2)
    [2, 3, 4, 5]
    """
    seq_len = len(headers_seqs[0][1])
    
    # I will count the number of gaps in each column, with columns indexed starting at 1
    ngaps = dict((i+1, 0) for i in range(seq_len))
    for (header, seq) in headers_seqs:
        for i in ngaps:
            if seq[i-1] == '-':
                ngaps[i] += 1
    
    # Next, I will make a list of columns for which the number of gaps exceeds the threshold
    n_threshold = float(f_threshold) * float(len(headers_seqs))
    columns_above_threshold = []
    for i in ngaps:
        if ngaps[i] > n_threshold:
            columns_above_threshold.append(i)

    columns_above_threshold.sort()
    return columns_above_threshold

doctest.testmod()
```

Out[7]:

```
TestResults(failed=0, attempted=13)
```

In [8]:

```
def CreateDNAColumnListFromProteinColumnList(protein_columns):
    """This function takes a list of columns in a protein alignment and returns the corresponding list
    of columns for a DNA alignment of the same gene
    
    Code for doctest:
    >>> test_protein_columns = [1, 3, 8]
    >>> CreateDNAColumnListFromProteinColumnList(test_protein_columns)
    [1, 2, 3, 7, 8, 9, 22, 23, 24]
    """
    dna_columns = []
    for protein_column in protein_columns:
        first_dna_column_in_codon = (3*protein_column)-2
        dna_columns.extend([first_dna_column_in_codon, first_dna_column_in_codon + 1, first_dna_column_in_codon + 2])
    return dna_columns
doctest.testmod()
```

Out[8]:

```
TestResults(failed=0, attempted=15)
```

In [9]:

```
def WriteColumnsToFile(columns, columns_file_name):
    """This function writes a list of columns (*columns*) to a .txt file named *columns_file_name*
    
    Columns will be return-delimited entries.
    """
    
    counts_file = open(columns_file_name, 'w')
    for column in columns:
        counts_file.write('%s\n'%column)
        
    return None
```

In [10]:

```
# A list for columns for removal in the protein alignment
columns_for_removal = []

# I will remove all columns for which I lack deep mutational scanning data
# i.e. 1-30 and 703-856(end)
columns_for_removal.extend([i for i in range(1, 31)])
columns_for_removal.extend([i for i in range(703, 858)]) # including the stop codon

# I will remove all of variable loop #1 between the cysteines (sites 132-156)
columns_for_removal.extend([i for i in range(132, 157)])

# I will remove all of variable loop #2 between the cysteines (sites 185-190)
columns_for_removal.extend([i for i in range(185, 191)])

# I will also remove part of variable loop #4 (sites 396-413)
columns_for_removal.extend([i for i in range(396, 414)])

# and part of variable loop #5 (sites 460-465)
columns_for_removal.extend([i for i in range(460, 466)])

# Additionally, I will remove all columns with >5% gaps for every position in the alignment stripped to HXB2
f_threshold = 0.05
columns_with_excess_gaps = IdentifyColumnsInAlignmentWithNumberOfGapsAboveThreshold(stripped_gaps_lanl, f_threshold)
for i in columns_with_excess_gaps:
    if i not in columns_for_removal:
        columns_for_removal.append(i)
print "The following columns were found to have excess gaps when using a theshold of %s:\n%s" %(f_threshold, columns_with_excess_gaps)

# Make a list of columns for removal in the DNA alignment
dna_columns_for_removal = CreateDNAColumnListFromProteinColumnList(columns_for_removal)

columns_for_removal.sort()
dna_columns_for_removal.sort()
print "\nThe following columns in the protein alignment and equivalent columns in the DNA alignment have been flagged for masking in downstream analysis:\n%s"%columns_for_removal

# Write the columns for removal to an output file for use with other IPython notebooks
columns_for_removal_file_name = 'columns_for_removal_protein_HXB2.txt'
print "\nNow writing the above list of columns in the protein alignment to the file: %s"%columns_for_removal_file_name
WriteColumnsToFile(columns_for_removal, columns_for_removal_file_name)

dna_columns_for_removal_file_name = 'columns_for_removal_DNA_HXB2.txt'
print "\nNow writing the equivalent list of columns in the DNA alignment to the file: %s"%dna_columns_for_removal_file_name
WriteColumnsToFile(dna_columns_for_removal, dna_columns_for_removal_file_name)
```

```
The following columns were found to have excess gaps when using a theshold of 0.05:
[14, 15, 16, 17, 32, 139, 140, 141, 142, 143, 144, 145, 146, 147, 187, 310, 311, 322, 354, 355, 397, 399, 400, 401, 402, 403, 404, 405, 406, 407, 408, 409, 464]

The following columns in the protein alignment and equivalent columns in the DNA alignment have been flagged for masking in downstream analysis:
[1, 2, 3, 4, 5, 6, 7, 8, 9, 10, 11, 12, 13, 14, 15, 16, 17, 18, 19, 20, 21, 22, 23, 24, 25, 26, 27, 28, 29, 30, 32, 132, 133, 134, 135, 136, 137, 138, 139, 140, 141, 142, 143, 144, 145, 146, 147, 148, 149, 150, 151, 152, 153, 154, 155, 156, 185, 186, 187, 188, 189, 190, 310, 311, 322, 354, 355, 396, 397, 398, 399, 400, 401, 402, 403, 404, 405, 406, 407, 408, 409, 410, 411, 412, 413, 460, 461, 462, 463, 464, 465, 703, 704, 705, 706, 707, 708, 709, 710, 711, 712, 713, 714, 715, 716, 717, 718, 719, 720, 721, 722, 723, 724, 725, 726, 727, 728, 729, 730, 731, 732, 733, 734, 735, 736, 737, 738, 739, 740, 741, 742, 743, 744, 745, 746, 747, 748, 749, 750, 751, 752, 753, 754, 755, 756, 757, 758, 759, 760, 761, 762, 763, 764, 765, 766, 767, 768, 769, 770, 771, 772, 773, 774, 775, 776, 777, 778, 779, 780, 781, 782, 783, 784, 785, 786, 787, 788, 789, 790, 791, 792, 793, 794, 795, 796, 797, 798, 799, 800, 801, 802, 803, 804, 805, 806, 807, 808, 809, 810, 811, 812, 813, 814, 815, 816, 817, 818, 819, 820, 821, 822, 823, 824, 825, 826, 827, 828, 829, 830, 831, 832, 833, 834, 835, 836, 837, 838, 839, 840, 841, 842, 843, 844, 845, 846, 847, 848, 849, 850, 851, 852, 853, 854, 855, 856, 857]

Now writing the above list of columns in the protein alignment to the file: columns_for_removal_protein_HXB2.txt

Now writing the equivalent list of columns in the DNA alignment to the file: columns_for_removal_DNA_HXB2.txt
```

## Make new alignments without low-quality columns and a preferences files with the same sites removed and the remaining sites renumbered sequentially.¶

I will now remove the above low-quality columns from the alignment. I will also use `dms_editsites` to make new a new file of amino-acid preferences averaged between replicates with the same sites removed and the remaining sites sequentially renumbered to match the index of the remaining columns in the alignment with (1, 2, 3, ...) numbering. After futher curation of this alignment in the IPython notebook: `CorrelatePrefsAndNaturalSequenceDiversity.ipynb`, I will use the resulting alignment and matching preferences files in the `pyvolve` simulation.

In [11]:

```
def RemoveColumnsFromAlignment(headers_seqs, columns_for_removal):
    """
    This function removes columns from an alignment
    
    Code for doctest:
    >>> test_headers_seqs = [('s1', 'A-G-T*'), ('s2', 'AGG--*'), ('s3', 'ACTT-*'), ('s4', 'AG---*')]
    >>> RemoveColumnsFromAlignment(test_headers_seqs, [1, 4, 6])
    [('s1', '-GT'), ('s2', 'GG-'), ('s3', 'CT-'), ('s4', 'G--')]
    """
    shortened_headers_seqs = []
    for (header, seq) in headers_seqs:
        shortened_seq = []
        for i in range(len(seq)):
            if i+1 not in columns_for_removal:
                shortened_seq.append(seq[i])
        assert len(seq)-len(columns_for_removal) == len(shortened_seq)
        shortened_headers_seqs.append((header, ''.join(shortened_seq)))
    
    return (shortened_headers_seqs)

doctest.testmod()
```

Out[11]:

```
TestResults(failed=0, attempted=17)
```

In [12]:

```
def Create_Remove_And_Renumber_Files_For_dms_editsites(columns_for_removal, removefile, renumberfile):
    """
    This function creates two files for *dms_editsites*. These files are for removing and renumbering
    sites in .txt files of preferences so that they match alignments after removal of a subset of columns:
    
    *removefile* is a file that specifies sites for removal
    
    *renumberfiles* is a file that specifies sites for renumbering
    """
    # First, I will make a file specifying which sites in HXBII to remove.
    # Specifically, I will remove all sites in "columns_for_removal"
    dms_remove = open(removefile, 'w')
    dms_remove.write('# sites to remove\n')
    for site in columns_for_removal:
        if (site > 30) and (site < 703):
            dms_remove.write('%s\n'%site)
    # I will also remove the sites in LAI that lack homologs in HXB2
    for site in ['142a', '142b', '142c', '142d', '142e']:
        dms_remove.write('%s\n'%site)

    # Next, I will make a file to renumber the remaing sites in sequential order using 1, 2, ... numbering.
    dms_renumber = open(renumberfile, 'w')
    dms_renumber.write('#ORIGINAL_SITE NEW_SITE\n')
    n = 1 # indexed starting at 1
    for site in range(31, 703):
        if site not in columns_for_removal:
            dms_renumber.write('%s %s\n'%(site, n))
            n += 1
    
    return None
```

Make a new alignment, NOT including columns flagged for removal (see above). Also, make new file of the preferences averaged between replicates, NOT including sites corresponding to the removed columns, and renumbered to match the indices of the remaining sites in the alignment.

In [13]:

```
print "Edited preferences files will be generated using:"
!dms_editsites -v

# Preferences file for renumbering
prefs_file = '../../averaged_preferences/avg_prefs_p2_nhxb2.txt'

# Subdivide Env
sequence_elements = {}
sequence_elements['Env'] = [i for i in range(31, 703)]

# Make a directory for the renumbered prefs files
renumbered_prefs_dir_prefix = 'renumbered_prefs'
if not os.path.isdir('%s/'%renumbered_prefs_dir_prefix):
    os.makedirs('%s/'%renumbered_prefs_dir_prefix)

# Make subalignments, masking regions previously identified as low confidence
sub_stripped_gaps_lanl = {}
sub_stripped_gaps_dna_lanl = {}
for element in sequence_elements:
    
    # Remove columns that are not in the element or that have already been flagged for removal
    sub_columns_for_removal = [i for i in range(1, 858) if (i not in sequence_elements[element]) or (i in columns_for_removal)]
    
    # Make a list of columns for removal in the DNA alignment
    dna_sub_columns_for_removal = CreateDNAColumnListFromProteinColumnList(sub_columns_for_removal)
    
    # Remove the specified columns from the DNA and protein alignments and make new FASTA files
    print "The following columns in the protein alignment (and equivalent columns in the DNA alignment) will now be removed:\n%s"%columns_for_removal
    sub_stripped_gaps_lanl[element] = RemoveColumnsFromAlignment(stripped_gaps_lanl, sub_columns_for_removal)
    sub_stripped_gaps_dna_lanl[element] = RemoveColumnsFromAlignment(stripped_gaps_dna_lanl, dna_sub_columns_for_removal)
    
    print "\nWriting the protein and DNA alignments to the following files:"
    current_protein_alignment_file = '%s_filteredseqs_strippedgapstoHXB2_%s_without_lowq_columns.fasta'%(protein_alignment_prefix, element)
    current_dna_alignment_file = '%s_filteredseqs_strippedgapstoHXB2_%s_without_lowq_columns.fasta'%(dna_alignment_prefix, element)
    print current_protein_alignment_file
    print current_dna_alignment_file
    pips.fasta.Write(sub_stripped_gaps_lanl[element], current_protein_alignment_file)
    pips.fasta.Write(sub_stripped_gaps_dna_lanl[element], current_dna_alignment_file)
    
    # Make sure the HXB2 sequence in the DNA alignment encodes the HXB2 sequence in the protein alignment
    current_hxb2_protein_seq = sub_stripped_gaps_lanl[element][0][1]
    translated_current_hxb2_dna_seq = pips.fasta.Translate([sub_stripped_gaps_dna_lanl[element][0]])[0][1]
    assert current_hxb2_protein_seq == translated_current_hxb2_dna_seq
    
    # Make dms_editsites files for removing and renumbering sites in the preferences so that they match the subalignment 
    removefile = '%s/phydms_remove_%s.txt'%(renumbered_prefs_dir_prefix, element)
    renumberfile = '%s/phydms_renumber_%s.txt'%(renumbered_prefs_dir_prefix, element)
    print "\nNow writing the following input files for dms_editsites:\n%s\n%s"%(removefile, renumberfile)
    Create_Remove_And_Renumber_Files_For_dms_editsites(sub_columns_for_removal, removefile, renumberfile)
    
    # Make new prefs files with the appropriate sites removed
    print "\nNow making a reunumber preferences file starting from the file: %s"%prefs_file
    cmd = ' '.join([
            'dms_editsites',
            prefs_file, # infile
            '%s/avg_prefs_p2_nhxb2_removed_%s.txt'%(renumbered_prefs_dir_prefix, element), # outfile
            'remove',
            removefile # editifile
            ])
    print ("Removing sites with the command:\n" + cmd)
    log = !$cmd
    
    # Make new prefs files with the remaining sites renumbered in sequential order (1, 2, ...)
    cmd = ' '.join([
            'dms_editsites',
            '%s/avg_prefs_p2_nhxb2_removed_%s.txt'%(renumbered_prefs_dir_prefix, element),
            '%s/avg_prefs_p2_nhxb2_renumbered_%s.txt'%(renumbered_prefs_dir_prefix, element),
            'renumber',
            renumberfile
            ])
    print ("Renumbering the remaining sites with the command:\n" + cmd)
    log = !$cmd
```

```
Edited preferences files will be generated using:
dms_editsites 1.1.dev13
The following columns in the protein alignment (and equivalent columns in the DNA alignment) will now be removed:
[1, 2, 3, 4, 5, 6, 7, 8, 9, 10, 11, 12, 13, 14, 15, 16, 17, 18, 19, 20, 21, 22, 23, 24, 25, 26, 27, 28, 29, 30, 32, 132, 133, 134, 135, 136, 137, 138, 139, 140, 141, 142, 143, 144, 145, 146, 147, 148, 149, 150, 151, 152, 153, 154, 155, 156, 185, 186, 187, 188, 189, 190, 310, 311, 322, 354, 355, 396, 397, 398, 399, 400, 401, 402, 403, 404, 405, 406, 407, 408, 409, 410, 411, 412, 413, 460, 461, 462, 463, 464, 465, 703, 704, 705, 706, 707, 708, 709, 710, 711, 712, 713, 714, 715, 716, 717, 718, 719, 720, 721, 722, 723, 724, 725, 726, 727, 728, 729, 730, 731, 732, 733, 734, 735, 736, 737, 738, 739, 740, 741, 742, 743, 744, 745, 746, 747, 748, 749, 750, 751, 752, 753, 754, 755, 756, 757, 758, 759, 760, 761, 762, 763, 764, 765, 766, 767, 768, 769, 770, 771, 772, 773, 774, 775, 776, 777, 778, 779, 780, 781, 782, 783, 784, 785, 786, 787, 788, 789, 790, 791, 792, 793, 794, 795, 796, 797, 798, 799, 800, 801, 802, 803, 804, 805, 806, 807, 808, 809, 810, 811, 812, 813, 814, 815, 816, 817, 818, 819, 820, 821, 822, 823, 824, 825, 826, 827, 828, 829, 830, 831, 832, 833, 834, 835, 836, 837, 838, 839, 840, 841, 842, 843, 844, 845, 846, 847, 848, 849, 850, 851, 852, 853, 854, 855, 856, 857]

Writing the protein and DNA alignments to the following files:
HIV1_FLT_2014_env_PRO_filteredseqs_strippedgapstoHXB2_Env_without_lowq_columns.fasta
HIV1_FLT_2014_env_DNA_filteredseqs_strippedgapstoHXB2_Env_without_lowq_columns.fasta

Now writing the following input files for dms_editsites:
renumbered_prefs/phydms_remove_Env.txt
renumbered_prefs/phydms_renumber_Env.txt

Now making a reunumber preferences file starting from the file: ../../averaged_preferences/avg_prefs_p2_nhxb2.txt
Removing sites with the command:
dms_editsites ../../averaged_preferences/avg_prefs_p2_nhxb2.txt renumbered_prefs/avg_prefs_p2_nhxb2_removed_Env.txt remove renumbered_prefs/phydms_remove_Env.txt
Renumbering the remaining sites with the command:
dms_editsites renumbered_prefs/avg_prefs_p2_nhxb2_removed_Env.txt renumbered_prefs/avg_prefs_p2_nhxb2_renumbered_Env.txt renumber renumbered_prefs/phydms_renumber_Env.txt
```

In [ ]:

```

```
