# Supplementary material for: Experimental Estimation of the Effects of All Amino-Acid Mutations to HIV’s Envelope Protein on Viral Replication in Cell Culture
Source: PLoS Pathog. 2016 Dec 13;12(12):e1006114. doi: 10.1371/journal.ppat.1006114 (PMC5189966; doi:10.1371/journal.ppat.1006114)
Supplement: S3 File — (ZIP) [file ppat.1006114.s013.zip › S3_File_notebooks/correlate_preferences_with_amino_acid_frequencies_in_nature/SimulateExpectedCorrelation.html]

SimulateExpectedCorrelation


# Use `pyvolve` to simulate Env's evolution using a preferences-based model to determine the expected correlation between natural sequences and the preferences.¶

The workflow of this notebook is:

- use RAxML to infer a phylogenetic tree for an alignment of env sequences
- estimate the expected nucleotide composition in the absence of amino-acid selection
- estimate the transition-transversion ratio kappa from the literature
- use `pyvolve` to computationally simulate the evolution along this tree assuming that the rescaled preferences are the true ones and using the above model parameters
- run 100 simulations to get a range on the expected correlations

## Imports¶

In [2]:

```
import os
import sys
sys.path.append('../scripts')
import prefsutils
import plotutils
import pips.fasta
import numpy
import scipy.stats
import random
import doctest
from IPython.display import Image, display
```

## Use RAxML to make a phylogenetic tree for sequences in a given alignment¶

I will use an alignment of group M sequences with 30 sequences per subtype from subtypes A, B, C, D, F, and G. I made this alignment in the IPython notebook called `CorrelatePrefsAndNaturalSequenceDiversity.ipynb`.

In [3]:

```
env_pro_alignment_file_name = 'alignments/HIV1_FLT_2014_env_PRO_filteredseqs_strippedgapstoHXB2_Env_without_lowq_columns_clade_grpM_30perclade.fasta'
env_DNA_alignment_file_name = 'alignments/HIV1_FLT_2014_env_DNA_filteredseqs_strippedgapstoHXB2_Env_without_lowq_columns_clade_grpM_30perclade.fasta'

!raxml -s {env_DNA_alignment_file_name} -n jukescantor -m GTRCAT -p 1 -T 2
```

## Estimate the expected nucleotide composition in the absence of selection at the amino-acid level¶

I will do this using an alignment of group M env gene sequences I made using the IPython notebook: `CorrelatePrefsAndNaturalSequenceDiversity.ipynb`. This alignment is given above (*DNA\_alignment\_file*) and consists of 30 sequences per subtype from subtypes A, B, C, D, F, and G, randomly selected from the starting LANL group M alignment for which I removed columns that were low quality because of frequent indels.

Specifically, I will examine amino acids with four-fold degeneracy at the third codon position. For positions in Env where one of these amino acids is the most frequent amino acid in the alignment, I will count the number of times each of the four nucleotides is found at the third position (making sure the first two nucleotides are the same since some amino acids are encoded by more than four codons). Then, after summing nucleotide counts over all sites, I will compute the relative fraction of each nucleotide, and take this fraction to be the relative nucleotide preference for the entire gene. The assumption here is that selection for different synonynous codons is acting on the nucleotide level and will reflect nucleotide preferences in tha absence of amino-acid selection. I excluded codons in the Rev-response element, since there is selection for specific codons that satisfy 2ndary structre constraints.

In [5]:

```
# Read in the sequences from an alignment
env_DNA_alignment = pips.fasta.Read(env_DNA_alignment_file_name)
env_pro_alignment = pips.fasta.Read(env_pro_alignment_file_name)
```

In [6]:

```
# Compute site-specific amino-acid frequencies from the protein alignment
amino_acid_frequencies = prefsutils.ComputeSiteSpecificAminoAcidFrequenciesInAlignment(env_pro_alignment)

# Specify which amino-acids have four-fold degeneracy at their third position, and if so, what the first
# two non-degenerate nucleotides are (since some amino acids are also encoded by degeneracies in the first
# and second positions, as well)
AAs_with_four_fold_degeneracy = {
                                'L':'CT',
                                'V':'GT',
                                'S':'TC',
                                'P':'CC',
                                'T':'AC',
                                'A':'GC',
                                'R':'CG',
                                'G':'GG'}

degenerate_nt_counts = dict((nt, 0) for nt in ['A', 'T', 'G', 'C'])
n_sites_analyzed = 0

for codon_site in amino_acid_frequencies:
    
    # Ignore codons in the Rev-response element (495-613 in HXB2 numbering). In this alignment, which lacks low-quality
    # columns, the sites that fall witin the RRE are sites 404-522 (see the file `alignments/renumbered_prefs/phydms_renumber_Env.txt`)
    if 404 <= codon_site <= 522:
        continue
    
    # For each site, determine the most common amino acid at that position (sites indexed starting at 1, e.g.: 1, 2, 3, ...)
    freq_aa = [(amino_acid_frequencies[codon_site][aa], aa) for aa in amino_acid_frequencies[codon_site]]
    freq_aa.sort()
    most_freq_aa = freq_aa[-1][1]
    
    # If the most common amino acid has four-fold degeneracy at the third position, then analyze it further    
    if most_freq_aa not in AAs_with_four_fold_degeneracy:
        continue
    n_sites_analyzed += 1
    
    # For all sequences with the amino acid of interest, count the number of times each nucleotide occurs at the
    # third position, making sure that the first two nucleotides are the expected nucleotides.
    first_nt_site = 3*codon_site - 2 # index of the first nt of the site's codon (nt sites indexed starting at 1, e.g.: 1, 2, 3, ...)
    
    for (header, DNA_seq) in env_DNA_alignment:
        codon = DNA_seq[first_nt_site-1:first_nt_site-1+3]
        codon = codon.upper()
        
        # Skip codons that are degenerate at the first and/or second positions
        if codon[:2] == AAs_with_four_fold_degeneracy[most_freq_aa]:      
            degenerate_nt_counts[codon[2]] += 1

print "\nThe total number of sites used to determine phi values for each nucleotide was: %s" %n_sites_analyzed
print "\nHere are the counts for each nucleotide:"
for nt in ['A', 'T', 'G', 'C']:
    print "%s = %s"%(nt, degenerate_nt_counts[nt])

total_nt_counts = sum(degenerate_nt_counts.values())
psi = dict((nt, float(degenerate_nt_counts[nt])/total_nt_counts) for nt in degenerate_nt_counts)
print "\nHere is the fraction of each nucleotide:"
for nt in ['A', 'T', 'G', 'C']:
    print "%s = %s"%(nt, psi[nt])
```

```
The total number of sites used to determine phi values for each nucleotide was: 219

Here are the counts for each nucleotide:
A = 14688
T = 4898
G = 2976
C = 4059

Here is the fraction of each nucleotide:
A = 0.551744863078
T = 0.183990083017
G = 0.111791442846
C = 0.152473611059
```

## Estimate the transition-transversion ratio kappa from the literature¶

I will use the kappa value estimated from the Nielsen and Yang 1998 study cited below (=4.3674). This value is in close agreement with an estimate of kappa from the Sharp et al. 2000 study.

- Nielsen and Yang. GENETICS March 1, 1998 vol. 148 no. 3 929-936

  - kappa = 4.3674. This study investigated 77 codons of V3 from HIV isolates from a single patient over time and compared several maximum-likelihood phylogenetic models in describing the evolution of these sequences. The above estimate of kappa is from the positive selection model from Table 1. As the authors note, there is more variability in kappa estimations between years than between models.
- Sharp et al. Biochem Soc Trans. 2000 Feb;28(2):275-82.

  - kappa = 4.7. This study used maximum-likelihood phylogenetic modeling of several group-M sequences to estimate kappa for env, pol, and gag for codon positions 1, 2, and 3. The estimate for env averaged across all codon positions was 4.7. This model also involved estimating the alpha parameter of a gamma distribution for rate heterogeneity among sites.

## Write model parameters to a file¶

I will use the stringency parameter that maximizes the correlation between the averaged preferences and the group M alignment (the same preferences and alignment I'm using in the this anaysis) (=2.1156) as determined in the IPython notebook: `CorrelatePrefsAndNaturalSequenceDiversity.ipynb`

In [7]:

```
%%writefile env_modelparams.txt
stringencyparameter = 2.1156
kappa = 4.3674
phiA = 0.55
phiT = 0.18
phiG = 0.11
phiC = 0.15
```

```
Overwriting env_modelparams.txt
```

## Use `pyvolve` to simulate the evolution along this tree assuming the preferences are the true ones¶

In [8]:

```
def ComputePercentIdentityFromFASTA(query_seq, headers_seqs):
    """
    This function computes the percent identity between a query sequence and sequences from a FASTA file
    in the form of a list of tupples (header, sequence) described above.
    
    *query_seq* : is the sequence of the query
    
    Non-matching gap characters are counted as mismatches. However, matching gap characters are NOT counted as
    matches and these sites are not considered when calculating the percent ID.
    
    Code for doctest:
    >>> query_seq = 'atT-cAg'
    >>> headers_seqs = [('seq1', 'ATt-cag'), ('seq2', 'aat-cag'), ('seq3', 'att--ag'), ('seq4', 'attgcag'), ('seq5', 'tcg-gga'), ('seq6', 'gat-gag')]
    >>> percentID = ComputePercentIdentityFromFASTA(query_seq, headers_seqs)
    >>> percentID['seq1']
    1.0
    >>> percentID['seq2']
    0.8333333333333334
    >>> percentID['seq3']
    0.8333333333333334
    >>> percentID['seq4']
    0.8571428571428571
    >>> percentID['seq5']
    0.0
    >>> percentID['seq6']
    0.5
    """
    percentID = {}
    query_seq = query_seq.upper()
    for (header, seq) in headers_seqs:
        assert len(query_seq) == len(seq)
        seq = seq.upper()
        nmatches = 0
        nmatchinggaps = 0
        for i in range(len(query_seq)):
            if seq[i] == query_seq[i]:
                nmatches += 1
                if seq[i] == '-':
                    nmatchinggaps += 1     
        percentID[header] = float(nmatches - nmatchinggaps)/float(len(seq)-nmatchinggaps)
    
    return percentID

doctest.testmod()
```

Out[8]:

```
TestResults(failed=0, attempted=9)
```

In [9]:

```
def ComputeAveragePairwiseIdentitesInAlignment(headers_seqs):
    """
    This function computes the average percent identity between sequences in a given alignment in the form of a
    list of tupples (header, sequence) as described above.
    
    Test code for doctest:
    >>> headers_seqs = [('seq1', 'ATt-cag'), ('seq2', 'aat-cag'), ('seq3', 'att--ag'), ('seq4', 'attgcag'), ('seq5', 'tcg-gga'), ('seq6', 'gat-gag')]
    >>> avg_percentID = ComputeAveragePairwiseIdentitesInAlignment(headers_seqs)
    >>> round(avg_percentID, 9)
    0.458730159
    """
    percentIDs = {} # percentIDs[header_i][header_j] returns the percent identity between these two headers
    for (header, seq) in headers_seqs:
        percentIDs[header] = ComputePercentIdentityFromFASTA(seq, headers_seqs)
    
    # Make a list of all pairwise distances
    pairwise_percentIDs = []
    for i in range(len(headers_seqs)):
        header_i = headers_seqs[i][0]
        for j in range(len(headers_seqs)):
            header_j = headers_seqs[j][0]
            if i == j:
                assert percentIDs[header_i][header_j] == 1.0
            elif i >= j:
                continue
            else:
                pairwise_percentIDs.append(percentIDs[header_i][header_j])
    
    # Return the average pairwise distance
    avg_percentID = numpy.mean(pairwise_percentIDs)
    return avg_percentID

doctest.testmod()
```

Out[9]:

```
TestResults(failed=0, attempted=12)
```

In [10]:

```
def PlotCumulativeFraction(xvalues, plotfile='temp.pdf'):
    """
    This function makes a cumulative fraction plot for a given list of x variables: *xvalues*
    """
    
    xvalues.sort()
    yvalues = []
    cumulative_fraction = 0.0
    for xvalue in xvalues:
        cumulative_fraction += 1/float(len(xvalues))
        yvalues.append(cumulative_fraction)
    assert len(xvalues) == len(yvalues)
    
    pylab.figure(figsize=(15,5))
    ax = pylab.axes()
    pylab.plot(xvalues, yvalues)
    ylabel = 'Cumulative fraction'
    xlabel = 'PercentID'
    ax.set_ylabel(ylabel, fontsize=20)
    ax.set_xlabel(xlabel, fontsize=20)
    pylab.xlim([0.7, 1])
    pylab.ylim([0, 1])
    pylab.yticks(fontsize=15)
    pylab.xticks(fontsize=15)
    pylab.savefig(plotfile, bbox_inches = 'tight')
    # pylab.close()
    
    pdf = plotfile
    png = os.path.splitext(pdf)[0] + '.png'
    !convert -density 192 -trim $pdf $png
    print("\nHere is %s" % png)
    display(Image(png, width=900))
```

In [11]:

```
def RunSbatchCmd(cmd, sbatchfilename):
    """
    This function makes an sbatch file given:
        *cmd* a command for the sbatch file
        *sbatchfilename* the name of the sbatch file
    """
    sbatchfile= open(sbatchfilename, 'w')
    sbatchfile.write("#!/bin/sh\n")
    sbatchfile.write("#SBATCH\n")
    sbatchfile.write("#PBS -l walltime=96:00:00\n")
    sbatchfile.write(cmd)
    sbatchfile.close()
    sbatchcmd = 'sbatch %s'%sbatchfilename
    print "Running the job using sbatch with the input file: %s"%sbatchfilename
    print "... and the command: %s"%sbatchcmd
    !$sbatchcmd
    return None
```

In [12]:

```
def SimulateNewAlignment(prefs_file, tree_file, modelparams, simulated_alignment_file_name, scalerate, sbatchfilename):
    """
    This function simulates an alignment using `pyvolve` using the program `run_simulation.py`. It generates a .fasta
    file with the alignment and then reports the average pairwise distance between sequences in the alignment.
    
    Input:
        *prefs_file* : a file of amino-acid preferences
        
        *tree_file* : a file of the input tree for the simulation
        
        *modelparams* : a file of model parameters created in the main script
        
        *simulated_alignment_file_name* : the name of the output .fasta file with the simulated alignment
        
        *scalerate* : a parameter (float) that scales the evolutionary rate
    """
    
    run_simulation_cmd = ' '.join([
                        'python',
                        'run_simulation.py',
                        prefs_file, # prefs_file
                        tree_file, # tree_file
                        modelparams, # modelparams
                        simulated_alignment_file_name, # simulated_alignment
                        '--scalerate %s'%scalerate
                        ])

    print ("Simulating an alignment using the command:\n"+ run_simulation_cmd)
    
    # Run this command if you wish to use sbatch
    #RunSbatchCmd(run_simulation_cmd, sbatchfilename)
    
    # Run this command if you wish to use the terminal and not sbatch
    !$run_simulation_cmd

    pass
```

First, I will determine the average pairwise distance between sequences in the real alignment. I will then emperically determine a scalerate parameter that results in similar distances in the simulated alignments.

In [13]:

```
avg_percentID_DNA = ComputeAveragePairwiseIdentitesInAlignment(env_DNA_alignment)
avg_percentID_pro = ComputeAveragePairwiseIdentitesInAlignment(env_pro_alignment)
print "The average pairwise identity for the real input alignments are:"
print "DNA alignment: %s"%avg_percentID_DNA
print "protein alignment: %s"%avg_percentID_pro
```

```
The average pairwise identity for the real input alignments are:
DNA alignment: 0.843091093562
protein alignment: 0.797994827124
```

The below table shows how the parameter *scalerate* influences the average pairwise identity of sequences in the simulated alignment. Values for *scalerate* that have multiple values for average pairwise identity are ones for which I ran multiple simulations.

```
scalerate -> average pairwise identity
0.1 -> 0.97
0.4 -> 0.89
0.5 -> 0.87, 0.86, 0.86
0.6 -> 0.85, 0.84
0.7 -> 0.81, 0.82
0.8 -> 0.80
2.0 -> 0.67
```

Based on these results, I will set the *scalerate* parameter to 0.6

# Conduct the simulations with `pyvolve`¶

Input for the `pyvolve` simulation:

- **site-specific amino-acid preferences** : averaged between replicates with sites that aren't in the alignment removed from the preferences file and the file nenumbered using sequential numbering (1, 2, 3, ...)
- **stringency parameter** : the one that maximizes the correlation between the amino-acid preferences and amino-acid frequencies in the alignment used to build the tree (see the notebook: `CorrelatePrefsAndNaturalSequenceDiversity.ipynb`). This parameter is specified in the `env_modelparams.txt` input file created above
- **phylogenetic tree** : the phylogenetic tree created above using `RAxML`
- **scale rate** : the parameter emperically determined from above
- **expected nucleotide frequencies and kappa** : specified in the above `env_modelparams.txt` input file

In [15]:

```
# Specify input
prefs_file = 'alignments/renumbered_prefs/avg_prefs_p2_nhxb2_renumbered_Env.txt'
tree_file = 'RAxML_result.jukescantor'
modelparams = 'env_modelparams.txt' # file containing model params from phydms
scalerate = 0.6
```

I will use the script `run_simulation.py` to conduct 100 simulations using `pyvolve`:

In [16]:

```
# Specify the name of the file with the simulated alignment
simulated_alignment_dir_prefix = 'simulated_alignments'
if not os.path.isdir('%s/'%simulated_alignment_dir_prefix):
    os.makedirs('%s/'%simulated_alignment_dir_prefix)

# Emperically find a scalerate parameter that gives the same pairwise distance as that seen in the real alignment
n_simulations = 100
for i in range(n_simulations):
    simulated_alignment_file_name = '%s/simulated_alignment_sim_%s.fasta'%(simulated_alignment_dir_prefix, i) # Name of created alignment file
    sbatchfilename = '%s/run.sbatch_sim_%s'%(simulated_alignment_dir_prefix, i)
    SimulateNewAlignment(prefs_file, tree_file, modelparams, simulated_alignment_file_name, scalerate, sbatchfilename)
    i += 1
```

## Conduct negative-control simulations¶

I will conduct two types of negative controls. The first negative control will be to conduct simulations after randomly shuffling the preferences between sites. The second negative control will be to conduct simulations after averaging the preferences across sites. Both controls would be expected to generate alignments that have very low correlations with the starting site-specific preferences, indicating that correlations with the above simulations are dependent on the site-specificity of the preferences.

First, I will conduct simulations with preferences randomly shuffled between sites, starting by shuffling the preferences:

In [17]:

```
# Randomly shuffle the preferences between sites
prefs = prefsutils.ConvertPrefsFileToDictionary(prefs_file, CIs_present=False)
sites_to_shuffle = prefs.keys()
shuffled_sites = random.sample(sites_to_shuffle, len(sites_to_shuffle))
shuffled_prefs = {}
for (site, shuffled_site) in zip(sites_to_shuffle, shuffled_sites):
    shuffled_prefs[shuffled_site] = prefs[site]
assert len(prefs.values()) == len(shuffled_prefs.values())

# Finally, write this dictionary of site-averaged preferences to a file
ordered_positions = [int(site) for site in prefs]
ordered_positions.sort()
ordered_positions = [str(site) for site in ordered_positions]
shuffled_prefs_file_name = 'simulated_alignments/shuffled_prefs.txt'
print "\nWriting the shuffled preferences to the file: %s"%shuffled_prefs_file_name
prefsutils.ConvertPrefsDictionaryToFile(shuffled_prefs, ordered_positions, shuffled_prefs_file_name)
```

```
Writing the shuffled preferences to the file: simulated_alignments/shuffled_prefs.txt
```

I will now conduct 100 simulations using the preferences shuffled between sites:

In [18]:

```
n_simulations = 100
for i in range(n_simulations):
    simulated_alignment_file_name = '%s/shuffled_prefs_simulated_alignment_sim_%s.fasta'%(simulated_alignment_dir_prefix, i) # Name of created alignment file
    sbatchfilename = '%s/run.sbatch_shuffled_prefs_sim_%s'%(simulated_alignment_dir_prefix, i)
    SimulateNewAlignment(shuffled_prefs_file_name, tree_file, modelparams, simulated_alignment_file_name, scalerate, sbatchfilename)
    i += 1
```

Next, I will conduct the second negative control of running simulations after averaging the preferences across all sites:

In [19]:

```
# Make a file of amino-acid preferences after averaging the preferences across sites. To do so, first read in the
# preferences, storing them in a dictionary:
prefs = prefsutils.ConvertPrefsFileToDictionary(prefs_file, CIs_present=False)

# Next, make a list of preferences for a given amino acid across sites
amino_acids = ['A', 'C', 'D', 'E', 'F', 'G', 'H', 'I', 'K', 'L', 'M', 'N', 'P', 'Q', 'R', 'S', 'T', 'V', 'W', 'Y', '*']
avg_pref_per_aa = {}
for aa in amino_acids:
    avg_pref_per_aa[aa] = numpy.mean([prefs[site][aa] for site in prefs])

# Make a new dictionary of preferences with the site-averaged preferences for all sites
site_avg_prefs = dict((site, avg_pref_per_aa) for site in prefs)

# Finally, write this dictionary of site-averaged preferences to a file
ordered_positions = [int(site) for site in prefs]
ordered_positions.sort()
ordered_positions = [str(site) for site in ordered_positions]
site_avg_prefs_file_name = 'simulated_alignments/site_averaged_prefs.txt'
print "\nWriting the site-averaged preferences to the file: %s"%site_avg_prefs_file_name
prefsutils.ConvertPrefsDictionaryToFile(site_avg_prefs, ordered_positions, site_avg_prefs_file_name)
```

```
Writing the site-averaged preferences to the file: simulated_alignments/site_averaged_prefs.txt
```

Conduct 100 simulations with the site-averaged preferences:

In [20]:

```
n_simulations = 100
for i in range(n_simulations):
    simulated_alignment_file_name = '%s/site_avg_prefs_simulated_alignment_sim_%s.fasta'%(simulated_alignment_dir_prefix, i) # Name of created alignment file
    sbatchfilename = '%s/run.sbatch_site_avg_prefs_sim_%s'%(simulated_alignment_dir_prefix, i)
    SimulateNewAlignment(site_avg_prefs_file_name, tree_file, modelparams, simulated_alignment_file_name, scalerate, sbatchfilename)
    i += 1
```

In [ ]:

```

```
