# Supplementary material for: Experimental Estimation of the Effects of All Amino-Acid Mutations to HIV’s Envelope Protein on Viral Replication in Cell Culture
Source: PLoS Pathog. 2016 Dec 13;12(12):e1006114. doi: 10.1371/journal.ppat.1006114 (PMC5189966; doi:10.1371/journal.ppat.1006114)
Supplement: S3 File — (ZIP) [file ppat.1006114.s013.zip › S3_File_notebooks/InferPreferences.html]

InferPreferences


# Use `dms_tools` to infer site-specific amino-acid preferences¶

## Imports¶

In [1]:

```
import os
import sys
sys.path.append('scripts/')
import prefsutils
from IPython.display import Image, display
import matplotlib
matplotlib.use("pdf")
matplotlib.rc('text', usetex=True)
import pylab
# % matplotlib inline
import scipy.stats
import numpy
from IPython.display import Image, display
```

## Global variables¶

In [2]:

```
# Replicates
replicates = [1, 2, 3, '3b']
```

Infer the preferences for each replicate:

In [3]:

```
print "The preferences will be inferred using:"
!dms_inferprefs -v

# A dictionary of preferences files (for passage 2 only) to create averaged preferences, keyed by replicate
prefs_for_averaging = {}

# Infer preferences
for replicate in replicates:
    print "\nAnalyzing replicate: %s" %replicate
    
    if replicate in [1, 2, 3]:
        
        # Specify output files
        prefs_p2_prefix = 'replicate-%s/prefs_p2'%replicate
        prefs_p2 = '%s.txt'%prefs_p2_prefix
        prefs_for_averaging[replicate] = prefs_p2
        
        # Command for inferring preferences
        cmd_inferprefs = ' '.join([
                            'dms_inferprefs',
                            'replicate-%s/mutDNA-%s/mutDNA-%s_edited_counts.txt'%(replicate, replicate, replicate), # npre
                            'replicate-%s/mutvirus-%s-p2/mutvirus-%s-p2_edited_counts.txt'%(replicate, replicate, replicate), # npost
                            prefs_p2, # outfile
                            '--errpre replicate-%s/DNA-%s/DNA-%s_edited_counts.txt'%(replicate, replicate, replicate), # errpre
                            '--errpost replicate-%s/virus-%s-p2/virus-%s-p2_edited_counts.txt'%(replicate, replicate, replicate), #errpost
                            '--ncpus -1'])
        print ("\nInferring preferences with the command:\n" + cmd_inferprefs)
        log = !$cmd_inferprefs
        
        if replicate != 3:
            continue
        
        # Specify output files for replicate 3 passage 1
        prefs_p1_prefix = 'replicate-%s/prefs_p1'%replicate
        prefs_p1 = '%s.txt'%prefs_p1_prefix

        # Command for inferring preferences
        cmd_inferprefs = ' '.join([
                            'dms_inferprefs',
                            'replicate-%s/mutDNA-%s/mutDNA-%s_edited_counts.txt'%(replicate, replicate, replicate), # npre
                            'replicate-%s/mutvirus-%s-p1/mutvirus-%s-p1_edited_counts.txt'%(replicate, replicate, replicate), # npost
                            prefs_p1, # outfile
                            '--errpre replicate-%s/DNA-%s/DNA-%s_edited_counts.txt'%(replicate, replicate, replicate), # errpre
                            '--errpost replicate-%s/virus-%s-p1/virus-%s-p1_edited_counts.txt'%(replicate, replicate, replicate), #errpost
                            '--ncpus -1'])
        print ("\nInferring preferences with the command:\n" + cmd_inferprefs)
        log = !$cmd_inferprefs
    
    # Replicates 3b-1 and 3b-2
    else:
        for subreplicate in ['3b-1', '3b-2']:
            
            # Specify output files
            prefs_p2_prefix = 'replicate-3b/prefs_p2-%s'%subreplicate
            prefs_p2 = '%s.txt'%prefs_p2_prefix
            prefs_for_averaging[subreplicate] = prefs_p2
            
            # Command for inferring preferences
            cmd_inferprefs = ' '.join([
                                'dms_inferprefs',
                                'replicate-3b/mutDNA-3b/mutDNA-3b_edited_counts.txt', # npre
                                'replicate-3b/mutvirus-%s-p2/mutvirus-%s-p2_edited_counts.txt'%(subreplicate, subreplicate), # npost
                                prefs_p2, # outfile
                                '--errpre replicate-3b/DNA-3b/DNA-3b_edited_counts.txt', # errpre
                                '--errpost replicate-3b/virus-%s-p2/virus-%s-p2_edited_counts.txt'%(subreplicate, subreplicate), #errpost
                                '--ncpus -1'])

            print ("\nInferring preferences with the command:\n" + cmd_inferprefs)
            log = !$cmd_inferprefs
```

```
The preferences will be inferred using:
dms_inferprefs 1.1.dev16

Analyzing replicate: 1

Inferring preferences with the command:
dms_inferprefs replicate-1/mutDNA-1/mutDNA-1_edited_counts.txt replicate-1/mutvirus-1-p2/mutvirus-1-p2_edited_counts.txt replicate-1/prefs_p2.txt --errpre replicate-1/DNA-1/DNA-1_edited_counts.txt --errpost replicate-1/virus-1-p2/virus-1-p2_edited_counts.txt --ncpus -1

Analyzing replicate: 2

Inferring preferences with the command:
dms_inferprefs replicate-2/mutDNA-2/mutDNA-2_edited_counts.txt replicate-2/mutvirus-2-p2/mutvirus-2-p2_edited_counts.txt replicate-2/prefs_p2.txt --errpre replicate-2/DNA-2/DNA-2_edited_counts.txt --errpost replicate-2/virus-2-p2/virus-2-p2_edited_counts.txt --ncpus -1

Analyzing replicate: 3

Inferring preferences with the command:
dms_inferprefs replicate-3/mutDNA-3/mutDNA-3_edited_counts.txt replicate-3/mutvirus-3-p2/mutvirus-3-p2_edited_counts.txt replicate-3/prefs_p2.txt --errpre replicate-3/DNA-3/DNA-3_edited_counts.txt --errpost replicate-3/virus-3-p2/virus-3-p2_edited_counts.txt --ncpus -1

Inferring preferences with the command:
dms_inferprefs replicate-3/mutDNA-3/mutDNA-3_edited_counts.txt replicate-3/mutvirus-3-p1/mutvirus-3-p1_edited_counts.txt replicate-3/prefs_p1.txt --errpre replicate-3/DNA-3/DNA-3_edited_counts.txt --errpost replicate-3/virus-3-p1/virus-3-p1_edited_counts.txt --ncpus -1

Analyzing replicate: 3b

Inferring preferences with the command:
dms_inferprefs replicate-3b/mutDNA-3b/mutDNA-3b_edited_counts.txt replicate-3b/mutvirus-3b-1-p2/mutvirus-3b-1-p2_edited_counts.txt replicate-3b/prefs_p2-3b-1.txt --errpre replicate-3b/DNA-3b/DNA-3b_edited_counts.txt --errpost replicate-3b/virus-3b-1-p2/virus-3b-1-p2_edited_counts.txt --ncpus -1

Inferring preferences with the command:
dms_inferprefs replicate-3b/mutDNA-3b/mutDNA-3b_edited_counts.txt replicate-3b/mutvirus-3b-2-p2/mutvirus-3b-2-p2_edited_counts.txt replicate-3b/prefs_p2-3b-2.txt --errpre replicate-3b/DNA-3b/DNA-3b_edited_counts.txt --errpost replicate-3b/virus-3b-2-p2/virus-3b-2-p2_edited_counts.txt --ncpus -1
```

## Average the preferences between replicates using `dms_merge`¶

First, I will average the passage 2 preferences for replicates 3b-1 and 3b-2:

In [4]:

```
print "Preferences will be averaged using:"
!dms_merge -v

avg_prefs_dir_prefix = 'averaged_preferences'
if not os.path.isdir('%s/'%avg_prefs_dir_prefix):
    os.makedirs('%s/'%avg_prefs_dir_prefix)

averaged_prefs_3b = '%s/avg_3b_prefs_p2.txt'%avg_prefs_dir_prefix
prefs_for_averaging_string = ' '.join([prefs_for_averaging['3b-1'], prefs_for_averaging['3b-2']])

cmd_merge = ' '.join([
                'dms_merge',
                averaged_prefs_3b, # outfile
                'average', # command
                prefs_for_averaging_string # infiles
                ])

print ("The preferences will be averaged using the command:\n" + cmd_merge)
log = !$cmd_merge
```

```
Preferences will be averaged using:
dms_merge 1.1.dev16
The preferences will be averaged using the command:
dms_merge averaged_preferences/avg_3b_prefs_p2.txt average replicate-3b/prefs_p2-3b-1.txt replicate-3b/prefs_p2-3b-2.txt
```

Next, I will average the passage 2 preferences for replicates 1-3 and the averaged 3b preferences:

In [5]:

```
print "Preferences will be averaged using:"
!dms_merge -v

avg_prefs_dir_prefix = 'averaged_preferences'
if not os.path.isdir('%s/'%avg_prefs_dir_prefix):
    os.makedirs('%s/'%avg_prefs_dir_prefix)

averaged_prefs = '%s/avg_prefs_p2.txt'%avg_prefs_dir_prefix
prefs_for_averaging_string = ' '.join([prefs_for_averaging[rep] for rep in [1, 2, 3]] + [averaged_prefs_3b])

cmd_merge = ' '.join([
                'dms_merge',
                averaged_prefs, # outfile
                'average', # command
                prefs_for_averaging_string # infiles
                ])

print ("The preferences will be averaged using the command:\n" + cmd_merge)
log = !$cmd_merge
```

```
Preferences will be averaged using:
dms_merge 1.1.dev16
The preferences will be averaged using the command:
dms_merge averaged_preferences/avg_prefs_p2.txt average replicate-1/prefs_p2.txt replicate-2/prefs_p2.txt replicate-3/prefs_p2.txt averaged_preferences/avg_3b_prefs_p2.txt
```

## Convert the preferences to HXB2 numbering¶

I will change the preferences to follow HXB2 numbering. The renumbering scheme is shown in the below renumbering file for `dms_editsites`. Based on BLAST and CLUSTAL O(1.2.1) alignments, there appears to be a 5-residue insertion in LAI relative to HXB2. The precise location of this insertion is not clear because it involves a repetitive segment of LAI. To align the two for renumbering, I decided to treat positions 1-142 as homologous in both sequences. Then, I decided to introduce a gap, with LAI positions 143-147 having no homologous sites in HXB2; instead, numbering them as ('142a', '142b', ..., '142e') . Then, I decided to treat LAI sites 148-end and HXB2 sites 143-end as homologous. The location of the indel in my alignment between LAI and HXB2 Envs is:

```
DTNTNS-----SSGR (HXB2 amino acids 137-146)
ATNTNSSNTNSSSGE (LAI amino acids 137-151)
```

First, I will write an input file for `dms_editsites` specifying the renumbering scheme:

In [7]:

```
LAI_to_HXB2_renumbering_file_name = 'LAI_to_HXB2_renumbering_file.txt'
LAI_to_HXB2_renumbering_file = open(LAI_to_HXB2_renumbering_file_name, 'w')
LAI_to_HXB2_renumbering_file.write('# original_LAI_site new_HXB2_site\n')
for LAI_site in range(31, 708):
    HXB2_site = prefsutils.ConvertNumberingLAItoHXBII(str(LAI_site))
    LAI_to_HXB2_renumbering_file.write("%s %s\n"%(LAI_site, HXB2_site))
LAI_to_HXB2_renumbering_file.close()
```

In [8]:

```
def ChangePrefsToHxb2NumAndMakeLogoPlots(prefs, prefs_nhxb2, prefs_nhxb2_logo_with_stopcodons, prefs_nhxb2_logo):
    """
    This function takes the name of a .txt file of LAI Env preferences as input (*prefs*) and does two things. First,
    it converts the preferences to HXB2 numbering. Second, it creates logo plots with and without stop codons.
    """
    # Change numbering of preferences file to HXBII numbering
    cmd_nhxb2 = ' '.join([
                    'dms_editsites',
                    prefs, # input file
                    prefs_nhxb2, # output file
                    'renumber', # command
                    'LAI_to_HXB2_renumbering_file.txt']) # renumbering file
    print ("\nConverting the preferences to HXB2 numbering with the command:\n" + cmd_nhxb2)
    log = !$cmd_nhxb2
    
    # Make a logo plot with stop codons
    cmd_logoplotstops = ' '.join([
                            'dms_logoplot',
                            prefs_nhxb2,
                            prefs_nhxb2_logo_with_stopcodons,
                            '--nperline 76'])
    print ("\nMaking a logo plot with stop codons with the command:\n" + cmd_logoplotstops)
    log = !$cmd_logoplotstops
    
    # Make a logo plot without stop codons
    cmd_logoplot = ' '.join([
                        'dms_logoplot',
                        prefs_nhxb2,
                        prefs_nhxb2_logo,
                        '--nperline 76',
                        '--excludestop'])
    print ("\nMaking a logo plot without stop codons with the command:\n" + cmd_logoplot)
    log = !$cmd_logoplot
    
    return None
```

Next, I will use the above renumbering file to first change each preference file to HXB2 numbering and then create sequence-logo plots, both with and without stop codons:

In [9]:

```
print "\nThe prefs files will be renumbered using:"
!dms_editsites -v

print "\nLogo plots will be made using:"
!dms_logoplot -v

for replicate in replicates:
    if replicate in [1, 2, 3]:
        print "\nProcessing replicate: %s" %replicate
        # Specify output files
        prefs_p2_prefix = 'replicate-%s/prefs_p2'%replicate
        prefs_p2 = '%s.txt'%prefs_p2_prefix
        prefs_p2_nhxb2 = '%s_nhxb2.txt'%prefs_p2_prefix
        prefs_p2_nhxb2_logo_with_stopcodons = '%s_with_stopcodons_nhxb2.pdf'%prefs_p2_prefix
        prefs_p2_nhxb2_logo = '%s_nhxb2.pdf'%prefs_p2_prefix
        # Renumber the preferences and make logo plots
        ChangePrefsToHxb2NumAndMakeLogoPlots(prefs_p2, prefs_p2_nhxb2, prefs_p2_nhxb2_logo_with_stopcodons, prefs_p2_nhxb2_logo)

        if replicate != 3:
            continue
        # Specify output files for replicate 3 passage 1
        prefs_p1_prefix = 'replicate-%s/prefs_p1'%replicate
        prefs_p1 = '%s.txt'%prefs_p1_prefix
        prefs_p1_nhxb2 = '%s_nhxb2.txt'%prefs_p1_prefix
        prefs_p1_nhxb2_logo_with_stopcodons = '%s_with_stopcodons_nhxb2.pdf'%prefs_p1_prefix
        prefs_p1_nhxb2_logo = '%s_nhxb2.pdf'%prefs_p1_prefix
        # Renumber the preferences and make logo plots
        ChangePrefsToHxb2NumAndMakeLogoPlots(prefs_p1, prefs_p1_nhxb2, prefs_p1_nhxb2_logo_with_stopcodons, prefs_p1_nhxb2_logo)

    else:
        for subreplicate in ['3b-1', '3b-2']:
            # Specify output files
            prefs_p2_prefix = 'replicate-3b/prefs_p2-%s'%subreplicate
            prefs_p2 = '%s.txt'%prefs_p2_prefix
            prefs_p2_nhxb2 = '%s_nhxb2.txt'%prefs_p2_prefix
            prefs_p2_nhxb2_logo_with_stopcodons = '%s_with_stopcodons_nhxb2.pdf'%prefs_p2_prefix
            prefs_p2_nhxb2_logo = '%s_nhxb2.pdf'%prefs_p2_prefix
            # Renumber the preferences and make logo plots
            ChangePrefsToHxb2NumAndMakeLogoPlots(prefs_p2, prefs_p2_nhxb2, prefs_p2_nhxb2_logo_with_stopcodons, prefs_p2_nhxb2_logo)

print "\nProcessing the averaged preferences"
averaged_prefs_prefix = '%s/avg_prefs_p2'%avg_prefs_dir_prefix
averaged_prefs = '%s.txt'%averaged_prefs_prefix
averaged_prefs_nhxb2 = '%s_nhxb2.txt'%averaged_prefs_prefix
averaged_prefs_p2_nhxb2_logo_with_stopcodons = '%s_with_stopcodons_nhxb2.pdf'%averaged_prefs_prefix
averaged_prefs_p2_nhxb2_logo = '%s_nhxb2.pdf'%averaged_prefs_prefix
ChangePrefsToHxb2NumAndMakeLogoPlots(averaged_prefs, averaged_prefs_nhxb2, averaged_prefs_p2_nhxb2_logo_with_stopcodons, averaged_prefs_p2_nhxb2_logo)
```

```
The prefs files will be renumbered using:
dms_editsites 1.1.dev16

Logo plots will be made using:
dms_logoplot 1.1.dev16

Processing replicate: 1

Converting the preferences to HXB2 numbering with the command:
dms_editsites replicate-1/prefs_p2.txt replicate-1/prefs_p2_nhxb2.txt renumber LAI_to_HXB2_renumbering_file.txt

Making a logo plot with stop codons with the command:
dms_logoplot replicate-1/prefs_p2_nhxb2.txt replicate-1/prefs_p2_with_stopcodons_nhxb2.pdf --nperline 76

Making a logo plot without stop codons with the command:
dms_logoplot replicate-1/prefs_p2_nhxb2.txt replicate-1/prefs_p2_nhxb2.pdf --nperline 76 --excludestop

Processing replicate: 2

Converting the preferences to HXB2 numbering with the command:
dms_editsites replicate-2/prefs_p2.txt replicate-2/prefs_p2_nhxb2.txt renumber LAI_to_HXB2_renumbering_file.txt

Making a logo plot with stop codons with the command:
dms_logoplot replicate-2/prefs_p2_nhxb2.txt replicate-2/prefs_p2_with_stopcodons_nhxb2.pdf --nperline 76

Making a logo plot without stop codons with the command:
dms_logoplot replicate-2/prefs_p2_nhxb2.txt replicate-2/prefs_p2_nhxb2.pdf --nperline 76 --excludestop

Processing replicate: 3

Converting the preferences to HXB2 numbering with the command:
dms_editsites replicate-3/prefs_p2.txt replicate-3/prefs_p2_nhxb2.txt renumber LAI_to_HXB2_renumbering_file.txt

Making a logo plot with stop codons with the command:
dms_logoplot replicate-3/prefs_p2_nhxb2.txt replicate-3/prefs_p2_with_stopcodons_nhxb2.pdf --nperline 76

Making a logo plot without stop codons with the command:
dms_logoplot replicate-3/prefs_p2_nhxb2.txt replicate-3/prefs_p2_nhxb2.pdf --nperline 76 --excludestop

Converting the preferences to HXB2 numbering with the command:
dms_editsites replicate-3/prefs_p1.txt replicate-3/prefs_p1_nhxb2.txt renumber LAI_to_HXB2_renumbering_file.txt

Making a logo plot with stop codons with the command:
dms_logoplot replicate-3/prefs_p1_nhxb2.txt replicate-3/prefs_p1_with_stopcodons_nhxb2.pdf --nperline 76

Making a logo plot without stop codons with the command:
dms_logoplot replicate-3/prefs_p1_nhxb2.txt replicate-3/prefs_p1_nhxb2.pdf --nperline 76 --excludestop

Converting the preferences to HXB2 numbering with the command:
dms_editsites replicate-3b/prefs_p2-3b-1.txt replicate-3b/prefs_p2-3b-1_nhxb2.txt renumber LAI_to_HXB2_renumbering_file.txt

Making a logo plot with stop codons with the command:
dms_logoplot replicate-3b/prefs_p2-3b-1_nhxb2.txt replicate-3b/prefs_p2-3b-1_with_stopcodons_nhxb2.pdf --nperline 76

Making a logo plot without stop codons with the command:
dms_logoplot replicate-3b/prefs_p2-3b-1_nhxb2.txt replicate-3b/prefs_p2-3b-1_nhxb2.pdf --nperline 76 --excludestop

Converting the preferences to HXB2 numbering with the command:
dms_editsites replicate-3b/prefs_p2-3b-2.txt replicate-3b/prefs_p2-3b-2_nhxb2.txt renumber LAI_to_HXB2_renumbering_file.txt

Making a logo plot with stop codons with the command:
dms_logoplot replicate-3b/prefs_p2-3b-2_nhxb2.txt replicate-3b/prefs_p2-3b-2_with_stopcodons_nhxb2.pdf --nperline 76

Making a logo plot without stop codons with the command:
dms_logoplot replicate-3b/prefs_p2-3b-2_nhxb2.txt replicate-3b/prefs_p2-3b-2_nhxb2.pdf --nperline 76 --excludestop

Processing the averaged preferences

Converting the preferences to HXB2 numbering with the command:
dms_editsites averaged_preferences/avg_prefs_p2.txt averaged_preferences/avg_prefs_p2_nhxb2.txt renumber LAI_to_HXB2_renumbering_file.txt

Making a logo plot with stop codons with the command:
dms_logoplot averaged_preferences/avg_prefs_p2_nhxb2.txt averaged_preferences/avg_prefs_p2_with_stopcodons_nhxb2.pdf --nperline 76

Making a logo plot without stop codons with the command:
dms_logoplot averaged_preferences/avg_prefs_p2_nhxb2.txt averaged_preferences/avg_prefs_p2_nhxb2.pdf --nperline 76 --excludestop
```

In [ ]:

```

```
