# Supplementary material for: Experimental Estimation of the Effects of All Amino-Acid Mutations to HIV’s Envelope Protein on Viral Replication in Cell Culture
Source: PLoS Pathog. 2016 Dec 13;12(12):e1006114. doi: 10.1371/journal.ppat.1006114 (PMC5189966; doi:10.1371/journal.ppat.1006114)
Supplement: S3 File — (ZIP) [file ppat.1006114.s013.zip › S3_File_notebooks/Sanger_sequencing_of_libraries/AnalyzeResultsOfSangerSequencingTheLibraries.html]

AnalyzeResultsOfSangerSequencingTheLibraries


# Analyze the results of Sangers sequencing the libraries¶

As an initial assessment of the diversity of mutations in our plasmid libraries, we Sanger sequencing 26 clones sampled roughly evenly from the three replicate libraries.

I analyzed the sequencing data using a Python script written by the Bloom lab (https://github.com/jbloomlab/SangerMutantLibraryAnalysis). This link provides detailed documentation and examples of how to use this script. In this IPython notebook, I write input files for this script, including a FASTA file of LAI env and a file listing the mutations observed in the Sanger sequencing data. I also write to a file the specific version of the python script I used to analyze the data. Finally, I execute the analysis through the command line using `%%bash` to execute command-line arguments in the terminal. The output includes plots that summarize the distribution of mutations in the library.

## Write input files and analysis script¶

Input file giving LAI env's nucleotide sequence from the first sequence through the region of env I mutagenized in the deep mutational scanning experiment:

In [1]:

```
%%writefile LAI-Env.fasta
>LAI Env gene truncated at the end of the region I am mutagenizing
atgagagtgaaggagaaatatcagcacttgtggagatgggggtggaaatggggcaccatgctccttgggatattgatgatctgtagtgctACAGAAAAATTGTGGGTCACAGTCTATTATGGGGTACCTGTGTGGAAGGAAGCAACCACCACTCTATTTTGTGCATCAGATGCTAAAGCATATGATACAGAGGTACATAATGTTTGGGCCACACATGCCTGTGTACCCACAGACCCCAACCCACAAGAAGTAGTATTGGTAAATGTGACAGAAAATTTTAACATGTGGAAAAATGACATGGTAGAACAGATGCATGAGGATATAATCAGTTTATGGGATCAAAGCCTAAAGCCATGTGTAAAATTAACCCCACTCTGTGTTAGTTTAAAGTGCACTGATTTGGGGAATGCTACTAATACCAATAGTAGTAATACCAATAGTAGTAGCGGGGAAATGATGATGGAGAAAGGAGAGATAAAAAACTGCTCTTTCAATATCAGCACAAGCATAAGAGGTAAGGTGCAGAAAGAATATGCATTTTTTTATAAACTTGATATAATACCAATAGATAATGATACTACCAGCTATACGTTGACAAGTTGTAACACCTCAGTCATTACACAGGCCTGTCCAAAGGTATCCTTTGAGCCAATTCCCATACATTATTGTGCCCCGGCTGGTTTTGCGATTCTAAAATGTAATAATAAGACGTTCAATGGAACAGGACCATGTACAAATGTCAGCACAGTACAATGTACACATGGAATTAGGCCAGTAGTATCAACTCAACTGCTGTTGAATGGCAGTCTAGCAGAAGAAGAGGTAGTAATTAGATCTGCCAATTTCACAGACAATGCTAAAACCATAATAGTACAGCTGAACCAATCTGTAGAAATTAATTGTACAAGACCCAACAACAATACAAGAAAAAGTATCCGTATCCAGAGGGGACCAGGGAGAGCATTTGTTACAATAGGAAAAATAGGAAATATGAGACAAGCACATTGTAACATTAGTAGAGCAAAATGGAATGCCACTTTAAAACAGATAGCTAGCAAATTAAGAGAACAATTTGGAAATAATAAAACAATAATCTTTAAGCAATCCTCAGGAGGGGACCCAGAAATTGTAACGCACAGTTTTAATTGTGGAGGGGAATTTTTCTACTGTAATTCAACACAACTGTTTAATAGTACTTGGTTTAATAGTACTTGGAGTACTGAAGGGTCAAATAACACTGAAGGAAGTGACACAATCACACTCCCATGCAGAATAAAACAATTTATAAACATGTGGCAGGAAGTAGGAAAAGCAATGTATGCCCCTCCCATCAGCGGACAAATTAGATGTTCATCAAATATTACAGGGCTGCTATTAACAAGAGATGGTGGTAATAACAACAATGGGTCCGAGATCTTCAGACCTGGAGGAGGAGATATGAGGGACAATTGGAGAAGTGAATTATATAAATATAAAGTAGTAAAAATTGAACCATTAGGAGTAGCACCCACCAAGGCAAAGAGAAGAGTGGTGCAGAGAGAAAAAAGAGCAGTGGGAATAGGAGCTTTGTTCCTTGGGTTCTTGGGAGCAGCAGGAAGCACTATGGGCGCAGCGTCAATGACGCTGACGGTACAGGCCAGACAATTATTGTCTGGTATAGTGCAGCAGCAGAACAATTTGCTGAGGGCTATTGAGGCGCAACAGCATCTGTTGCAACTCACAGTCTGGGGCATCAAGCAGCTCCAGGCAAGAATCCTGGCTGTGGAAAGATACCTAAAGGATCAACAGCTCCTGGGGATTTGGGGTTGCTCTGGAAAACTCATTTGCACCACTGCTGTGCCTTGGAATGCTAGTTGGAGTAATAAATCTCTGGAACAGATTTGGAATAACATGACCTGGATGGAGTGGGACAGAGAAATTAACAATTACACAAGCTTAATACATTCCTTAATTGAAGAATCGCAAAACCAGCAAGAAAAGAATGAACAAGAATTATTGGAATTAGATAAATGGGCAAGTTTGTGGAATTGGTTTAACATAACAAATTGGCTGTGGTATATAAAAATATTCATAATGATAGTAGGAGGCTTGGTAGGTTTAAGAATAGTTTTTGCTGTACTT
```

```
Overwriting LAI-Env.fasta
```

Input file documenting the mutations I observed from Sanger sequencing:

In [2]:

```
%%writefile mut_list.txt

# Sequencing from 4-1-14, 4-4-14, and 4-7-14 for indicated clones
WT1round2-1: CTA808GTC, GCA1609GGT
WT1round2-2: None
WT1round2-3: G724T, TGT730GGG
WT1round2-4: C209T
WT1round2-5: AAG136CGG, GAA271ATG
WT1round2-6: delA293, GAT574AAA, TCA781GCA, AGT805GGG
WT1round2-7: None
WT1round2-10: TTC1417TAT, insA1546

WT2round2-1: A229C
WT2round2-2: GGG1405AAC
WT2round2-3: None
WT2round2-5: A1886G
WT2round2-6: A723G, T1239C, G1837A
WT2round2-7: GGG1153TAA
WT2round2-8: GGG1225ACC
WT2round2-9: AAT571CTT, T1350G, T1810C
WT2round2-10: None

WT3.2round2-1: ATA868TAA, CAA1783GTT
WT3.2round2-2: CAA340CTG, delG344
WT3.2round2-3: GAG1411TCA, GCG1696TGG
WT3.2round2-4: AGT439TTA, C952A
WT3.2round2-5: None
WT3.2round2-6: TTT1282ATA, AAG1519TAC, CTG1756TTC
WT3.2round2-7: TGT160GGA
WT3.2round2-9: GAA1156CCG, CTG1756AAA
WT3.2round2-10: ATG460TTT, TGT754TAG
```

```
Overwriting mut_list.txt
```

Python script for analyzing the Sanger sequencing data:

In [3]:

```
%%writefile analyze_library.py

"""Script to analyze mutations to a coding sequence.

Written by Jesse Bloom, 2013

Edited by Hugh Haddox, October-16-2015"""

import re
import os
import time
import math
import random
import matplotlib
matplotlib.use('pdf') # use the PDF backend
matplotlib.rc('legend', fontsize=12)
import pylab
import scipy.stats


def TranslateCodon(codon):
    """Returns one-letter amino acid code for *codon*.

    *codon* is a 3-letter string giving a valid codon."""
    genetic_code = {'TTT':'F', 'TTC':'F', 'TTA':'L', 'TTG':'L', 'CTT':'L', 'CTC':'L',
        'CTA':'L', 'CTG':'L', 'ATT':'I', 'ATC':'I', 'ATA':'I', 'ATG':'M', 'GTT':'V',
        'GTC':'V', 'GTA':'V', 'GTG':'V', 'TCT':'S', 'TCC':'S', 'TCA':'S',
        'TCG':'S', 'CCT':'P', 'CCC':'P', 'CCA':'P', 'CCG':'P', 'ACT':'T',
        'ACC':'T', 'ACA':'T', 'ACG':'T', 'GCT':'A', 'GCC':'A', 'GCA':'A',
        'GCG':'A', 'TAT':'Y', 'TAC':'Y', 'TAA':'*', 'TAG':'*',
        'CAT':'H', 'CAC':'H', 'CAA':'Q', 'CAG':'Q', 'AAT':'N', 'AAC':'N',
        'AAA':'K', 'AAG':'K', 'GAT':'D', 'GAC':'D', 'GAA':'E', 'GAG':'E',
        'TGT':'C', 'TGC':'C', 'TGA':'*', 'TGG':'W', 'CGT':'R',
        'CGC':'R', 'CGA':'R', 'CGG':'R', 'AGT':'S', 'AGC':'S', 'AGA':'R',
        'AGG':'R', 'GGT':'G', 'GGC':'G', 'GGA':'G', 'GGG':'G'}
    return genetic_code[codon.upper()]


def PlotMutationClustering(mutation_nums_by_clone, ncodons, plotfile, title, mutstart, nsimulations=1000):
    """Plots clustering of mutations versus null expectation of no clustering.

    This function addresses the question of whether clones with multiple
    mutations tend to have those mutations clustered in primary sequence.
    Clones with <2 mutations are not considered. For every clone with >= 2 
    mutations, records the distance in primary sequence between each pair
    of mutations. Then performs nsimulations simulations of placing this
    number of mutations at random on a gene, and records the distance in
    primary sequence between each pair in the simulated sequences. Plots
    the actual distributions of primary sequence distances versus
    the simulated distribution.

    mutation_nums_by_clone -> list, with an entry for each clone. Each of
        these entries is itself a list. The entries in these sublists are
        numbers (1, 2, 3, ...) of the codon positions mutated in that clone.
    ncodons -> integer number of codons in the gene.
    plotfile -> name of the plot file we create.
    title -> string giving the plot title.
    nsimulations -> number of simulations for each clone. Is 1000 by default.
    mutstart -> specifies the first codon in the mutated segment of the gene
        (integer). This variable will be used to truncate the gene to the
        appropriate length for simulating the random distribution of distances
        between mutations.
    """
    actual_distances = dict([(i, 0) for i in range(1, ncodons - mutstart + 1)])
    simulated_distances = dict([(i, 0) for i in range(1, ncodons - mutstart + 1)])
    codons = [i for i in range(1, ncodons - mutstart + 2)]
    nactual = nsimulated = 0
    for mutpositions in mutation_nums_by_clone:
        nmuts = len(mutpositions)
        if nmuts < 2:
            continue
        for i in range(nmuts):
            for j in range(i + 1, nmuts):
                d = abs(mutpositions[i] - mutpositions[j])
                actual_distances[d] += 1
                nactual += 1
        for isimulate in range(nsimulations):
            simulpositions = random.sample(codons, nmuts)
            for i in range(nmuts):
                for j in range(i + 1, nmuts):
                    d = abs(simulpositions[i] - simulpositions[j])
                    simulated_distances[d] += 1
                    nsimulated += 1
    actual_cumul = []
    simulated_cumul = []
    actual_tot = simul_tot = 0.0
    for d in range(1, ncodons - mutstart + 1):
        actual_tot += actual_distances[d] / float(nactual)
        simul_tot += simulated_distances[d] / float(nsimulated)
        actual_cumul.append(actual_tot)
        simulated_cumul.append(simul_tot)
    pylab.figure(figsize=(4.5, 2.25))
    (lmargin, rmargin, bmargin, tmargin) = (0.13, 0.01, 0.21, 0.07)
    pylab.axes([lmargin, bmargin, 1.0 - lmargin - rmargin, 1.0 - bmargin - tmargin])
    barwidth = 0.7
    xs = [x for x in range(1, ncodons - mutstart + 1)]
    assert len(xs) == len(actual_cumul) == len(simulated_cumul)
    pred = pylab.plot(xs, simulated_cumul, 'b--')
    actual = pylab.plot(xs, actual_cumul, 'r-')
    pylab.gca().set_xlim([0, ncodons])
    pylab.gca().set_ylim([0, 1])
    pylab.gca().xaxis.set_major_locator(matplotlib.ticker.MaxNLocator(6))
    pylab.gca().yaxis.set_major_locator(matplotlib.ticker.FixedLocator([0, 0.5, 1]))
    pylab.xlabel('distance between pairs of mutations')
    pylab.ylabel('cumulative fraction')
    pylab.legend((actual[0], pred[0]), ('actual', 'expected'), loc='lower right', numpoints=1, handlelength=2, ncol=2, borderaxespad=0.4, handletextpad=0.4, columnspacing=1.1)
    pylab.title(title, fontsize=12)
    pylab.savefig(plotfile)
    time.sleep(0.5)
    pylab.show()


def PlotCodonMutNTComposition(allmutations, plotfile, title):
    """Plots nucleotide composition of mutant codons and mtuated.

    For each site containing a mutated codon, looks at the nucleotide
    composition of all three sites at the original and new codon. Plots the 
    overall frequency of each nucleotide at these sites.

    allmutations -> list of all mutations as tuples (wtcodon, r, mutcodon)
    plotfile -> name of the plot file we create.
    title -> string giving the plot title.
    """
    nts = ['A', 'T', 'C', 'G']
    wtntcounts = dict([(nt, 0) for nt in nts])
    mutntcounts = dict([(nt, 0) for nt in nts])
    ntot = float(3 * len(allmutations))
    for (wtcodon, r, mutcodon) in allmutations:
        for nt in wtcodon:
            wtntcounts[nt] += 1 / ntot
        for nt in mutcodon:
            mutntcounts[nt] += 1 / ntot
    pylab.figure(figsize=(3.5, 2.25))
    (lmargin, rmargin, bmargin, tmargin) = (0.16, 0.01, 0.21, 0.07)
    pylab.axes([lmargin, bmargin, 1.0 - lmargin - rmargin, 1.0 - bmargin - tmargin])
    barwidth = 0.35
    xs = [i for i in range(len(nts))]
    nwt = pylab.bar([x - barwidth for x in xs], [wtntcounts[nt] for nt in nts], width=barwidth, color='blue')
    nmut = pylab.bar([x for x in xs], [mutntcounts[nt] for nt in nts], width=barwidth, color='red')
    #pred = pylab.plot(xs, nexpected, 'rx', markersize=6, mew=3)
    pylab.gca().set_xlim([-0.5, 3.5])
    pylab.gca().set_ylim([0, max(wtntcounts.values() + mutntcounts.values()) * 1.35])
    #pylab.gca().xaxis.set_major_locator(matplotlib.ticker.MaxNLocator(4))
    pylab.gca().yaxis.set_major_locator(matplotlib.ticker.MaxNLocator(5))
    pylab.xlabel('nucleotide')
    pylab.ylabel('codon composition')
    pylab.legend((nwt[0], nmut[0]), ('parent', 'mutant'), loc='upper center', numpoints=1, handlelength=1, ncol=2, borderaxespad=0.01, columnspacing=1.1, handletextpad=0.4)
    pylab.title(title, fontsize=12)
    pylab.xticks(pylab.arange(0, 4, 1), tuple(nts))
    pylab.savefig(plotfile)
    time.sleep(0.5)
    pylab.show()


def PlotNCodonMuts(allmutations, plotfile, title):
    """Plots number of nucleotide changes per codon mutation.

    allmutations -> list of all mutations as tuples (wtcodon, r, mutcodon)
    plotfile -> name of the plot file we create.
    title -> string giving the plot title.
    """
    pylab.figure(figsize=(3.5, 2.25))
    (lmargin, rmargin, bmargin, tmargin) = (0.16, 0.01, 0.21, 0.07)
    pylab.axes([lmargin, bmargin, 1.0 - lmargin - rmargin, 1.0 - bmargin - tmargin])
    nchanges = {1:0, 2:0, 3:0}
    nmuts = len(allmutations)
    for (wtcodon, r, mutcodon) in allmutations:
        assert 3 == len(wtcodon) == len(mutcodon)
        diffs = len([i for i in range(3) if wtcodon[i] != mutcodon[i]])
        nchanges[diffs] += 1
    barwidth = 0.6
    xs = [1, 2, 3]
    nactual = [nchanges[x] for x in xs]
    nexpected = [nmuts * 9. / 63., nmuts * 27. / 63., nmuts * 27. / 63.]
    bar = pylab.bar([x - barwidth / 2.0 for x in xs], nactual, width=barwidth)
    pred = pylab.plot(xs, nexpected, 'rx', markersize=6, mew=3)
    pylab.gca().set_xlim([0.5, 3.5])
    pylab.gca().set_ylim([0, max(nactual + nexpected) * 1.1])
    pylab.gca().xaxis.set_major_locator(matplotlib.ticker.MaxNLocator(4))
    pylab.gca().yaxis.set_major_locator(matplotlib.ticker.MaxNLocator(5))
    pylab.xlabel('nucleotide changes in codon')
    pylab.ylabel('number of mutations')
    pylab.legend((bar[0], pred[0]), ('actual', 'expected'), loc='upper left', numpoints=1, handlelength=0.9, borderaxespad=0, handletextpad=0.4)
    pylab.title(title, fontsize=12)
    pylab.savefig(plotfile)
    time.sleep(0.5)
    pylab.show()


def PlotNMutDist(nmutations, plotfile, title):
    """Plots number of mutations per gene versus Poisson distribution.

    nmutations -> list of number of mutations per gene.
    plotfile -> name of the plot file that we create.
    title -> string giving the plot title.
    """
    pylab.figure(figsize=(3.5, 2.25))
    (lmargin, rmargin, bmargin, tmargin) = (0.20, 0.01, 0.21, 0.07)
    pylab.axes([lmargin, bmargin, 1.0 - lmargin - rmargin, 1.0 - bmargin - tmargin])
    nseqs = len(nmutations)
    mavg = scipy.mean(nmutations)
    barwidth = 0.8
    xmax = max(nmutations) + 2
    nmuts = [i for i in range(xmax + 1)]
    nactual = [nmutations.count(n) for n in nmuts]
    npoisson = [nseqs * math.exp(-mavg) * mavg**n / math.factorial(n) for n in nmuts]
    bar = pylab.bar([n - barwidth / 2.0 for n in nmuts], nactual, width=barwidth)
    pred = pylab.plot(nmuts, npoisson, 'rx', markersize=6, mew=3)
    pylab.gca().set_xlim([-0.5, xmax + 0.5])
    pylab.gca().set_ylim([0, max(nactual) + 1.5])
    pylab.gca().xaxis.set_major_locator(matplotlib.ticker.MaxNLocator(4))
    pylab.gca().yaxis.set_major_locator(matplotlib.ticker.MaxNLocator(5))
    pylab.xlabel('number of mutated codons')
    pylab.ylabel('number of clones')
    pylab.legend((bar[0], pred[0]), ('actual', 'Poisson'), loc='upper right', numpoints=1, handlelength=1.2, ncol=1, borderaxespad=0)
    #
    # Kolmogorov-Smirnov test
    def F(n):
        return scipy.stats.poisson.cdf(n, mavg)
    (d, p) = scipy.stats.kstest(scipy.array(nmutations), F)
#    pylab.text(0.98, 0.6, 
#        'P = %.3f for Kolmogorov-Smirnov test of\n' % p +\
#        'whether actual distribution would differ\n' +\
#        'from Poisson at least this much by chance.',
#        horizontalalignment='right',
#        verticalalignment='bottom',
#        transform=pylab.gca().transAxes, 
#        fontsize=11)
    pylab.title(title, fontsize=12)
    pylab.savefig(plotfile)
    time.sleep(0.5)
    pylab.show()


def PlotGeneMutDist(genelength, sub_nums, indel_nums, plotfile, cumulplotfile, title, mutstart):
    """Plots mutation distribution along a gene.

    genelength -> length of gene (integer)
    sub_nums -> list of positions of substitutions (1, 2, ... numbering)
    indel_nums -> list of positions of indels (1, 2, ... numbering)
    plotfile -> string giving plot file name for plot with lines at each
        site.
    cumulplotfile -> string giving name of cumulative distribution plot file.
    title -> string giving plot title
    mutstart -> specifies the first codon in the mutated portion of the gene (integer).
        The cumulative and uniform distributions will begin at this position.
    """
    nsubs = len(sub_nums)
    if not nsubs:
        raise ValueError("empty sub_nums")
    xs = [i for i in range(mutstart, genelength + 1)]
    subs = dict([(x, 0) for x in xs])
    indels = dict([(x, 0) for x in xs])
    for x in sub_nums:
        subs[x] += 1
    for x in indel_nums:
        indels[x] += 1
    subs = [subs[x] for x in xs]
    indels = [-indels[x] for x in xs]
    barwidth = 1.0
    xlefts = [x - barwidth / 2. for x in xs]
    pylab.figure(figsize=(5.5, 2.5))
    (lmargin, rmargin, bmargin, tmargin) = (0.1, 0.03, 0.2, 0.09)
    pylab.axes([lmargin, bmargin, 1.0 - lmargin - rmargin, 1.0 - bmargin - tmargin])
    pylab.bar(xlefts, subs, width=barwidth)
    pylab.bar(xlefts, indels, width=barwidth)
    pylab.gca().set_xlim([mutstart - 0.5, genelength + 0.5])
    yticker = matplotlib.ticker.MultipleLocator(1)
    pylab.gca().yaxis.set_major_locator(yticker)
    ymax = max(subs + indels)
    pylab.gca().set_ylim([-ymax, ymax])
    pylab.title(title, fontsize=12)
    pylab.xlabel('codon number')
    pylab.ylabel('indels <-> subst.')
    pylab.savefig(plotfile)
    time.sleep(0.5)
    pylab.show()
    # make cumulative distribution plot
    cumul = []
    for i in range(len(subs)):
        if cumul:
            cumul.append(cumul[-1] + subs[i] / float(nsubs))
        else:
            cumul.append(subs[i] / float(nsubs))
    pylab.figure(figsize=(4.5, 2.25))
    (lmargin, rmargin, bmargin, tmargin) = (0.13, 0.04, 0.21, 0.07)
    pylab.axes([lmargin, bmargin, 1.0 - lmargin - rmargin, 1.0 - bmargin - tmargin])
    linear = [(x-mutstart+1) / float(genelength-mutstart+1) for x in xs]
    pylab.plot(xs, cumul, 'r-', label='actual')
    pylab.plot(xs, linear, 'b--', label='uniform')
    pylab.gca().set_xlim([mutstart, genelength])
    pylab.gca().set_ylim([0, 1])
    pylab.ylabel('cumulative fraction')
    pylab.gca().yaxis.set_major_locator(matplotlib.ticker.FixedLocator([0, 0.5, 1]))
    pylab.xlabel('codon number')
    pylab.legend(loc='upper left')
    pylab.title(title, fontsize=12)
    pylab.savefig(cumulplotfile)
    time.sleep(0.5)
    pylab.show()
    pylab.clf()
    pylab.close()


def ReadClone(line, seq):
    """Parses information from a line specifying mutations for a clone.

    line -> line containing mutation information
    seq -> the wildtype sequence.
    Returns the following tuple: (name, mutations, indels)
        name -> string giving name of clone
        mutations -> list of 3-tuples of wildtype codon, codon number, mutant codon
        indels -> list of codon numbers of sites where indels occur
    """
    submatch = re.compile('^(?P<wt>[ATCG]+)(?P<num>\d+)(?P<mut>[ATCG]+)$')
    indelmatch = re.compile('(ins|del)([ATCG]+)(?P<num>\d+)')
    (name, muts) = line.split(':')
    muts = muts.split(',')
    if len(muts) == 1 and muts[0].strip() == 'None':
        return (name, [], [])
    mutations = []
    indels = []
    for mutstring in muts:
        mutstring = mutstring.strip()
        if not mutstring:
            raise ValueError("No mutations specified for a clone. If a clone has no mutations, enter None in the mutation list.")
        m = submatch.search(mutstring)
        if m:
            (wt, num, mut) = (m.group('wt'), int(m.group('num')), m.group('mut'))
            assert len(wt) == len(mut)
            if seq[num - 1 : num - 1 + len(wt)] != wt:
                raise ValueError("Mismatch at %s. Make sure you entered this mutation correctly -- probably you entered the wrong wildtype identity or misnumbered the mutation." % mutstring)
            (icodon, ipos) = divmod(num - 1, 3)
            icodon = icodon + 1
            if ipos + len(wt) > 3:
                raise ValueError("Mutation spans multiple codons")
            wtcodon = seq[3 * (icodon - 1) : 3 * icodon]
            assert wt in wtcodon
            mutcodon = list(wtcodon)
            for i in range(len(mut)):
                mutcodon[i + ipos] = mut[i]
            mutations.append((wtcodon, icodon, mutcodon))
        else:
            m = indelmatch.search(mutstring)
            if not m:
                raise ValueError("Couldn't match sub or indel: %s" % mutstring)
            num = int(m.group('num'))
            (icodon, ipos) = divmod(num - 1, 3)
            icodon = icodon + 1
            indels.append(icodon)
    return (name, mutations, indels)


def main():
    """Main body of script."""

    print "\nBeginning analysis."
    seqfile = raw_input("\nEnter the name of the FASTA file containing the gene sequence: ").strip()
    if not os.path.isfile(seqfile):
        raise IOError("Cannot find specified file %s" % seqfile)
    seq = open(seqfile).readlines()[1].strip().upper()
    print "Read a coding sequence of length %d" % len(seq)
    assert len(seq) % 3 == 0, "Sequence length is not a multiple of three."
    ncodons = len(seq) // 3

    # read sequence
    mfile = raw_input("\nEnter the name of the file containing the list of mutations: ").strip()
    if not os.path.isfile(mfile):
        raise IOError("Cannot find specified file %s" % mfile)
    
    # get the position of the first codon in the mutated portion of the gene
    mutstart = int(raw_input("\nEnter the position of the first codon in the mutated segment of the gene: ").strip())
    
    # begin looping over input libraries
    print "\nReading mutations from %s" % mfile
    clones = [line for line in open(mfile).readlines() if (not line.isspace()) and line[0] != '#']
    print "Read entries for %d clones" % len(clones)
    clone_d = {}
    sub_nums = []
    indel_nums = []
    nmutations = []
    allmutations = []
    mutation_nums_by_clone = []
    for line in clones:
        (name, mutations, indels) = ReadClone(line, seq)
        if name in clone_d:
            raise ValueError("duplicate clone %s" % name)
        clone_d[name] = True
        nmutations.append(len(mutations))
        allmutations += mutations
        imutation_nums_by_clone = []
        for (wtcodon, icodon, mutcodon) in mutations:
            if icodon < mutstart:
                raise ValueError("This line reports a mutation before the beginning of the mutated segment of the gene: %s" %line)
            imutation_nums_by_clone.append(icodon)
            sub_nums.append(icodon)
        mutation_nums_by_clone.append(imutation_nums_by_clone)
        for icodon in indels:
            if icodon < mutstart:
                raise ValueError("This line reports an indel before the beginning of the mutated segment of the gene: %s" %line)
            indel_nums.append(icodon)
    sub_nums.sort()
    indel_nums.sort()
    print "\nSubstitutions begin at following positions: %s" % ', '.join([str(s) for s in sub_nums])
    print "\nIndels begin at following positions: %s" % ', '.join([str(s) for s in indel_nums])
    denom = float((ncodons - mutstart + 1) * len(clones))
    print "\nFound a total of %d substitutions out of %d codons sequenced (%.4f)" % (len(allmutations), (ncodons - mutstart + 1) * len(clones), len(allmutations) / denom)
    n_nmuts = {1:0, 2:0, 3:0}
    n_muttypes = {'synonymous':0, 'nonsynonymous':0, 'stop codon':0}
    for (wt, i, m) in allmutations:
        ndiffs = len([i for i in range(3) if wt[i] != m[i]])
        n_nmuts[ndiffs] += 1
        m = ''.join(m)
        wtaa = TranslateCodon(wt)
        mutaa = TranslateCodon(m)
        if wtaa == mutaa:
            n_muttypes['synonymous'] += 1
        elif wtaa and not mutaa:
            n_muttypes['stop codon'] += 1
        else:
            n_muttypes['nonsynonymous'] += 1
    print "\nHere are the fractions with different numbers of nucleotide mutations:"
    for n in range(1, 4):
        print "  %d nucleotide mutations: %.5f" % (n, n_nmuts[n] / denom)
    print "\nHere are the fractions of mutation types"
    for key in ['synonymous', 'nonsynonymous', 'stop codon']:
        print "  %s: %.5f" % (key, n_muttypes[key] / denom)
    nclones = len(clone_d)
    print "\nOverall summary:\n%d clones, avg. %.1f codon substitutions, avg. %.1f indels" % (nclones, len(sub_nums) / float(nclones), len(indel_nums) / float(nclones))
    print "\nNow creating the output PDF plot files..."
    title = ''
    PlotGeneMutDist(ncodons, sub_nums, indel_nums, "mutpositions.pdf", "mutpositions_cumulative.pdf", title, mutstart)
    os.system('convert -density 150 mutpositions.pdf mutpositions.jpg')
    os.system('convert -density 150 mutpositions_cumulative.pdf mutpositions_cumulative.jpg')
    PlotNMutDist(nmutations, 'nmutdist.pdf', '')
    os.system('convert -density 150 nmutdist.pdf nmutdist.jpg')
    PlotNCodonMuts(allmutations, 'ncodonmuts.pdf', '')
    os.system('convert -density 150 ncodonmuts.pdf ncodonmuts.jpg')
    PlotCodonMutNTComposition(allmutations, 'codonmutntcomposition.pdf', '')
    os.system('convert -density 150 codonmutntcomposition.pdf codonmutntcomposition.jpg')
    PlotMutationClustering(mutation_nums_by_clone, ncodons, 'mutationclustering.pdf', '', mutstart)
    os.system('convert -density 150 mutationclustering.pdf mutationclustering.jpg')
    print "The output PDF file plots have now all been created.\n\nScript complete."


main() # run the script
```

```
Overwriting analyze_library.py
```

## Execute the analysis¶

Command-line arguments to execute the analysis. The first line initiates the script. The next three lines are input to the prompts raised by the script, including name of fasta file, name of the file containing the list of mutations, and the first codon in the mutated segment of the gene. The results of the script are shown below:

In [4]:

```
%%bash
python analyze_library.py
LAI-Env.fasta
mut_list.txt
31
```

```
Beginning analysis.

Enter the name of the FASTA file containing the gene sequence: Read a coding sequence of length 2121

Enter the name of the file containing the list of mutations: 
Enter the position of the first codon in the mutated segment of the gene: 
Reading mutations from mut_list.txt
Read entries for 26 clones

Substitutions begin at following positions: 46, 54, 70, 77, 91, 114, 147, 154, 191, 192, 241, 242, 244, 252, 261, 269, 270, 290, 318, 385, 386, 409, 413, 428, 450, 469, 471, 473, 507, 537, 566, 586, 586, 595, 604, 613, 629

Indels begin at following positions: 98, 115, 516

Found a total of 37 substitutions out of 17602 codons sequenced (0.0021)

Here are the fractions with different numbers of nucleotide mutations:
  1 nucleotide mutations: 0.00062
  2 nucleotide mutations: 0.00097
  3 nucleotide mutations: 0.00051

Here are the fractions of mutation types
  synonymous: 0.00011
  nonsynonymous: 0.00199
  stop codon: 0.00000

Overall summary:
26 clones, avg. 1.4 codon substitutions, avg. 0.1 indels

Now creating the output PDF plot files...
The output PDF file plots have now all been created.

Script complete.
```

In [ ]:

```

```
