# Supplementary material for: Experimental Estimation of the Effects of All Amino-Acid Mutations to HIV’s Envelope Protein on Viral Replication in Cell Culture
Source: PLoS Pathog. 2016 Dec 13;12(12):e1006114. doi: 10.1371/journal.ppat.1006114 (PMC5189966; doi:10.1371/journal.ppat.1006114)
Supplement: S3 File — (ZIP) [file ppat.1006114.s013.zip › S3_File_notebooks/secondary_structure_and_RSA_analysis/secondary_structure_and_RSA_analysis.html]

secondary\_structure\_and\_RSA\_analysis


# Analyzing Env's secondary structure and relative solvent accessibility¶

I use `dssp` (http://swift.cmbi.ru.nl/gv/dssp/) to assign residues to secondary structures and to compute the relative solvent accessibility based on a recent crystal structure (PDB: 4TVP) by Pancera et al. (http://www.nature.com/nature/journal/v514/n7523/full/nature13808.html)

Hugh Haddox, March-8-2016

## Imports¶

In [1]:

```
import os
import mapmuts.dssp
import matplotlib
matplotlib.use('pdf')
%matplotlib inline
import pylab
import doctest
```

## Installing `dssp`¶

I installed `dssp` version 2.2.1 (http://swift.cmbi.ru.nl/gv/dssp/) using the commands:

> wget ftp://ftp.cmbi.ru.nl:21/pub/software/dssp/dssp-2.2.1.tgz
>
> tar -xvzf dssp-2.2.1.tgz
>
> cd dssp-2.2.1/

Next, in "makefile" I changed "DEST\_DIR ?= /usr/local" to "DEST\_DIR ?= /home/hhaddox/.local"

Next, I completed the installation with the following commands:

> make
>
> make install

## Use the `dssp` program to assign residues to secondary-structural elements and to compute absolute solvent accessibility for each residue in the 4tvp Env crystal structure¶

First, I will make some changes to the 4tvp biological assembly pdb file:

- I will first remove the antibody-associated chains.
- Next, I will rename the chains corresponding to gp120 and gp41 (chains G and B, respectively) in each timer, giving each chain a unique name. This step is necessary since, for some reason, dssp seems to only analyze monomers in context of the full trimer if each monomer is named differently. I will do this by executing the below script *make\_4tvp\_pdb\_file\_for\_dssp.py* in the PyMOL terminal using the command:

> run make\_pdb\_file\_for\_dssp.py

This command should generate the new .pdb file: *4tvp\_renamed\_chains.pdb*

In [2]:

```
%%writefile make_4tvp_pdb_file_for_dssp.py

"""
This script is for making a 4tvp .pdb file that can serve as input to the dssp program
for computing RSA.

It is intended to be run in the PyMOL terminal as:
    run make_pdb_file_for_dssp.py

Hugh Haddox, January-28-2016
"""
import pymol
from pymol import cmd

cmd.delete('all')
cmd.fetch('4tvp', type='pdb1')
cmd.remove ('c;d,e,h,l')
cmd.split_states('4tvp')
s1 = '4tvp_0001'
s2 = '4tvp_0002'
s3 = '4tvp_0003'
cmd.alter('4tvp_0002 and chain B', 'chain="W"')
cmd.alter('4tvp_0002 and chain G', 'chain="X"')
cmd.alter('4tvp_0003 and chain B', 'chain="Y"')
cmd.alter('4tvp_0003 and chain G', 'chain="Z"')

cmd.save('4tvp_renamed_chains.pdb', '4tvp_0001 and chain B + 4tvp_0001 and chain G + chain W + chain X + chain Y + chain Z')
```

```
Overwriting make_4tvp_pdb_file_for_dssp.py
```

Next, I will remove lines in the new pdb file that start with "TER" since these cause problems with the dssp program. These lines occur at boundaries of unresolved parts of primary sequence in the crystal structure. It does not seem like their removal should not affect how dssp determines absolute solvent accessibility. I will also remove lines corresponding to "HETATM", which `dssp` is said to ignore anyway (see http://swift.cmbi.ru.nl/gv/dssp/).

In [3]:

```
%%capture
# Specify names of input and output .pdb files
pdb_file_4tvp_name = '4tvp_renamed_chains.pdb'
pdb_file_4tvp = open(pdb_file_4tvp_name, 'r')
pdb_file_4tvp_rmTERandHETATM_name = '4tvp_renamed_chains_rmTERandHETATM.pdb'
pdb_file_4tvp_rmTERandHETATM = open(pdb_file_4tvp_rmTERandHETATM_name, 'w')

# Write a new .pdb file without lines starting with "TER"
print "Removing the following lines from .pdb file: %s"%pdb_file_4tvp_name
for line in pdb_file_4tvp:
    if 'TER' in line or 'HETATM' in line:
        print line.strip()
    else:
        pdb_file_4tvp_rmTERandHETATM.write(line)
pdb_file_4tvp_rmTERandHETATM.close()
```

Next, I will use the `dssp` program to analyze the 4tvp structure. Among other things, the output includes secondary-structure assignments for each residue along with "solvent accessibility" (ACC), which is defined as: "number of water molecules in contact with this residue \*10. or residue water exposed surface in Angstrom\*\*2" (see http://swift.cmbi.ru.nl/gv/dssp/).

In [5]:

```
# Run dssp
print "A .dssp file will be generated using:"
!mkdssp --version

dssp_4tvp_file_name = '4tvp_renamed_chains_rmTERandHETATM.dssp'

cmd = ' '.join([
        'mkdssp',
        '-i %s'%pdb_file_4tvp_rmTERandHETATM_name,
        '-o %s'%dssp_4tvp_file_name])

print("\nMaking a .dssp file with the command:\n" + cmd)

!$cmd
```

```
A .dssp file will be generated using:
mkdssp version 2.2.1

Making a .dssp file with the command:
mkdssp -i 4tvp_renamed_chains_rmTERandHETATM.pdb -o 4tvp_renamed_chains_rmTERandHETATM.dssp
```

## Create a file with per-residue secondary-structure annotations. Also, compute per-residue relative solvent accessibility based on solvent accessibility values from .dssp files¶

Next, I will use the `dssp.py` script from `mapmuts` (http://jbloom.github.io/mapmuts/) to extract per-residue secondary-structure information and ACC (solvent accessibility) values from the dssp file. I will write the secondary structure information to a file. I will then convert the ACC values to values of relative solvent accessibility (RSA) and write those to a file. I will use `mapmuts` to compute RSA by normizing ACC values for each residue by that residue's maximum accessible solvent area (ASA), as given in Tien et al, Maximum allowed solvent accessibilities of residues in proteins, PLOS ONE, 2013, as defined in Table 1 in the column labeled "Theoretical" at http://www.plosone.org/article/info:doi/10.1371/journal.pone.0080635 .

Since the `mapmuts` script doesn't work if there are sites with letters, I will first remove site "321A":

In [6]:

```
dssp_4tvp_file = open(dssp_4tvp_file_name, 'r')
dssp_4tvp_rm_321A_file_name = '4tvp_renamed_chains_rmTERandHETATM_rm321A.dssp'
dssp_4tvp_rm_321A_file = open(dssp_4tvp_rm_321A_file_name, 'w')

print "Removing the following lines from .dssp file: %s"%dssp_4tvp_file_name
for line in dssp_4tvp_file:
    if '321A' in line:
        print line.strip()
    else:
        dssp_4tvp_rm_321A_file.write(line)
dssp_4tvp_rm_321A_file.close()
```

```
Removing the following lines from .dssp file: 4tvp_renamed_chains_rmTERandHETATM.dssp
282  321AG D        -     0   0   90    -18,-0.1     2,-0.5     1,-0.1    -1,-0.2  -0.334  60.7-110.1 -71.3 157.3 -287.6 -150.8  -36.9
866  321AX D        -     0   0   90    -18,-0.1     2,-0.5     1,-0.1    -1,-0.2  -0.334  60.7-110.2 -71.3 157.3 -241.1 -173.6  -36.9
1450  321AZ D        -     0   0   89    -18,-0.1     2,-0.5     1,-0.1    -1,-0.2  -0.334  60.7-110.2 -71.2 157.3 -244.6 -122.0  -36.9
```

Now I will use the `dssp.py` script from `mapmuts` to read in information from the `.dssp` file, writing seperate output files with either per-site secondary structure or RSA values. These files have two space-deliminted columns, the first with the site and the second with the value. The top row has a '#' and labels for each column.

In [7]:

```
print "dssp files will be analyzed using mapmuts.dssp"

print "\nReading in information from the input .dssp file: %s" % dssp_4tvp_rm_321A_file_name
dssp_4tvp_G = mapmuts.dssp.ReadDSSP(dssp_4tvp_rm_321A_file_name, 'Tien2013', chain='G') #gp120 chain
dssp_4tvp_B = mapmuts.dssp.ReadDSSP(dssp_4tvp_rm_321A_file_name, 'Tien2013', chain='B') #gp41 chain
dssp_4tvp = {}
for i in dssp_4tvp_G:
    dssp_4tvp[i] = dssp_4tvp_G[i]
for j in dssp_4tvp_B:
    dssp_4tvp[j] = dssp_4tvp_B[j]
```

```
dssp files will be analyzed using mapmuts.dssp

Reading in information from the input .dssp file: 4tvp_renamed_chains_rmTERandHETATM_rm321A.dssp
```

In [8]:

```
# First I will write secondary structure to a file
secondary_structure_4TVP_file_name = 'secondary_structure_4tvp_renamed_chains_rmTERandHETATM_rm321A.txt'
print "\nWriting secondary-structure information to the output .txt file: %s" %secondary_structure_4TVP_file_name
secondary_structure_4TVP_file = open(secondary_structure_4TVP_file_name, 'w')
secondary_structure_4TVP_file.write('# POSITION secondary_structure\n')
sites = dssp_4tvp.keys()
sites.sort
for site in sites:
    secondary_structure_4TVP_file.write('%s %s\n' %(site, dssp_4tvp[site]['SS_CLASS']))
secondary_structure_4TVP_file.close()
```

```
Writing secondary-structure information to the output .txt file: secondary_structure_4tvp_renamed_chains_rmTERandHETATM_rm321A.txt
```

In [9]:

```
# Next, I will write the site-specific RSA values to a file after normalizing the values so that the higest RSA value
# equals one
RSA_4TVP_file_name = 'RSA_4tvp_renamed_chains_rmTERandHETATM_rm321A.txt'
print "\nWriting RSA values to the output .txt file: %s" %RSA_4TVP_file_name
RSA_4TVP_file = open(RSA_4TVP_file_name, 'w')
RSA_4TVP_file.write('# POSITION RSA\n')

# Normalize the values so that the max value = 1
RSA_values = [dssp_4tvp[site]['RSA'] for site in dssp_4tvp]
max_RSA_value = max(RSA_values)
print "The max RSA value is: %s"%max_RSA_value
print "Normalizing RSA values by diving by this max value"
sites = dssp_4tvp.keys()
sites.sort()

for site in sites:
    RSA_4TVP_file.write('%s %s\n' %(site, dssp_4tvp[site]['RSA']/max_RSA_value))
RSA_4TVP_file.close()
```

```
Writing RSA values to the output .txt file: RSA_4tvp_renamed_chains_rmTERandHETATM_rm321A.txt
The max RSA value is: 1.14193548387
Normalizing RSA values by diving by this max value
```

## Plot the distribution of RSA values along Env's primary sequence and the cumulative distribution¶

In [10]:

```
def PlotSiteSpecificValues(site_values, value_type):
    """
    This function plots site-specific values as a function of primary sequence. It also plots the cumulative
    distribution of those values.
    
    Input:
        *site_values* : a dictionary keyed first by replicate (string or integer) and then by sites (integer),
        with site-specific values as the ultimate values.
        
        *value_type* : will be used as the label for the y axis in the plot of site-specific value and as the
        label for the x axis in the cumulative fraction plot
    """
    
    cumulative_distribution_data = []
    
    for rep in site_values:
        print "Plotting data for replicate: %s"%rep
        
        # Make lists of sites and corresponding site_values
        sites = site_values[rep].keys()
        sites.sort()
        values = [site_values[rep][site] for site in sites]

        # I will then plot site-specific ratios
        pylab.figure(figsize=(15, 5))
        ax = pylab.axes()
        # ax.set_yscale('log')
        # pylab.ylim([0.01, 100])
        pylab.xlim([31, 707])
        pylab.plot(sites, values, linewidth = 2)
        pylab.plot((31, 707), (1, 1), 'g--', linewidth = 2)
        ylabel = value_type
        xlabel = 'codon position'
        ax.set_ylabel(ylabel, fontsize=30)
        ax.set_xlabel(xlabel, fontsize=30)
        pylab.yticks(fontsize=20)
        pylab.xticks(fontsize=20)
        pylab.show()
        pylab.clf()
    
        # Next, I will compute the cumulative distribution of site-specific ratios
        n = float(len(sites))
        values.sort()
        cumulataive_dist_xvalues = values
        cumulataive_dist_yvalues = [(y+1.0)/n for y in range(len(sites))]
    
        cumulative_distribution_data.append((cumulataive_dist_xvalues, cumulataive_dist_yvalues, rep))
        print "\ncumulative_frac\tRSA"
        print "%s\t%s"%(cumulataive_dist_yvalues[int(0.1*n)], cumulataive_dist_xvalues[int(0.1*n)])
        print "%s\t%s"%(cumulataive_dist_yvalues[int(0.9*n)], cumulataive_dist_xvalues[int(0.9*n)])
    
    # I will then plot the cumulative distribution of site-specific ratios
    pylab.figure(figsize=(15,5))
    ax = pylab.axes()
    for (xvalues, yvalues, rep) in cumulative_distribution_data:
        pylab.plot(xvalues, yvalues, label = rep, linewidth = 2)
    # pylab.plot((1, 1), (0, 1), 'k--')
    # pylab.xlim([0, 3])
    ylabel = 'Frac. < this %s'%value_type
    xlabel = value_type
    ax.set_ylabel(ylabel, fontsize=30)
    ax.set_xlabel(xlabel, fontsize=30)
    pylab.yticks(fontsize=20)
    pylab.xticks(fontsize=20)
    pylab.legend(loc = 0, fontsize=25)
    pylab.show()
    pylab.clf()
    
    return None
```

In [11]:

```
plot_RSA_4tvp = {}
plot_RSA_4tvp[1] = dict((site, dssp_4tvp[site]['RSA']/max_RSA_value) for site in dssp_4tvp.keys())
value_type = 'RSA'
PlotSiteSpecificValues(plot_RSA_4tvp, value_type)
```

```
Plotting data for replicate: 1
```

```
cumulative_frac	RSA
0.100346020761	0.00435674733676
0.901384083045	0.502647513364
```

```
<matplotlib.figure.Figure at 0x7f16f1c8a1d0>
```

```
<matplotlib.figure.Figure at 0x7f16f1bd1c50>
```

In [ ]:

```

```
